# Socio-structural and individual determinants of HIV/STI prevention behaviors in Chile: a national cross-sectional analysis

**DOI:** 10.1186/s12889-026-26934-z

**Published:** 2026-03-12

**Authors:** Giuliano Duarte-Anselmi, Susana Sanduvete-Chaves, Daniel López-Arenas, Salvador Chacón-Moscoso

**Affiliations:** 1 Facultad de Psicología, Universitat de Barcelona, Barcelona, Spain; 2 Escuela de Obstetricia. Facultad de Ciencias Médicas, Universidad de Santiago de Chile, Av. Libertador Bernardo O`Higgins 3363, Estación Central, Santiago, Chile; 3 Facultad de Psicología, Universidad de Sevilla, Sevilla, Spain; 4 Universidad Autónoma de Chile, Santiago, Chile

**Keywords:** HIV prevention, socio-structural determinants, STI testing, COM-B model, injunctive norms

## Abstract

**Background:**

Despite ongoing prevention efforts, rates of consistent condom use and testing for human immunodeficiency virus (HIV)/sexually transmitted infections (STI) remain low in Chile. Understanding how individual and socio-structural behavioral determinants influence these prevention behaviors is essential to developing effective interventions.

**Methods:**

We analyzed nationally representative data from the ENSSEX 2022–2023 survey (*n* = 20,392), mapping 44 behavioral indicators to the COM-B model and the Theoretical Domains Framework. Multivariable logistic regression models assessed associations between behavioral determinants and two key outcomes: consistent condom use and HIV/STI testing, adjusted by sex and other covariates.

**Results:**

Consistent condom use was reported by 15.5% of respondents, and HIV/STI testing by 23.1%. Injunctive norms favoring condom use were strongly associated with consistent condom use (aOR = 1.89), while access to sexual health services (aOR = 2.07) and PrEP awareness (aOR = 1.67) were strongly associated with HIV/STI testing. General knowledge about HIV was not independently associated with either behavior after adjustment.

**Conclusions:**

In this cross-sectional study of a nationally representative sample of Chilean adults, socio-structural factors—particularly injunctive norms and access to sexual health services—showed stronger and more consistent associations with condom use and HIV/STI testing than did knowledge-related factors after multivariable adjustment. These findings highlight the need for comprehensive, culturally tailored, multi-component interventions that integrate structural, normative, and motivational elements to enhance HIV/STI prevention in Chile and similar settings.

**Trial registration:**

Not applicable.

**Supplementary Information:**

The online version contains supplementary material available at 10.1186/s12889-026-26934-z.

## Key Messages

⦁ Effective HIV/STI prevention requires combining individual skills, socio-structural supports, and normative change.

⦁ Consistent condom use and HIV/STI testing remain low in Chile despite national prevention efforts.

⦁ Socio-structural determinants—including injunctive norms, access to services, and social support—exert a stronger influence on prevention behaviors than knowledge alone.

⦁ Reflective motivation and psychological capability are associated with prevention behaviors, but their effects differ by sex.

⦁ These findings identify priority targets for the design of culturally responsive behavioral interventions.

**Supplementary Information:**

The online version contains supplementary material available at 10.1186/s12889-026-26934-z.

## Introduction

Sexually transmitted infections (STIs), including human immunodeficiency virus (HIV), continue to pose a major public health burden globally, with over one million new infections reported daily [1]. In Chile, key preventive behaviors—such as consistent condom use and regular HIV/STI testing—remain below desired levels despite sustained national prevention efforts [2, 3]. This behavioral gap underscores the need to understand not only what preventive actions people take but also the underlying factors that shape their behavior [1, 2, 4, 5].

Behavioral science provides robust frameworks for examining the cognitive, emotional, social, and environmental determinants that shape health-related decision-making [6]. The COM-B model [7] and the Theoretical Domains Framework (TDF) [8, 9] are among the most widely applied frameworks in behavioral and implementation research. These complementary models enable the systematic identification of modifiable factors that facilitate or hinder behavior change. According to COM-B, behavior arises from the interaction of capability (knowledge/skills, behavioral regulation), opportunity (resources, context, social norms), and motivation (beliefs, attitudes, emotions, identity).

The TDF expands on the core COM-B constructs, defining 14 domains that enable a more detailed analysis of behavioral influences [8, 10]. Together with the Behavior Change Wheel (BCW) and the AACTT (Action, Actor, Context, Target, Time) framework, these models allow for the systematic identification of barriers and enablers and the design of tailored interventions [11–13].

In line with the emphasis on determinants of behavior in these frameworks, meta-analytic evidence confirms that behavioral determinants are strongly associated with HIV/STI preventive behaviors. While early systematic reviews based on the theories of planned behavior demonstrated substantial variance in condom use [14], subsequent research has shown that interventions targeting motivation, opportunity, and social influence—rather than knowledge alone—prove more effective [15]. Recent meta-analyses and narrative reviews further emphasize the critical role of socio-structural determinants, including access to services, social support, and injunctive norms (perceived social approval or disapproval), as key drivers of sustained prevention behaviors [16–19]. This distinction provides a useful conceptual framework for structuring the present analysis of HIV/STI prevention behaviors [20].

Despite these advances, most existing evidence derives from high-income countries. Few studies in Latin America have systematically applied behavioral frameworks to population-level data. The Chilean National Health, Sexuality and Gender Survey (ENSSEX, 2022–2023) [21] offers a unique opportunity to address this gap. In a previous study, we mapped 44 ENSSEX indicators to COM-B and TDF domains, identifying barriers and enablers of condom use and HIV/STI testing [22]. There is evidence that knowledge alone rarely suffices to drive behavior change; structural factors such as access to services and supportive norms consistently demonstrate stronger associations [15, 16, 19].Distinguishing between individual and socio-structural determinants within ENSSEX data therefore provides both theoretical clarity and practical insights into the most relevant behavioral targets for HIV/STI prevention in Chile.

Building on this earlier qualitative-theoretical work, the present study adopts a combined COM-B and TDF framework to distinguish between individual determinants (e.g., knowledge, beliefs, self-efficacy) and socio-structural determinants (e.g., injunctive norms, access to resources, social support). Using survey-weighted multivariable analyses, we quantify and compare the strength of their associations with consistent condom use and HIV/STI testing in a nationally representative sample. By structuring our analysis around these domains, we aim to clarify the relative contribution of each and to identify the most strategic leverage points for intervention. Furthermore, in keeping with the Sex and Gender Equity in Research (SAGER) guidelines [23], we examine gender differences to generate culturally grounded and contextually relevant evidence for intervention design. Specifically, the aim of this study was to identify individual and socio-structural determinants associated with consistent condom use and HIV/STI testing within the past 12 months among Chilean adults, accounting for sex differences.

## Methods

### Study design and sample

The data for this cross-sectional study were drawn from the National Health, Sexuality, and Gender Survey (Encuesta Nacional de Salud, Sexualidad y Género-ENSSEX) [21]. The study was conducted between 2022 and 2023 by the Pontificia Universidad Católica de Chile under the coordination of the Chilean Ministry of Health. Adults aged 18 years or older who participated in the ENSSEX 2022–2023 survey and resided in urban areas of Chile met the inclusion criteria, while individuals residing in rural areas, populations not covered by the ENSSEX sampling frame (i.e., individuals not living in private households, such as institutionalized populations), and respondents who did not provide data on the primary outcomes of interest did not. These criteria reflect the original ENSSEX study design and sampling procedures.

Data collection took place between August and December 2022 using computer-assisted personal interviews (CAPI). The survey employed a multistage, stratified probability sampling design to ensure representativeness of the adult population (≥ 18 years) residing in Chile’s urban areas. The final dataset included 20,392 participants, yielding a weighted sample representative of approximately 13.4 million adults. To enhance analytic precision in age-disaggregated analysis, participants aged 18–34 and over 60 were intentionally oversampled.

### Ethics approval and informed consent

The Ethics Committee of the Faculty of Medicine at Pontificia Universidad Católica de Chile reviewed and approved the ENSSEX study protocol (Protocol ID: 211102002). All participants provided written informed consent prior to participation, in accordance with the Declaration of Helsinki and relevant national guidelines. The present study used anonymized, publicly available secondary data from ENSSEX 2022–2023 and did not require additional ethical approval.

### Measures

The ENSSEX questionnaire comprised 305 items grouped into 11 modules [21]. For this study, we analyzed a theory-driven subset of 44 items drawn from multiple modules: normative orientations (Module C), socialization and sexual education (Module D), sexual trajectory (Module E), sexual and reproductive health (Module I), HIV/STI knowledge and prevention (Module I and the HIV submodule), and socioeconomic characterization (Module K).

### Variable selection and theoretical mapping

Building on our previous deductive analysis of the ENSSEX 2022–2023 survey [22], these 44 items were systematically mapped to the COM-B and TDF domains. Items were classified using theory-driven thresholds as enablers, moderate barriers, major barriers, or descriptive variables only. This procedure achieved high inter-rater reliability (Cohen’s κ = 0.96) and provided the foundation for selecting variables for inferential modeling (see Supplementary Tables S1-S4).

In addition, following behavioral science theory (Cialdini et al., 1990 [24]; Albarracín et al., 2024 [16]), we distinguished between descriptive norms (behaviors reflecting what is commonly practiced) and injunctive norms (beliefs or expectations about what ought to be done).

### Outcomes

Two primary HIV/STI prevention behaviors were assessed:

1. Consistent condom use: measured by self-reported frequency of condom use in the past 12 months. Responses were dichotomized as “always” versus all other categories (“almost always,” “sometimes,” “never”).
2. HIV/STI testing: measured by whether participants reported having taken at least one HIV or STI test in the past 12 months (yes vs. no).

For the analysis of condom use (P73), the analytic denominator was restricted to respondents eligible for the item, defined as those who reported sexual activity in the past 12 months. In contrast, the analysis of HIV/STI testing (P208) included all survey respondents.

### Behavioral determinants

Determinants were organized into individual and socio-structural factors (see Table 1). Individual factors included HIV knowledge, measured as a composite score of correct responses to questions about HIV transmission, as well as beliefs and attitudes about condoms, such as whether condoms reduce the sexual pleasure of women or men, and whether they make sexual activity more playful. Self-efficacy was assessed in a question on whether respondents used condoms specifically to prevent HIV or STIs. Socio-structural determinants comprised injunctive norms, such as the belief that condoms should be used even with a stable partner; perceived material barriers, like the belief that condoms are too expensive for regular use; and access to services, measured by a respondent’s history of consulting a health professional about sexual health concerns. Social support was assessed using an index that combined family communication about sexuality during childhood and partner communication about prevention before first sex. Finally, awareness of pre-exposure prophylaxis (PrEP) was assessed as an additional determinant.

**Table 1:** Outcomes, determinants, and covariates: questionnaire items, coding, and response categories from ENSSEX 2022–2023

Domain	Variable (ENSSEX code)	Questionnaire item (English translation)	Response categories (numeric codes)	TDF Domain	COM sub-constructs	COM-B
Outcomes	Condom use (P73; reinforced with P89/P119)	“En las relaciones con esas parejas sexuales del último año, ¿con qué frecuencia usted usaba condón o preservativo?” [In your sexual relationships over the past year, how often did you use condoms? ]	1 = Always; 2 = Sometimes; 3 = Never; 9 = Don’t know/ No answer	Behavioral regulation	Psychological Capability	Capability
	HIV/STI testing (P208)	“Por cualquier razón, ¿se ha hecho el examen del VIH o Sida en los últimos 12 meses?” [Have you had an HIV test for any reason in the last 12 months? ]	1 = Yes; 2 = No; 8 = Don’t know; 9 = No answer	Behavioral regulation	Psychological Capability	Capability
Determinants – Individual	HIV knowledge (i_1–i_6_P212)	“¿Puede reducirse el riesgo de transmisión del VIH…?” (seis ítems, ej.: “¿Puede una persona de aspecto saludable tener VIH?”) [“Can HIV transmission be reduced by…?” (six items, e.g., “Can someone who looks healthy have HIV?”)]	1 = Yes; 2 = No; 8 = Don’t know; 9 = No answer (summed 0–6 correct)	Knowledge	Psychological Capability	Capability
	PrEP awareness (P213)	“¿Conoce usted la medida ‘profilaxis de pre exposición’ o PREP como alternativa de prevención del VIH/Sida?” [Do you know about pre-exposure prophylaxis (PrEP) as an option for HIV/AIDS prevention? ]	1 = Yes; 2 = No; 8 = Don’t know; 9 = No answer	Knowledge	Psychological Capability	Capability
	Belief: condoms reduce women’s pleasure (i_1_P33)	“Según lo que usted cree, ¿qué tan de acuerdo está con que usar preservativos o condón disminuye el placer de las mujeres?” [Based on what you believe, how much do you agree or disagree that using condoms reduces women’s sexual pleasure? ]	1 = Strongly disagree; 2 = Disagree; 3 = Neutral; 4 = Agree; 5 = Strongly agree; 8 = Don’t know; 9 = No answer	Beliefs about consequences	Reflective motivation	Motivation
	Belief: condoms reduce men’s pleasure (i_2_P33)	“Según lo que usted cree, ¿qué tan de acuerdo está con que usar preservativos o condón disminuye el placer de los hombres?” [Based on what you believe, how much do you agree or disagree that using condoms reduces men’s sexual pleasure? ]	2 = Strongly disagree; 2 = Disagree; 3 = Neutral; 4 = Agree; 5 = Strongly agree; 8 = Don’t know; 9 = No answer	Beliefs about consequences	Reflective motivation	Motivation
	Belief: condoms make sexual activity more playful (i_4_P33)	“Según lo que usted cree, ¿usar preservativo o condón estimula el juego sexual?” [Based on what you believe, does using condoms make sexual activity more playful? ]	3 = Strongly disagree; 2 = Disagree; 3 = Neutral; 4 = Agree; 5 = Strongly agree; 8 = Don’t know; 9 = No answer	Beliefs about consequences	Reflective motivation	Motivation
	Self-efficacy proxy (P121a)	“¿Por qué motivo usaron preservativo o condón? Para prevenir una ITS” [What was the reason for using a condom? To prevent an STI]	1 = Yes; 2 = No; 8 = Don’t know; 9 = No answer	Goals	Reflective motivation	Motivation
Determinants – Socio-structural	Injunctive norm (i_3_P33)	“Según lo que usted cree, ¿es necesario ocupar preservativo o condón incluso si se tiene pareja estable?” [Based on what you believe, do you agree or disagree that condoms should be used even when you are in a stable relationship? ]	1 = Strongly disagree; 2 = Disagree; 3 = Neutral; 4 = Agree; 5 = Strongly agree; 8 = Don’t know; 9 = No answer	Social influences	Social opportunity	Opportunity
	Perceived cost barrier (i_5_P33)	“Según lo que usted cree, ¿los preservativos o condones son demasiado caros para usarlos regularmente?” [Based on what you believe, do you agree or disagree that condoms are too expensive to use regularly? ]	2 = Strongly disagree; 2 = Disagree; 3 = Neutral; 4 = Agree; 5 = Strongly agree; 8 = Don’t know; 9 = No answer	Environmental context and resources	Physical opportunity	Opportunity
	Access to services (P151/P152)	“¿Alguna vez en su vida ha ido a una consulta o donde algún profesional de la salud…?” [Have you ever visited a health professional for personal medical concerns related to gynecology/urology/sexuality/contraception/STIs? ]	1 = Yes; 2 = No; 9 = No answer	Environmental context and resources	Physical opportunity	Opportunity
	Social support index (P34 + P55)	p34: “Cuando usted era niño/a, ¿En su familia se conversaban temas sexuales?” [When you were a child, did your family talk about sex? ]; p55: “Antes de su primera relación sexual, ¿Usted y esa persona hablaron de cómo evitar una ITS…?” [Before having intercourse for the first time with your partner, did you talk about how to avoid STIs…?]	p34: 1 = No; 2 = Some topics; 3 = All topics; 8 = Don’t know; 9 = No answer. p55: 1 = Yes; 2 = No; 9 = No answer. Combined 0–2 index.	Social influences	Social opportunity	Opportunity
Covariates	Sex (P1)	“¿Cuál es su sexo asignado al nacer?” [What sex were you assigned at birth? ]	1 = Male; 2 = Female	NA	NA	NA
	Age (P4)	“¿Cuál es su edad (en años)?” [How old are you? ]	Continuous (open numeric)	NA	NA	NA
	Education (P5)	“¿Cuál es su nivel educacional más alto alcanzado?” [What is your highest level of education you have completed? ]	1 = Never attended → 17 = Postgraduate complete; 88 = Don’t know; 99 = No answer (recoded ≤ 8, 9–12, ≥ 13 years)	NA	NA	NA
	Marital status (P7)	“¿Cuál es su estado civil actual?” [What is your current marital status? ]	1 = Married; 2 = Cohabiting; 3 = Civil union; 4 = Annulled; 5 = Separated; 6 = Divorced; 7 = Widowed; 8 = Single; 88 = Don’t know; 99 = No answer	NA	NA	NA
	Sexual orientation (P134)	“Actualmente, ¿cómo se identifica?” [How do you currently identify? ]	1 = Gay; 2 = Lesbian; 3 = Bisexual; 4 = Heterosexual; 5 = Other; 6 = Prefer not to answer; 8 = Don’t know; 9 = No answer	NA	NA	NA
	Socioeconomic status (P285)	Índice socioeconómico ENSSEX derivado [Derived ENSSEX composite index]	Low; Medium; High	NA	NA	NA

Condom use was primarily assessed with item P73 on condom use over the past year. Items P89 (“Y en la primera relación sexual que volvió a tener con esa persona después de la separación, ¿usaron condón o preservativo?” [In the first sexual encounter you had with that person after the separation, did you use a condom?]). P119 (“En esa última relación sexual, ¿Ustedes usaron algún método anticonceptivo?” [In that last sexual encounter, did you use any contraceptive method?]) were included as auxiliary variables to validate consistency of reporting across time frames. P119 is not condom-specific and was used only as an auxiliary indicator and for population-level descriptive norms, not as an independent covariate.

Social norms were conceptualized as descriptive (reflecting what people commonly do) and injunctive (reflecting what is socially approved or expected). Because the survey did not measure perceived peer behavior, population-level prevalence indicators (e.g., condom use rates, contraceptive use at last sex, and family communication about sexuality) were used as a proxy for descriptive norms. These indicators were used descriptively to provide contextual benchmarks but were not entered as independent covariates. In contrast, injunctive norms were measured directly through beliefs about the need to use condoms, pleasure, and partner communication about prevention. This classification, summarized in Supplementary Table S2.1, follows Cialdini et al. (1990) [24] and Albarracín et al. (2024) [16].

### Covariates

Sociodemographic variables included sex (male/female), age (continuous, modeled using a natural cubic spline with 3 degrees of freedom), marital status (single, partnered, separated/divorced/widowed), educational attainment (≤ 8, 9–12, ≥ 13 years), sexual orientation (heterosexual, gay/lesbian, bisexual/other), and socioeconomic status (low, medium, high).

### Statistical analysis

The analysis accounted for the complex survey design of ENSSEX (weights, strata, and clusters) to allow generalization of the findings to the national adult population of urban Chile. Survey weights were constructed by the ENSSEX study team to correct for differential probabilities of selection and non-response, using a multistage design-based approach. Final weights incorporated sampling probabilities at each stage and were calibrated through post-stratification to match national population distributions by age, sex, and region, based on official census projections. These weights were applied in all descriptive and inferential analyses. In the descriptive analysis, weighted prevalence of outcomes were estimated and determinants were stratified by sex and age group. Bivariate associations were examined using survey-weighted chi-square tests and crude odds ratios (ORs). Multivariable analyses employed survey-weighted logistic regression models to estimate adjusted odds ratios (aORs) with 95% confidence intervals (CIs).

For condom use, the models included age, sex, injunctive norms, self-efficacy, condom-related beliefs, perceived cost, access to services, social support, HIV knowledge, education, sexual orientation, socioeconomic status, and marital status, with interaction terms testing whether associations differed by sex for injunctive norms and self-efficacy. For HIV/STI testing, the models included age, sex, HIV knowledge, PrEP awareness, access to services, social support, education, sexual orientation, socioeconomic status, and marital status, with interaction terms testing whether associations differed by sex for access and knowledge. Block-wise models were estimated sequentially (covariates → individual factors → socio-structural factors), with incremental fit assessed using ΔTjur’s R², ΔAIC, and Wald tests. Model performance was evaluated through the area under the curve (AUC) and Hosmer–Lemeshow tests. Primary analyses were based on complete-case models, with sensitivity analyses excluding variables with substantial missingness. Sex and gender were considered in descriptive analyses, while in multivariable models the most theoretically appropriate variable was retained, and the alternative was tested in sensitivity analyses in accordance with SAGER guidelines [23]. All analyses were conducted in R (version 2025.09.0 + 387) using survey-weighted regression functions (svyglm, quasibinomial family) and visualization packages. Full model specifications and supplementary results are provided in Appendices 5 and 6.

Following established normative frameworks, we classified ENSSEX items as descriptive and injunctive (prescriptive) norms. Descriptive norms were represented using survey-weighted estimates of the prevalence of relevant behaviors in the population and therefore were not entered as independent covariates. Item P73 was the primary outcome, while P119 was used only as an auxiliary indicator for temporal coherence. The full item classification and theoretical justification are provided in Supplementary Table S2.1.

## Results

### Overview of the dataset

The ENSSEX questionnaire comprises 305 items; including sub-items, 405 prompts were reviewed. Of these, 44 items were identified as relevant to condom use and HIV/STI testing and were deductively mapped to TDF domains and COM-B model, with AACTT used to define action, actor, context, target, and time (as previously described [22]). The represented TDF domains were environmental context and resources (11 items), knowledge (9), behavioral regulation (8), beliefs about consequences (5), social influences (4), beliefs about capabilities (3), goals (3), and reinforcement (1). Inter-rater agreement for TDF coding was Cohen’s κ = 0.96. Full details are provided in Supplementary Appendices 1–4.

### Participant characteristics and outcome prevalence

The analytic sample comprised 20,392 adults aged ≥ 18 years, representing approximately 13.4 million urban-dwelling adults after applying survey weights. The weighted sex distribution was 51.7% female and 48.3% male; age composition and other covariates (education, marital status, sexual orientation, and socioeconomic status) are shown in Table 2. Overall, 62.7% of respondents reported being sexually active in the past 12 months. In the eligible subpopulation for P73, the weighted prevalence of consistent (“always”) condom use was 15.5% (95% CI: 14.5–16.5). In the full sample, the weighted prevalence of ≥ 1 HIV/STI test in the past 12 months was 23.1% (95% CI: 22.1–24.0) (Table 2).

**Table 2:** Sample characteristics and weighted prevalence of outcomes (ENSSEX 2022–2023)

Category	Overall – *n* (unweighted %)	Overall – *n* (weighted %)	Condom use							HIV/STI testing		
			Over the past year, how often did you use condoms? (p73)			In your first sexual encounter after the separation, did you use a condom? (p89)		In your last sexual encounter, did you use any contraceptive method? (p119)		Have you had an HIV test for any reason in the last 12 months? (p208)		
			Always — *n* = 2236 15.5% (IC95%: 14.5–16.5)	Sometimes — *n* = 2620	Never — *n* = 7911	Yes — *n* = 801	No — *n* = 1427	Yes — *n* = 4467	No — *n* = 7644	Yes — *n* = 4004 23.1% (IC95%: 22.1–24.0	No — *n* = 13,720	
Sex												
Men	6838 (33.5)	6838 (48.3)	990; 21.7 (20.5–22.9)	1051; 23.0 (21.8–24.3)	2497; 54.7 (53.3–56.1)	327; 39.3 (36.0–42.6)	494; 59.3 (55.9–62.6)	1755; 38.1 (36.7–39.5)	2789; 60.5 (59.1–61.9)	1078; 17.6 (16.7–18.6)	4889; 79.8 (78.8–80.8)	
Women	13,554 (66.5)	13,554 (51.7)	1246; 15.0 (14.2–15.8)	1569; 18.9 (18.0–19.7)	5414; 65.1 (64.1–66.2)	474; 33.3 (30.9–35.8)	933; 65.5 (63.0–67.9)	2712; 35.5 (34.4–36.6)	4855; 63.6 (62.5–64.6)	2926; 24.4 (23.6–25.1)	8831; 73.6 (72.8–74.3)	
Age												
< 35	7346 (36.0)	7346 (34.6)	1474; 29.6 (28.3–30.9)	1509; 30.3 (29.0–31.6)	1971; 39.6 (38.2–40.9)	580; 44.3 (41.7–47.0)	707; 54.1 (51.3–56.7)	2868; 56.6 (55.2–58.0)	2153; 42.5 (41.1–43.9)	1743; 29.0 (27.8–30.1)	4128; 68.6 (67.4–69.8)	
35–59	7971 (39.1)	7971 (42.6)	684; 11.8 (11.0–12.7)	978; 16.9 (16.0–17.9)	4077; 70.5 (69.3–71.6)	208; 24.7 (21.9–27.7)	629; 74.6 (71.6–77.4)	1526; 27.6 (26.5–28.8)	3943; 71.4 (70.1–72.5)	1736; 23.2 (22.3–24.2)	5564; 74.5 (73.5–75.4)	
60+	5075 (24.9)	5075 (22.7)	78; 3.7 (3.0–4.6)	133; 6.3 (5.3–7.4)	1863; 88.3 (86.9–89.6)	13; 12.1 (7.2–19.7)	91; 85.0 (77.1–90.6)	73; 4.4 (3.5–5.5)	1548; 93.4 (92.1–94.5)	525; 11.3 (10.4–12.2)	4028; 86.7 (85.7–87.7)	
Education												
≤ 8 years	4720 (23.2)	4720 (19.4)	165; 7.2 (6.2–8.3)	264; 11.5 (10.3–12.9)	1815; 79.2 (77.4–80.8)	43; 21.4 (16.3–27.6)	154; 76.6 (70.3–81.9)	260; 14.3 (12.8–16.0)	1517; 83.5 (81.7–85.1)	556; 13.1 (12.1–14.2)	3564; 84.1 (83.0–85.2)	
9–12 years	8611 (42.3)	8611 (37.8)	862; 15.5 (14.6–16.5)	1148; 20.7 (19.7–21.8)	3510; 63.3 (62.0–64.6)	300; 32.5 (29.5–35.6)	617; 66.8 (63.7–69.7)	1881; 35.2 (33.9–36.4)	3404; 63.6 (62.3–64.9)	1665; 22.3 (21.4–23.2)	5636; 75.4 (74.4–76.4)	
≥ 13 years	7049 (34.6)	7049 (42.8)	1207; 24.0 (22.8–25.2)	1208; 24.0 (22.8–25.2)	2581; 51.3 (49.9–52.7)	458; 40.5 (37.7–43.4)	654; 57.8 (54.9–60.7)	2325; 45.8 (44.4–47.2)	2718; 53.5 (52.2–54.9)	1781; 27.8 (26.7–28.9)	4514; 70.4 (69.3–71.5)	
Marital status												
Partnered	7355 (36.2)	7355 (44.0)	457; 7.6 (7.0–8.3)	782; 13.0 (12.2–13.9)	4708; 78.6 (77.5–79.6)	120; 22.1 (18.8–25.8)	413; 76.2 (72.4–79.6)	1302; 23.7 (22.6–24.9)	4131; 75.2 (74.1–76.4)	1530; 21.9 (20.9–22.8)	5333; 76.2 (75.2–77.2)	
Separated/Divorced/Widowed	3837 (18.9)	3837 (15.1)	193; 13.2 (11.6–15.1)	275; 18.8 (16.9–20.9)	972; 66.6 (64.1–68.9)	75; 24.0 (19.6–29.0)	235; 75.1 (70.0–79.5)	352; 26.0 (23.7–28.4)	975; 71.9 (69.5–74.2)	576; 16.2 (15.0–17.4)	2913; 81.7 (80.4–82.9)	
Single	9129 (44.9)	9129 (40.9)	1580; 29.3 (28.1–30.6)	1550; 28.8 (27.6–30.0)	2212; 41.1 (39.8–42.4)	605; 43.2 (40.7–45.9)	776; 55.5 (52.9–58.1)	2802; 52.1 (50.8–53.5)	2519; 46.9 (45.5–48.2)	1888; 25.2 (24.2–26.2)	5421; 72.3 (71.3–73.3)	
Sexual orientation												
Bisexual/Other	1136 (5.6)	1136 (4.0)	148; 24.8 (21.5–28.5)	176; 29.5 (26.0–33.3)	263; 44.1 (40.2–48.1)	73; 43.2 (36.0–50.7)	96; 56.8 (49.3–64.0)	256; 43.3 (39.4–47.3)	314; 53.1 (49.1–57.1)	207; 26.8 (23.8–30.0)	523; 67.7 (64.4–70.9)	
Gay/Lesbian	282 (1.4)	282 (1.7)	63; 31.7 (25.6–38.4)	41; 20.6 (15.6–26.8)	93; 46.7 (39.9–53.7)	17; 37.8 (25.1–52.4)	27; 60.0 (45.5–73.0)	68; 33.0 (27.0–39.7)	133; 64.6 (57.8–70.8)	91; 38.4 (32.4–44.7)	140; 59.1 (52.7–65.1)	
Heterosexual	18,974 (93.0)	18,974 (94.4)	2025; 16.8 (16.1–17.4)	2403; 19.9 (19.2–20.6)	7555; 62.5 (61.7–63.4)	711; 34.8 (32.8–36.9)	1304; 63.8 (61.7–65.9)	4143; 36.2 (35.3–37.1)	7197; 62.8 (62.0–63.7)	3706; 21.6 (21.0–22.3)	13,057; 76.3 (75.6–76.9)	
Socioeconomic status												
Low	796 (29.2)	796 (25.6)	54; 12.4 (9.6–15.8)	71; 16.3 (13.1–20.1)	308; 70.8 (66.4–74.9)	27; 39.1 (28.5–50.9)	42; 60.9 (49.1–71.5)	98; 25.2 (21.1–29.7)	283; 72.8 (68.1–76.9)	179; 25.3 (22.3–28.7)	517; 73.1 (69.7–76.3)	
Middle	1528 (56.1)	1528 (59.5)	160; 16.9 (14.7–19.5)	151; 16.0 (13.8–18.5)	627; 66.4 (63.3–69.4)	54; 41.5 (33.4–50.1)	74; 56.9 (48.3–65.1)	305; 32.5 (29.6–35.6)	611; 65.1 (62.0–68.1)	270; 20.0 (17.9–22.2)	1027; 76.0 (73.7–78.2)	
High	401 (14.7)	401 (14.9)	47; 17.3 (13.3–22.2)	55; 20.2 (15.9–25.4)	164; 60.3 (54.4–65.9)	21; 36.8 (25.5–49.8)	34; 59.6 (46.7–71.4)	116; 42.3 (36.6–48.3)	155; 56.6 (50.6–62.3)	91; 24.8 (20.7–29.5)	264; 71.9 (67.1–76.3)	

Notes: (1) P73 (consistent condom use) was calculated in the eligible subpopulation reporting sexual activity in the past 12 months; unweighted *n* = 12,767; missing or “don’t know” responses were excluded (*n* = 110). (2) P208 (≥ 1 HIV/STI test in the past 12 months) was calculated in the full sample; unweighted *n* = 17,724; missing = 8; “don’t know” = 399. (3) Counts (n) are unweighted, whereas percentages and 95% confidence intervals (CIs) are weighted estimates derived using ENSSEX survey expansion factors. (4) 95% CIs account for the complex survey design (svy). (5) In the overall sample, the weighted prevalence of consistent condom use (P73) was 15.5% (95% CI: 14.5–16.5), and the weighted prevalence of ≥ 1 HIV/STI test in the past 12 months (P208) was 23.1% (95% CI: 22.1–24.0). (6) Data for P89 (“first sexual encounter after separation”) and P119 (“contraceptive use at last sexual encounter”) are also shown in the table but were not primary outcomes of this study.

Outcome prevalence varied by sex and age (Table 3). Consistent condom use was higher among participants aged < 35 years (30.2%) compared with those aged ≥ 60 years (3.4%), and was higher among men (20.0%) than among women (14.5%). In contrast, HIV/STI testing was more frequent among women (26.6%) than among men (19.3%), and declined with age (< 35: 29.2% vs. ≥60: 12.4%).

**Table 3:** Weighted prevalence by sex and age

Survey-weighted proportions (95% CI). Cell shows *n* (unweighted); % (95% CI)				
	Consistent condom use (p73)		HIV/STI testing (p208)	
Category	n (unweighted)	% (95% CI)	n (unweighted)	% (95% CI)
Sex				
Women	8229	14.5 (13.3–15.8)	11,757	26.6 (25.3–27.9)
Men	4538	20.0 (18.3–21.7)	5967	19.3 (17.6–21.0)
Age				
< 35	4954	30.2 (28.3–32.1)	5871	29.2 (27.4–30.9)
35–59	5739	11.9 (10.4–13.4)	7300	24.2 (22.6–25.9)
60+	2074	3.4 (2.2–4.6)	4553	12.4 (10.8–14.0)

Auxiliary consistency checks—first post-separation condom use (P89) and any contraceptive method at last sex (P119)—are presented in Supplementary Table S7 and were used to support temporal coherence; these items were not entered as independent covariates.

Taken together, these findings indicate that consistent condom use was most frequent among younger adults and among men, whereas HIV/STI testing was more frequent among women and decreased steadily with age.

### Distribution of behavioral determinants

Determinants exhibited heterogeneous distributions across domains (Table 4). Agreement with the injunctive norm that “condoms should be used even with a stable partner” (i_3_p33) was 50.6% (95% CI: 49.0–52.2). The perception that condoms are expensive (i_5_p33) was endorsed by 21.5% (20.3–22.7). PrEP awareness (p213) was relatively low (10.2%, 9.5–11.1). Regarding social support, the composite index combining family communication about sexuality during childhood (P34) and discussion with a partner before first sex (P55) showed a distribution of 57.3%, 31.7%, and 11.0% across the 0, 1, and 2 support categories, respectively (95% CIs: 55.9–58.7, 30.5–32.9, 10.2–11.8). Full breakdowns are available in Supplementary Table S8.

**Table 4:** Weighted prevalence of behavioral determinants (ENSSEX 2022–2023)

Determinant type	Variable	Indicator / Response	*n* (unweighted)	Weighted % (95% CI)
Individual determinants	HIV knowledge	Mean score (0–6), % correct items	19,916	64.3 (63.5–65.1)
	Condom belief: reduces women’s pleasure	Agree/strongly agree	16,288	31.0 (29.8–32.2)
	Condom belief: reduces men’s pleasure	Agree/strongly agree	16,096	43.4 (41.9–44.9)
	Condom belief: makes sexual activity more playful	Agree/strongly agree	16,103	30.7 (29.2–32.1)
	Self-efficacy	Used condom to prevent HIV/STIs	6,099	13.0 (11.6–14.6)
	PrEP awareness	Knows about PrEP	18,764	10.2 (9.5–11.1)
Socio-structural determinants	Injunctive norm	Condoms should be used even with stable partner (agree/strongly agree)	13,19	50.6 (49.0–52.2)
	Perceived cost barrier	Condoms are expensive (agree/strongly agree)	16,406	21.5 (20.3–22.7)
	Access to services	Ever consulted a health professional for sexual health	20,087	49.9 (48.1–51.6)
	Social support index	0 / 1 / 2	17,793	S0: 57.3(55.9–58.7) / S1: 31.7(30.5–32.9) / S2: 11.0 (10.2–11.8)

Notes: (1) Estimates are survey-weighted, and 95% confidence intervals account for the complex survey design (svy). (2) Unweighted cell counts (n) are shown per cell. (3) Self-efficacy (P121) is reported only among respondents eligible for the condom use items (unweighted *n* = 6,099). (4) Access to services combines P151 and P152 (ever consulted a health professional for sexuality, contraception, or STIs). (5) The social support index is defined as P34 (family communication about sex: any vs. none) plus P55 (partner prevention discussion before first sex); categories S0. S1, and S2 denote 0, 1, or 2 supportive components, respectively. (6) HIV knowledge is expressed as the mean percentage of correct responses across six items (equivalent to 3.86/6 correct)

### Social norms

Marked contrasts were observed in social norms (Supplementary Tables S9a–c). Among descriptive norms, only 15.5% of sexually active respondents reported always using condoms in the past year, whereas 36.0% who reported any contraceptive use at last sex and 68.8% recalled family discussions about sexuality during childhood. For injunctive norms, about half (50.6%) agreed that condoms should be used even with a stable partner, while roughly one in five endorsed beliefs that condoms reduce sexual pleasure and a similar proportion believed they enhance it; only 1.5% reported discussing STI prevention before first sex. When stratified by endorsement of the injunctive norm, respondents who agreed that condoms should be used even with a stable partner were more likely to report consistent condom use (20.5% vs. 9.0%) and HIV/STI testing in the past 12 months (26.9% vs. 21.3%).

### Individual and socio-structural determinants

Determinants were grouped into two categories: individual factors (primarily psychological capability and reflective motivation in COM-B, and knowledge, beliefs about consequences, and goals and behavioral regulation in TDF) and socio-structural factors (primarily social and physical opportunity in COM-B, and social influences and environmental context and resources in TDF). At the individual level, HIV knowledge averaged 64.3% correct responses (Table 4), PrEP awareness was 10.2%, and pleasure-related beliefs were heterogeneous: condoms reduce women’s pleasure (31.0%), condoms reduce men’s pleasure (43.4%), and condoms make sexual activity more playful (30.7%). The self-efficacy proxy (“used condom to prevent HIV/STIs” at the last reported sexual encounter) was 13.0%. At the socio-structural level, endorsement of the injunctive norm “condoms should be used even with a stable partner” reached 50.6%, perceived cost barriers were reported by 21.5% and access to sexual health services was 49.9%. Social support, as measured by the composite index, was distributed as 57.3%, 31.7%, and 11.0% across the 0/1/2 categories, respectively (Table 4).

This classification aligns with Supplementary Table S2.1, distinguishing descriptive versus injunctive normative constructs. Descriptive norms—treated as population-level contextual indicators rather than explanatory variables—were associated with consistently low condom use and higher prevalence of family communication about sex (Table S9a). Injunctive norms, conversely, were theory-driven explanatory factors, with pro-condom expectations and pleasure-related beliefs (Table S9b) directly associated with behavioral outcomes. Those endorsing the pro-condom injunctive norm demonstrated higher prevalence of both consistent condom use and HIV/STI testing (Table S9c). Together, these descriptive patterns underscore how normative contexts (opportunity in COM-B and social influences in TDF) intersect with individual knowledge, beliefs, and capabilities to shape prevention behaviors. Overall, the descriptive findings are consistent with COM-B, where reflective motivation (injunctive norms, self-efficacy, preventive beliefs), psychological capability (HIV knowledge, PrEP awareness), and social/physical opportunity (access, support, cost) jointly shape condom use and HIV/STI testing. Based on these patterns, we subsequently assessed the associations between individual and socio-structural determinants and condom use and HIV/STI testing in survey-weighted bivariate models (Supplementary Table S13).

### Bivariate associations

Survey-weighted bivariate models (Supplementary Table S13) showed that several individual and socio-structural determinants were associated with both outcomes. HIV knowledge was not significantly related to always using condoms (OR = 1.06, 95% CI: 0.99–1.13; *p* = .078) but was positively associated with HIV/STI testing (OR = 1.18, 1.13–1.23; *p* < .001). PrEP awareness was strongly related to both condom use (OR = 1.53, 1.24–1.89; *p* < .001) and testing (OR = 2.14, 1.81–2.52; *p* < .001). Beliefs that condoms reduce women’s or men’s pleasure were inversely associated with consistent condom use (OR = 0.72, 0.61–0.84; *p* < .001 and OR = 0.78, 0.67–0.91; *p* = .002, respectively) but not with testing. In contrast, the perception that condoms make sexual activity more playful was positively associated with both outcomes (condom use: OR = 1.30, 1.09–1.55; *p* = .003; testing: OR = 1.25, 1.10–1.42; *p* < .001). Self-efficacy (reporting condom use to prevent HIV/STIs) was not linked to consistent condom use (OR = 1.08, 0.84–1.39; *p* = .553) but was associated with being more likely to undergo testing (OR = 1.66, 1.33–2.07; *p* < .001).

Among socio-structural determinants, injunctive norms were strongly associated with both condom use (OR = 2.61, 2.18–3.12; *p* < .001) and testing (OR = 1.36, 1.17–1.58; *p* < .001). Perceived cost barriers reduced the likelihood of condom use (OR = 0.79, 0.64–0.97; *p* = .028) but were unrelated to testing. Access to services was negatively associated with consistent condom use (OR = 0.83, 0.70–0.97; *p* = .018) yet strongly associated with testing (OR = 2.14, 1.91–2.41; *p* < .001). Finally, social support was positively associated with both outcomes (condom use: OR = 2.39, 2.07–2.75; *p* < .001; testing: OR = 1.55, 1.38–1.73; *p* < .001). Supplementary analyses confirmed these patterns. Sex-stratified models (Supplementary Tables S10a–b) showed overlapping confidence intervals across men and women, with injunctive norms and social support remaining the strongest associated factors. Post-hoc checks of concordance between condom-use indicators (P73 vs. P89 and P119; Supplementary Table S11) showed strong agreement, and when P89 and P119 were analyzed as outcomes (Supplementary Table S12), associations mirrored those for consistent condom use, with pro-condom norms and social support linked to greater likelihood of condom use and cost barriers to lower likelihood.

### Multivariable, block-wise models

#### Consistent condom use (primary model, weighted)

In the weighted model for “always uses condoms,” the injunctive norm favorable to condom use was positively associated with the outcome (OR = 1.89; 1.46–2.43; *p* < .001). Women were less likely to use condoms than men (OR = 0.61; 0.46–0.81; *p* < .001). Associations for HIV knowledge, PrEP awareness, self-efficacy (used condom to prevent HIV/STIs), and access to services were not statistically significant after adjustment (Table 5).

**Table 5:** Adjusted odds ratios (aOR) from the multivariable model for consistent condom use (“always” vs. other), ENSSEX 2022–2023

	Determinant	OR (95% CI)
Individual determinants	HIV knowledge (per point)	0.99 (0.86–1.13)
	PrEP awareness	1.11 (0.81–1.51)
	Self-efficacy: used condom to prevent HIV/STIs	1.06 (0.80–1.41)
Socio-structural determinants	Access to services	1.08 (0.81–1.44)
	Injunctive norm (agree/strongly agree)	1.89 (1.46–2.43)
	Female (vs. male)	0.61 (0.46–0.81)

Notes: (1) Survey-weighted adjusted odds ratios (aORs) from logistic regression models that account for stratification, clustering, and sampling weights. (2) Outcome: consistent condom use in the last 12 months (“always” vs. other). (3) The model includes HIV knowledge (per-point score), PrEP awareness, self-efficacy (used condoms to prevent HIV/STIs), the injunctive norm about condom use, access to sexual health services, and sociodemographic covariates (sex, age, education, marital status, and sexual orientation). (4) Don’t-know and non-response categories were coded as missing (listwise deletion). (5) Results are presented as adjusted odd ratios (ORs) with 95% confidence intervals; estimates are considered statistically significant when 1.0 is not included in the interval

**Fig. 1:**
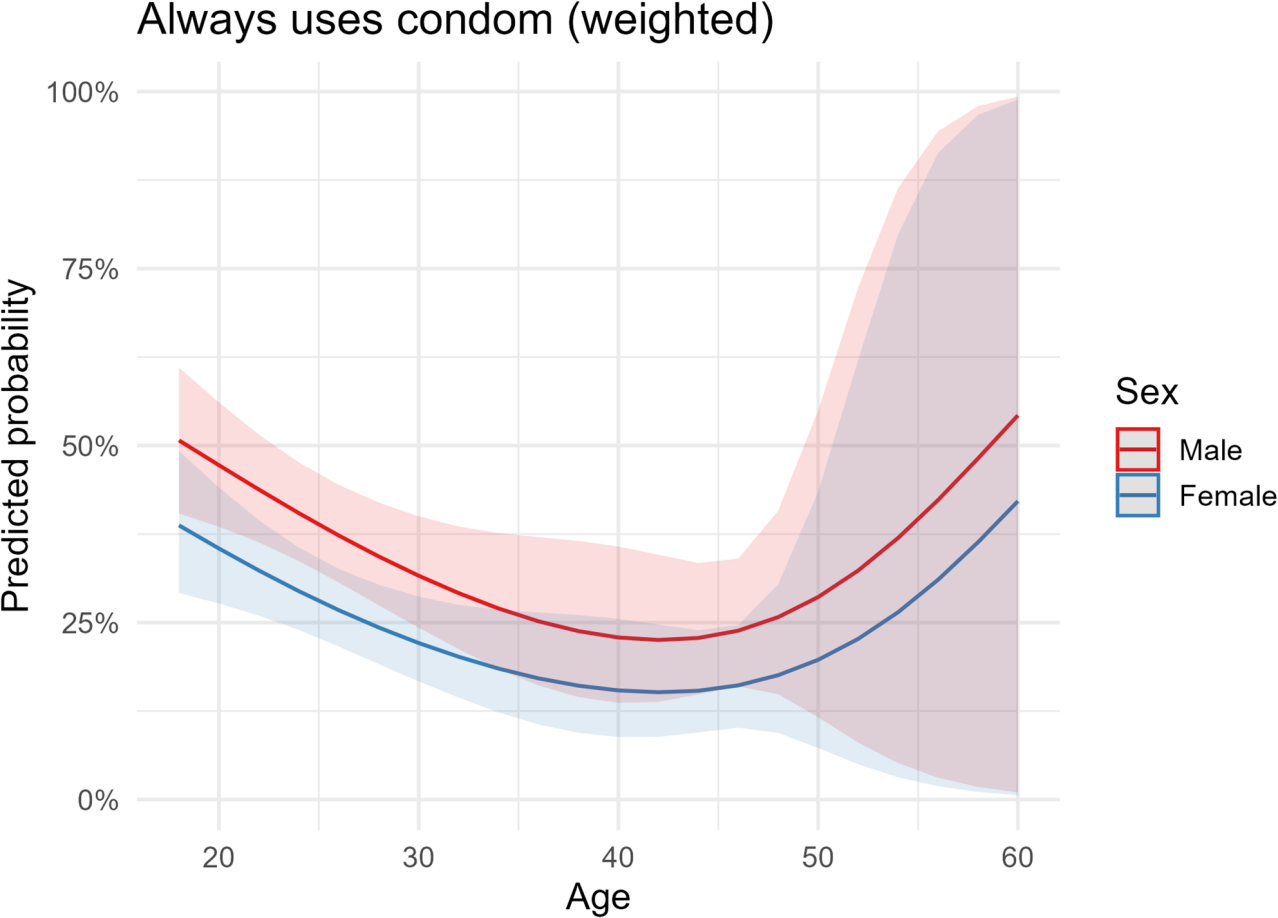
Survey-weighted predicted probability of “always” using condoms (P73) as a function of age using a natural cubic spline (df = 3), stratified by sex

Note: Survey-weighted predicted probability of “always” using condoms (P73) as a function of age using a natural cubic splines (df = 3), stratified by sex. Shaded bands indicate 95% CIs. Predicted probabilities adjusted for sociodemographic covariates and determinants as specified in Table 5.

Predicted probabilities decreased from late teens to the late thirties and then rose slightly at older ages; confidence intervals widened after age ~ 50, indicating greater uncertainty at older ages. Predicted probabilities were consistently higher among men across ages (Fig. 1).

#### HIV/STI testing (secondary model, weighted)

In the design-weighted logistic model with age modeled as a natural cubic spline (3 d.f.), PrEP awareness and access to sexual health services were among the strongest correlates of recent testing. Individuals aware of PrEP were 67% more likely to have tested (OR = 1.67; 95% CI 1.39–1.99; *p* < .001), and those who had consulted sexual health services, more than twice as likely (OR = 2.07; 1.80–2.38; *p* < .001). Participants identifying as gay/lesbian were also more likely to report testing than heterosexual participants (OR = 1.88; 1.42–2.50; *p* < .001). HIV knowledge, sex, and education were not statistically significant after adjustment (Table 6).

**Table 6:** Adjusted odds ratios (aOR) from the multivariable model for HIV/STI testing in the past 12 months (≥1 test), ENSSEX 2022–2023

	Determinantasd	OR (95% CI)
Individual determinants	HIV knowledge (per point)	1.03 (0.98–1.09)
	PrEP awareness	1.67 (1.39–1.99)
Socio-structural determinants	Access to services	2.07 (1.80–2.38)
Sociodemographic covariates	Education: 9–12 years (vs. ≤8)	0.91 (0.75–1.11)
	Education: ≥13 years (vs. ≤8)	0.98 (0.79–1.23)
	Female (vs. male)	1.10 (0.93–1.30)
	Gay/Lesbian (vs. heterosexual)	1.88 (1.42–2.50)

Notes. 1) Survey-weighted adjusted odds ratios (aORs) are estimated from logistic regression models that account for stratification, clustering, and sampling weights. 2) Outcome: HIV/STI testing in the past 12 months (≥1 test). 3) The model included HIV knowledge (per point), PrEP awareness, access to sexual health services, and sociodemographic covariates (sex, age, education, and sexual orientation). 4) “Don’t-know” and non-response categories were coded as missing (listwise deletion). 5) Results are presented as aORs with 95% confidence intervals; estimates are considered statistically significant when 1.0 is not included in the interval.

**Fig. 2:**
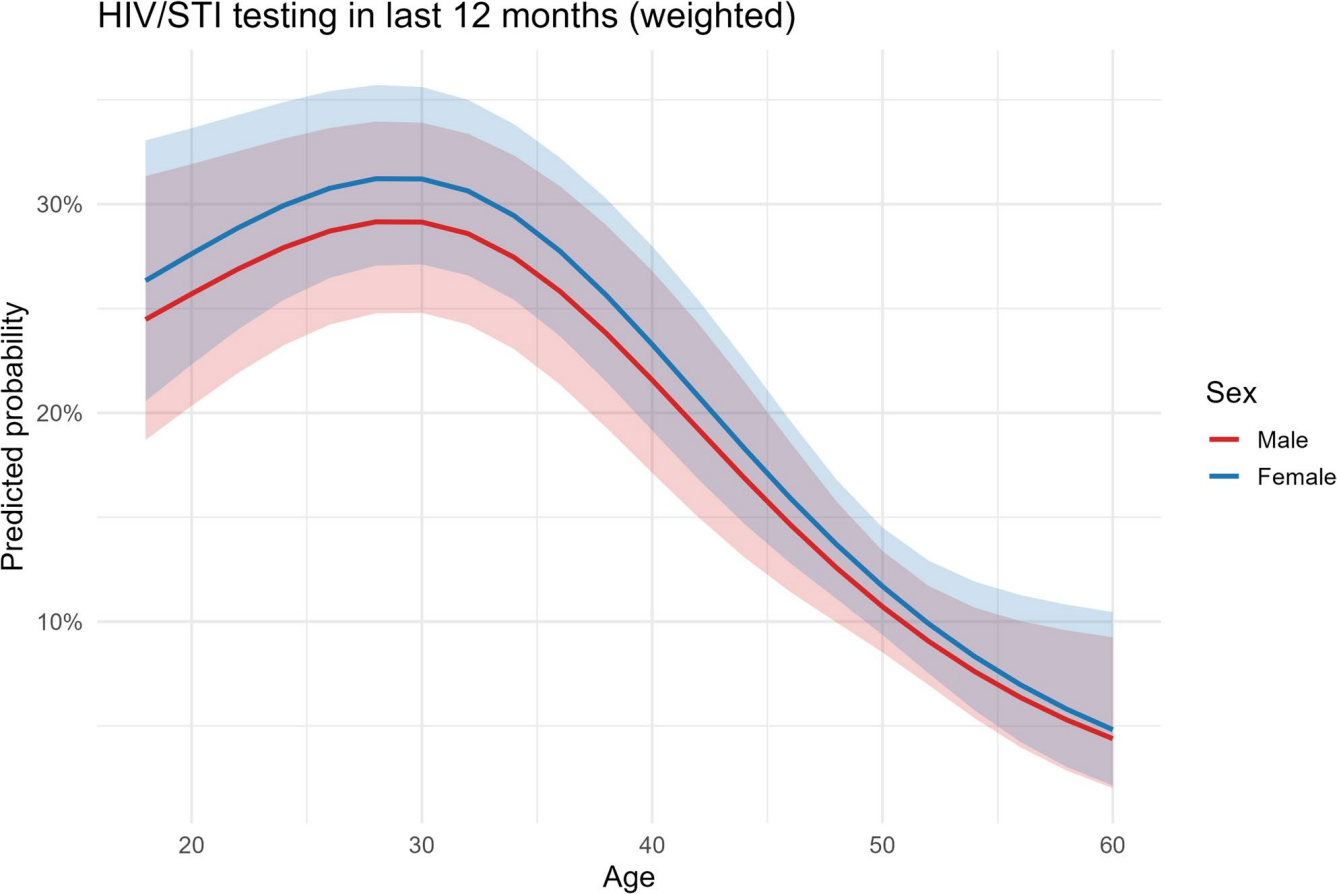
Survey-weighted predicted probability of ≥ 1 HIV/STI test in the past 12 months (P208) as a function of age (spline), stratified by sex

Note: Survey-weighted predicted probability of ≥ 1 HIV/STI test in the past 12 months (P208) as a function of age using natural cubic splines (df = 3), stratified by sex. Shaded bands show 95% CIs. Predicted probabilities adjusted for sociodemographic covariates and determinants as specified in Table 6.

Age displayed a steadily decreasing relationship with testing probability from early adulthood to older ages (Fig. 2). Model discrimination was acceptable in the corresponding unweighted diagnostic model (AUC ≈ 0.67); model calibration improved when the spline for age was used (Hosmer–Lemeshow *p* improved from < 0.01 without spline to ≈ 0.56 with spline), providing a rationale for its inclusion in the model. For transparency and reproducibility, the age-specific predicted probabilities corresponding to Figs. 1 and 2 are reported in the supplementary tables. Supplementary Table S5a presents weighted marginal-effect estimates with 95% confidence intervals for HIV/STI testing at ages 18, 20, 25, 30, 35, 40, 45, 50, 55, and 60, and Supplementary Table S5b provides the corresponding estimates for consistent condom use at the same ages, stratified by sex.

### Summary of findings

Taken together, these findings show that both individual and socio-structural determinants shape prevention behaviors, albeit in distinct ways. Reflective motivation (injunctive norms, pleasure-related beliefs, self-efficacy) was most consistently associated with consistent condom use, whereas physical opportunity (access to sexual health services) and specific psychological capability (PrEP awareness) were the strongest correlates of HIV/STI testing. Gender differences appeared mainly in condom use, with women being less likely than men to report consistent use, while testing patterns were better explained by access and knowledge-related factors. Overall, the results suggest that normative contexts (injunctive norms, social support) and individual beliefs act in concert to influence prevention behaviors, providing a coherent basis for theory-informed, multi-component intervention strategies.

## Discussion

### Main Findings

This nationally representative, theory-informed analysis of the Chilean National Health, Sexuality and Gender Survey (ENSSEX 2022–2023) identified distinct patterns of association underlying HIV/STI preventive behaviors. By applying the COM-B model and the Theoretical Domains Framework (TDF) to secondary survey data, we were able to differentiate how individual- and socio-structural determinants operate in real-world prevention contexts. Here, “distinct patterns” refers to well-differentiated constellations of behavioral determinants mapped to COM-B and TDF domains, rather than to causal or temporal pathways. Reflective motivation—captured through injunctive norms, pleasure-related beliefs, and self-efficacy—was most strongly associated with consistent condom use, whereas physical opportunity (access to sexual health services) and specific aspects of psychological capability (awareness of PrEP) emerged as the factors most strongly associated with HIV/STI testing. Women were less likely than men to report consistent condom use, yet sex differences were not evident for HIV/STI testing.

Beyond statistical significance, the observed sex difference in consistent condom use warrants further interpretation. The lower likelihood of condom use among women may reflect contextual and relationship-level barriers rather than lower levels of individual knowledge or motivation. Prior research suggests that women’s capacity to negotiate condom use is often shaped by gendered power dynamics, partner resistance, and concerns about relationship stability, which can constrain behavioral opportunity even when motivation is present. Within the COM-B framework, this pattern is more consistent with reduced social opportunity—particularly normative and relational factors—than with limited psychological capability.

Taken together, these findings indicate that normative and structural contexts are more strongly associated with prevention behaviors than knowledge alone.

### Comparison with the literature

Our results align with prior meta-analyses showing that knowledge-based interventions alone are insufficient to change sexual behaviors when broader motivational and structural determinants remain unaddressed [14, 15]. The explanatory role of injunctive norms is consistent with classic work distinguishing descriptive from prescriptive norms [24], and with evidence that perceived social approval can exert a stronger influence on condom use than knowledge or attitudes [18]. Similarly, the central role of service access and PrEP awareness parallels global findings that expanded biomedical availability and awareness drives HIV/STI testing [19].

Our study also underscores the well-documented intention–behavior gap: although many participants reported preventive intentions, consistent condom use remained low, echoing the findings of Albarracín et al. (2001) [14]. This pattern highlights the importance of supportive contexts—including access to services and enabling norms—in helping individuals translate motivation into sustained preventive behavior.

### Theoretical contributions (COM-B/TDF)

Mapping ENSSEX items to COM-B and TDF provided clear insight into how different determinants relate to behavior. Condom use was predominantly shaped by reflective motivation—such as injunctive norms, beliefs about consequences, and goal-directed self-efficacy—whereas HIV/STI testing was more strongly driven by opportunity (especially access to services) and specific forms of psychological capability (awareness of PrEP). Notably, general HIV knowledge was no longer associated with either outcome once structural and motivational determinants were taken into account, emphasizing that capability alone is insufficient unless supported by opportunity and motivation to produce changes in behavior.

These associations reflect how COM-B and TDF constructs were operationalized through specific ENSSEX survey items, linking psychological capability (e.g., HIV knowledge and PrEP awareness), reflective motivation (e.g., pleasure-related beliefs and self-efficacy), and social and physical opportunity (e.g., injunctive norms and access to services) to observed prevention behaviors. Taken together, the pattern suggests a gradient across COM-B components, in which socio-structural determinants carry greater weight than knowledge and self-efficacy plays a central role in condom use, with sex differences emerging mainly for self-efficacy rather than for normative beliefs.

These findings are also consistent with broader behavioral science frameworks. A recent scoping review of the Fogg Behavior Model (FBM) in health interventions reported that effective behavior change strategies typically depend on the alignment of motivation, ability, and prompts across contexts ranging from sexual health to chronic disease management [25]. The parallels are notable: while COM-B/TDF helped classify determinants of condom use and HIV/STI testing in Chile, the FBM points to similar mechanisms in which motivation and ability must converge with contextual triggers for behavior change to occur. Integrating these perspectives may strengthen the theoretical foundation of future interventions, combining the detailed diagnostic capacity of COM-B/TDF with the more design-oriented focus of the FBM.

### Strengths and limitations

Strengths include the use of a large, nationally representative dataset, the application of survey design weights, and the systematic mapping of variables to COM-B/TDF. To our knowledge, this is the first national study in Latin America to apply behavioral science frameworks to HIV/STI prevention outcomes. Limitations include the cross-sectional design, which implies that all associations are interpreted as correlational rather than causal; COM-B and TDF are therefore used as interpretative frameworks to structure patterns of association, not to infer causal pathways. Additional limitations include reliance on self-reported data, which are subject to recall and social desirability biases, and a narrow operationalization of social norms, approximated via injunctive proxies. The self-efficacy measure was based on a single item, so findings should be interpreted with caution.

A further limitation is that this study relies on secondary analysis of cross-sectional, observational data, which restricts causal inference. Following Holland’s reflections on causal inference (1986) [26], fixed attributes such as sex, age, or sexual orientation cannot be considered causal factors in an experimental sense, but rather markers of social stratification whose associations reflect underlying mechanisms (e.g., discrimination, access barriers, normative expectations). Acknowledging this distinction strengthens our analytic focus on modifiable determinants—such as access to services, injunctive norms, and social support—identified through COM-B and TDF. These are the factors most directly amenable to intervention in HIV/STI prevention.

### Public health and policy implications

Our findings have direct implications for prevention in Chile and similar contexts. Interventions focused solely on knowledge transfer are unlikely to yield substantial change. Instead, strategies should reinforce normative support for condom use, reduce structural barriers (e.g., affordability, access to services), and increase awareness of biomedical tools like PrEP. The role of social support highlights the need to engage families, peers, and partners in prevention efforts.

Importantly, these findings connect with evidence from digital health. Our recent overview of digital behavior change interventions for HIV/STI prevention [20] showed that the most effective digital strategies integrated motivational content with opportunities for access and normative reinforcement. Taken together, this suggests that digital platforms in Chile could be leveraged to reinforce pro-condom norms, normalize HIV/STI testing, and provide navigation support for PrEP and sexual health services, beyond traditional information campaigns.

In the Chilean context, youth-led or peer-mediated campaigns could normalize condom use and routine HIV/STI testing, while stigma-free health services may reduce barriers for men and other underserved groups [4, 27]. In line with recent meta-analyses highlighting the effectiveness of multicomponent strategies [19], national policies should integrate access, normative reinforcement, and motivational components rather than focusing on single determinants.

### Future directions

Future studies should employ longitudinal and experimental designs to test causal pathways and intervention strategies. Adolescents and LGBTQ+ populations, whose prevention needs and determinants differ, warrant special attention. Expanding measures of peer, community, and online norms would strengthen explanatory power. The application of the PROGRESS-Plus framework could further elucidate equity dimensions, ensuring that prevention strategies address intersecting disparities (for example, by sex, socioeconomic status, education, sexual orientation, and place of residence) [28–30].

Importantly, this study represents the first population-level application of the COM-B model and TDF to HIV/STI prevention in Latin America. Building on this precedent, future research in the region should further integrate behavioral science frameworks into population surveys, enabling more precise mapping of barriers and enablers across diverse cultural and structural contexts. Finally, embedding COM-B and TDF into the design and evaluation of digital and community-based interventions could strengthen cultural tailoring and scalability, maximizing public health impact.

## Conclusion

This study provides the first nationally representative evidence from Latin America to apply behavioral science frameworks to HIV/STI prevention. Our findings show that prevention behaviors are more strongly associated with normative and structural determinants than with knowledge alone. Consistent condom use was most strongly associated with reflective motivation—injunctive norms, beliefs about sexual pleasure, and self-efficacy—whereas HIV/STI testing was most strongly associated with physical opportunity (access to services) and specific psychological capability (PrEP awareness). Women reported lower odds of consistent condom use than men, while testing patterns were largely explained by access and awareness rather than sex.

These results reinforce global evidence that knowledge, while necessary, is less strongly associated with sustained prevention than opportunity and motivation. They also demonstrate the value of embedding behavioral frameworks such as the COM-B model and the TDF into epidemiological analyses, providing conceptual clarity and actionable insights for policy. By aligning with complementary models such as the Fogg Behavior Model, this study highlights an approach toward integrated, multicomponent prevention strategies. National and international programs should prioritize interventions that strengthen normative support, expand structural access, and leverage digital and community-based tools to help bridge the gap between intention and action.

## Supplementary Information

Supplementary Material 1.

## Data Availability

All data analyzed in this study are publicly available through the ENSSEX 2022–2023 national survey (https:/datos.gob.cl/dataset/encuesta-nacional-de-salud-sexualidad-y-genero-enssex-2022-2023/resource/ed81f50c-1c7d-43d9-9083-dfc161e0cd66). No additional data were generated.

## Abbreviations

AACTT: Action, Actor, Context, Target, Time
BCT: behavior change technique
BCTTv1: Behavior Change Technique Taxonomy version 1
BCW: Behavior Change Wheel
COM-B: Capability, Opportunity, Motivation - Behavior
ENSSEX: Chilean National Health, Sexuality, and Gender Survey
HIV: Human Immunodeficiency Virus
LGBTQ+: Lesbian, Gay, Bisexual, Transgender, Queer, and other sexual orientations and gender identities
PrEP: Pre-exposure Prophylaxis
STI: sexually transmitted infection
TDF: Theoretical Domains Framework
