# Supplementary material for: Socio-structural and individual determinants of HIV/STI prevention behaviors in Chile: a national cross-sectional analysis
Source: BMC Public Health. 2026 Mar 12;26:1289. doi: 10.1186/s12889-026-26934-z (PMC13097754; doi:10.1186/s12889-026-26934-z)
Supplement: Supplementary file 1 — Supplementary Material 1. [file 12889_2026_26934_MOESM1_ESM.docx]

**Socio-structural and individual determinants of HIV/STI prevention behaviors in Chile: a national cross-sectional analysis -Multimedia Appendices**

Contents

[Appendix 1. 305 items included in the national survey (ENSSEX 2022-2023). -including sub items (405) 1](#_Toc222595848)

[Appendix 2 – Structured Coding Matrix of Selected ENSEXX Survey Items: Variable Labels, Theoretical Mapping, and Justification 16](#_Toc222595849)

[Appendix Table S2.1–Classification of ENSSEX items into descriptive and injunctive (prescriptive) norms 25](#_Toc222595850)

[Appendix 3 –. Final Coding Matrix: Mapping of ENSEXX Survey Items to TDF, and COM-B Frameworks 26](#_Toc222595851)

[Appendix 4 – Intercoder reliability calculation report 31](#_Toc222595852)

[Appendix S5. Model diagnostics (AUC, Hosmer–Lemeshow, spline plots, sensitivity to missingness). 32](#_Toc222595853)

[Appendix Table S5a and S5b 33](#_Toc222595854)

[Appendix S6. Exploratory sex interactions (SAGER compliance) 41](#_Toc222595855)

[Appendix Supplementary Table S7. Consistency checks with P89 and P119. 42](#_Toc222595856)

[Appendix Supplementary Table S8. Full Distributions 43](#_Toc222595857)

[Appendix Supplementary Table S9a-c. Full response distributions for descriptive and injunctive norms related to HIV/STI prevention behaviors (ENSSEX 2022–2023) 45](#_Toc222595858)

[Appendix Supplementary Table S10 a-b. Full Distributions 46](#_Toc222595859)

[Appendix Supplementary Table S11 a-b. Full Distributions 47](#_Toc222595860)

[Appendix Supplementary Table S12 48](#_Toc222595861)

[Appendix Supplementary Table S13. Survey-weighted crude odds ratios (bivariate associations) for consistent condom use and HIV/STI testing (ENSSEX 2022–2023) 48](#_Toc222595862)

# Appendix 1. 305 items included in the national survey (ENSSEX 2022-2023). -including sub items (405)

| **N°** | **Variable** | **Questionnaire item (English translation)** |
| --- | --- | --- |
| 1 | sexo [Sex] | - |
| 2 | edad [Age] | - |
| 3 | p1 | ¿Cuál es su sexo asignado al nacer? [What is your sex assigned at birth?] |
| 4 | p2 | ¿Es usted una persona intersex? [Are you an intersex person?] |
| 5 | p3 | ¿Cuál es el género con el que Usted se identifica? [What gender do you identify with?] |
| 6 | p3_otro | - |
| 7 | p4 | ¿Qué edad tiene? [How old are you?] |
| 8 | p5 | ¿Cuál es su nivel educacional más alto alcanzado o su nivel educacional actual? [What is your highest level of education attained or your current educational level?] |
| 9 | p6 | En ese nivel educacional, ¿cuál fue el último curso que aprobó (para los que no... [At that educational level, what was the last grade you passed (for those who did not...] |
| 10 | p7 | ¿Cuál es su estado conyugal o civil actual? [What is your current marital or civil status?] |
| 11 | p8 | ¿Cómo calificaría su calidad de vida? [How would you rate your quality of life?] |
| 12 | i_1_p9 | (Con la privacidad que tiene donde vive) Ahora le preguntaré acerca de cómo se siente. [With the privacy you have where you live, I will now ask you how you feel.] |
| 13 | i_2_p9 | (Con su bienestar mental o emocional) Ahora le preguntaré acerca de cómo se siente. [With your mental or emotional well-being, I will now ask you how you feel.] |
| 14 | i_3_p9 | (Con su vida amorosa) Ahora le preguntaré acerca de cómo se siente. [With your love life, I will now ask you how you feel.] |
| 15 | i_4_p9 | (Con la cantidad de diversión que tiene en su vida) Ahora le preguntaré acerca de cómo se siente. [With the amount of fun you have in your life, I will now ask you how you feel.] |
| 16 | i_5_p9 | (Con su vida familiar) Ahora le preguntaré acerca de cómo se siente Usted en distintos aspectos. [With your family life, I will now ask you how you feel in different aspects.] |
| 17 | i_6_p9 | (Con su vida sexual) Ahora le preguntaré acerca de cómo se siente Usted en distintos aspectos. [With your sex life, I will now ask you how you feel in different aspects.] |
| 18 | p10 | En general Usted diría que su salud es... [In general, you would say that your health is...] |
| 19 | i_1_p11 | (Poco interés o placer en hacer cosas) Durante las dos últimas semanas, ¿con qué frecuencia ha sentido molestias, debido a cualquiera de los siguientes problemas? [Little interest or pleasure in doing things: In the last two weeks, how often have you felt distress due to any of the following problems?] |
| 20 | i_2_p11 | (Se ha sentido decaído(a), deprimido(a) o sin esperanzas) Durante las dos últimas semanas, ¿con qué frecuencia ha sentido molestias, debido a cualquiera de los siguientes problemas? [Feeling down, depressed, or hopeless: In the last two weeks, how often have you felt distress due to any of the following problems?] |
| 21 | i_3_p11 | (Se ha sentido nervioso(a), ansioso(a) o con los nervios de punta) Durante las dos últimas semanas, ¿con qué frecuencia ha sentido molestias, debido a cualquiera de los siguientes problemas? [Feeling nervous, anxious, or on edge: In the last two weeks, how often have you felt distress due to any of the following problems?] |
| 22 | i_4_p11 | (No ha sido capaz de parar o controlar su preocupación) Durante las dos últimas semanas, ¿con qué frecuencia ha sentido molestias, debido a cualquiera de los siguientes problemas? [Not being able to stop or control worrying: In the last two weeks, how often have you felt distress due to any of the following problems?] |
| 23 | i_1_p12 | ¿Alguna vez en su vida ha tenido la experiencia A? Pensar seriamente en suicidarse. [Have you ever in your life had experience A? Seriously thinking about suicide.] |
| 24 | i_1_p13 | ¿Alguna vez en su vida ha tenido la experiencia B? Hacer un plan para suicidarse. ¿Ha tenido la experiencia B en los últimos 12 meses? [Have you ever in your life had experience B? Making a plan to commit suicide. Have you had experience B in the last 12 months?] |
| 25 | i_2_p12 | (Experiencia B.) Ahora vea la tarjeta 6. Están anotadas dos experiencias designadas A y B. [Experience B. Now look at card 6. Two designated experiences A and B are written.] |
| 26 | i_2_p13 | (Experiencia B.) ¿Ha tenido la experiencia... en los últimos 12 meses? [Experience B. Have you had the experience... in the last 12 months?] |
| 27 | i_1_p16 | (Ver, sin usar anteojos ópticos o lentes?) En los últimos 30 días y sin tener en cuenta el uso de lentes. [Seeing, without using glasses or lenses? In the last 30 days and disregarding the use of glasses.] |
| 28 | i_2_p16 | (Oír, sin usar audífono o dispositivo de ayuda para oír?) En los últimos 30 días. [Hearing, without using a hearing aid or hearing device? In the last 30 days.] |
| 29 | i_3_p16 | (Caminar o subir peldaños?) En los últimos 30 días y sin tener en cuenta ningún dispositivo de ayuda. [Walking or climbing stairs? In the last 30 days and without considering any assistive device.] |
| 30 | i_4_p16 | ¿Recordar cosas o concentrarse? [Remembering things or concentrating?] |
| 31 | i_5_p16 | (Asearse o vestirse?) En los últimos 30 días y sin tener en cuenta ningún tipo de ayuda [Grooming or dressing? In the last 30 days and without any kind of help] |
| 32 | i_6_p16 | (Dormir?) En los últimos 30 días y sin tener en cuenta ningún tipo de ayuda [Sleeping? In the last 30 days and without any kind of help] |
| 33 | i_7_p16 | (Hacer las tareas de la casa como barrer, cocinar, hacer arreglos o sacar la basura) [Doing household chores such as sweeping, cooking, doing repairs or taking out the garbage] |
| 34 | i_8_p16 | (Participar en fiestas, eventos religiosos, reuniones vecinales u otras actividades) [Participating in parties, religious events, neighborhood meetings, or other activities] |
| 35 | i_9_p16 | ¿Llevarse bien con la gente cercana a usted, incluyendo su familia, amigos y amigas? [Getting along with people close to you, including family, friends, and others?] |
| 36 | i_1_p17 | Debido a su salud, ¿qué grado de dificultad le ha generado en su vida… (Sentirse triste, deprimido(a), preocupado(a) o ansioso(a)?) [Due to your health, how much difficulty has it caused you in your life… (Feeling sad, depressed, worried, or anxious?)] |
| 37 | i_2_p17 | Debido a su salud, ¿qué grado de dificultad le ha generado en su vida… ¿Sentir algún malestar o dolor físico, por ejemplo, dolor de espalda, dolor de estómago o dolor de cabeza? [Due to your health, how much difficulty has it caused you in your life… Feeling some discomfort or physical pain, such as back pain, stomach pain or headache?] |
| 38 | i_1_p18a1 | A continuación, le voy a preguntar por algunas enfermedades que Usted tiene o ha tenido (Migrañas o dolores de cabeza frecuentes) ¿Alguna vez un doctor o médico le ha dicho que tiene o que padece de...? [I will now ask you about some illnesses that you have or have had (Migraines or frequent headaches). Has a doctor or physician ever told you that you have or suffer from...?] |
| 39 | i_1_p18b1 | (Migrañas o dolores de cabeza frecuentes) ¿A qué edad se la diagnosticaron... [Migraines or frequent headaches: At what age were you diagnosed...?] |
| 40 | i_1_p18c1 | (Migrañas o dolores de cabeza frecuentes) ¿Alguna vez ha sido tratado por esto... [Migraines or frequent headaches: Have you ever been treated for this...?] |
| 41 | i_1_p18d1 | (Migrañas o dolores de cabeza frecuentes) ¿Alguna vez ha sido operado por esto... [Migraines or frequent headaches: Have you ever had surgery for this...?] |
| 42 | i_1_p18e1 | (Migrañas o dolores de cabeza frecuentes) ¿Ha estado tomando algún medicamento o [Migraines or frequent headaches: Have you been taking any medication or] |
| 43 | i_2_p18a1 | (Trastorno musculo esquelético (dolor de espalda crónico, pierna, brazos, hombro...¿Alguna vez un doctor o médico le ha dicho que tiene o que padece de...? [Musculoskeletal disorder (chronic pain in the back, legs, arms, shoulder)... Has a doctor ever told you that you have or suffer from...?] |
| 44 | i_2_p18b1 | (Trastorno musculo esquelético) ¿A qué edad se la diagnosticaron? [Musculoskeletal disorder: At what age were you diagnosed?] |
| 45 | i_2_p18c1 | (Trastorno musculo esquelético) ¿Alguna vez ha sido tratado por esto...? [Musculoskeletal disorder: Have you ever been treated for this...?] |
| 46 | i_2_p18d1 | (Trastorno musculo esquelético) ¿Alguna vez ha sido operado por esto...? [Musculoskeletal disorder: Have you ever had surgery for this...?] |
| 47 | i_2_p18e1 | (Trastorno musculo esquelético) ¿Ha estado tomando algún medicamento o [Musculoskeletal disorder: Have you been taking any medication or] |
| 48 | i_1_p18a2 | (Depresión) ¿Alguna vez un doctor o médico le ha dicho que tiene o que padece de [Depression: Has a doctor or physician ever told you that you have or suffer from] |
| 49 | i_1_p18b2 | (Depresión) ¿A qué edad se la diagnosticaron [Depression: At what age were you diagnosed?] |
| 50 | i_1_p18c2 | (Depresión) ¿Alguna vez ha sido tratado por esto... [Depression: Have you ever been treated for this?] |
| 51 | i_1_p18e2 | (Depresión) ¿Ha estado tomando algún medicamento o haciendo algún tratamiento [Depression: Have you been taking any medication or undergoing any treatment?] |
| 52 | i_1_p18a3 | (Diabetes) ¿Alguna vez un doctor o médico le ha dicho que tiene o que padece de. [Diabetes: Has a doctor ever told you that you have or suffer from it?] |
| 53 | i_1_p18b3 | (Diabetes) ¿A qué edad se la diagnosticaron... [Diabetes: At what age were you diagnosed?] |
| 54 | i_1_p18c3 | (Diabetes) ¿Alguna vez ha sido tratado [Diabetes: Have you ever been treated for it?] |
| 55 | i_1_p18d3 | (Diabetes) ¿Alguna vez ha sido operado por [Diabetes: Have you ever had surgery for it?] |
| 56 | i_1_p18e3 | (Diabetes) ¿Ha estado tomando algún medicamento o haciendo algún tratamiento pa [Diabetes: Have you been taking any medication or undergoing any treatment for it?] |
| 57 | i_2_p18a3 | (Bocio, enfermedad a la tiroides hipertiroidismo o hipotiroidismo) ¿Alguna vez u [Goiter, thyroid disease: hyperthyroidism or hypothyroidism – Has a doctor ever told you that you have or suffer from it?] |
| 58 | i_2_p18b3 | (Bocio, enfermedad a la tiroides hipertiroidismo o hipotiroidismo) ¿A qué edad s [Goiter, thyroid disease: At what age were you diagnosed?] |
| 59 | i_2_p18c3 | (Bocio, enfermedad a la tiroides hipertiroidismo o hipotiroidismo) ¿Alguna vez ha sido tratado [Goiter, thyroid disease: Have you ever been treated for it?] |
| 60 | i_2_p18d3 | (Bocio, enfermedad a la tiroides hipertiroidismo o hipotiroidismo) ¿Alguna vez ha sido operado [Goiter, thyroid disease: Have you ever had surgery for it?] |
| 61 | i_2_p18e3 | (Bocio, enfermedad a la tiroides hipertiroidismo o hipotiroidismo) ¿Ha estado to [Goiter, thyroid disease: Have you been taking any medication or undergoing any treatment for it?] |
| 62 | i_3_p18a3 | (Sobrepeso u obesidad) ¿Alguna vez un doctor o médico le ha dicho que tiene o qu [Overweight or obesity: Has a doctor ever told you that you have or suffer from it?] |
| 63 | i_3_p18b3 | (Sobrepeso u obesidad) ¿A qué edad se la diagnosticaron... @_@ITERNAME [Overweight or obesity: At what age were you diagnosed?] |
| 64 | i_3_p18c3 | (Sobrepeso u obesidad) ¿Alguna vez ha sido tratado por esto... [Overweight or obesity: Have you ever been treated for this?] |
| 65 | i_3_p18d3 | (Sobrepeso u obesidad) ¿Alguna vez ha sido operado [Overweight or obesity: Have you ever had surgery for it?] |
| 66 | i_3_p18e3 | (Sobrepeso u obesidad) ¿Ha estado tomando algún medicamento o haciendo algún tra [Overweight or obesity: Have you been taking any medication or undergoing any treatment for it?] |
| 67 | i_4_p18a3 | (Hipertensión) ¿Alguna vez un doctor o médico le ha dicho que tiene o que padece [Hypertension: Has a doctor ever told you that you have or suffer from it?] |
| 68 | i_4_p18b3 | (Hipertensión) ¿A qué edad se la diagnosticaron... [Hypertension: At what age were you diagnosed?] |
| 69 | i_4_p18c3 | (Hipertensión) ¿Alguna vez ha sido tratado por esto... [Hypertension: Have you ever been treated for this?] |
| 70 | i_4_p18d3 | (Hipertensión) ¿Alguna vez ha sido operado por esto... [Hypertension: Have you ever had surgery for this?] |
| 71 | i_4_p18e3 | (Hipertensión) ¿Ha estado tomando algún medicamento o haciendo algún tratamiento [Hypertension: Have you been taking any medication or undergoing any treatment?] |
| 72 | i_5_p18a3 | (Infertilidad) ¿Alguna vez un doctor o médico le ha dicho que tiene o que padece [Infertility: Has a doctor ever told you that you have this?] |
| 73 | i_5_p18b3 | (Infertilidad) ¿A qué edad se la diagnosticaron [Infertility: At what age were you diagnosed?] |
| 74 | i_5_p18c3 | (Infertilidad) ¿Alguna vez ha sido tratado por esto... @_@I [Infertility: Have you ever been treated for this?] |
| 75 | i_5_p18d3 | (Infertilidad) ¿Alguna vez ha sido operado por esto... [Infertility: Have you ever had surgery for this?] |
| 76 | i_5_p18e3 | (Infertilidad) ¿Ha estado tomando algún [Infertility: Have you been taking any medication for this?] |
| 77 | p19 | ¿Ha sido diagnosticado con COVID-19? [Have you been diagnosed with COVID-19?] |
| 78 | p20 | ¿Alguna vez se ha realizado una mamografía? [Have you ever had a mammogram?] |
| 79 | p20b | ¿Alguna vez se ha realizado un papanicolaou? [Have you ever had a Pap smear?] |
| 80 | p20c | ¿Alguna vez se ha realizado un control de antígeno prostático? [Have you ever had a prostate-specific antigen (PSA) test?] |
| 81 | p21a | ¿En los últimos tres años se ha realizado una mamografía? [In the past three years, have you had a mammogram?] |
| 82 | p21b | ¿En los últimos tres años se ha realizado un papanicolaou? [In the past three years, have you had a Pap smear?] |
| 83 | p21c | ¿En los últimos tres años se ha realizado un control de antígeno prostático? [In the past three years, have you had a PSA test?] |
| 84 | p22 | ¿Me podría decir cuánto pesa aproximadamente actualmente? [Could you tell me your approximate current weight?] |
| 85 | p23 | ¿Me podría decir cuánto mide aproximadamente actualmente? [Could you tell me your approximate current height?] |
| 86 | i_1_p24 | (Me gusta mi aspecto físico tal y como es) ¿Qué tan de acuerdo está con las sigu [I like the way my body looks just as it is – To what extent do you agree with the following] |
| 87 | i_2_p24 | (Me gusta el aspecto de mi cuerpo sin la ropa)¿Qué tan de acuerdo está con las sigu [I like how my body looks without clothes – To what extent do you agree with the following] |
| 88 | i_3_p24 | (Soy físicamente atractivo/a) ¿Qué tan de acuerdo está con las siguientes afirma [I am physically attractive – To what extent do you agree with the following statements] |
| 89 | i_1_p25 | (Cocaína) ¿Ha probado Ud. alguna de las siguientes sustancias alguna vez en su v [Cocaine: Have you ever tried any of the following substances in your life?] |
| 90 | i_1_p26 | (Cocaína) ¿Cuándo fue la última vez que consumió? [Cocaine: When was the last time you used it?] |
| 91 | i_2_p25 | (Marihuana) ¿Ha probado Ud. alguna de las siguientes sustancias alguna vez en su vida? [Marijuana: Have you ever tried any of the following substances in your life?] |
| 92 | i_2_p26 | (Marihuana) ¿Cuándo fue la última vez que consumió? [Marijuana: When was the last time you used it?] |
| 93 | i_3_p25 | (Drogas inyectables) ¿Ha probado Ud. alguna de las siguientes sustancias alguna vez en su vida? [Injectable drugs: Have you ever tried any of the following substances in your life?] |
| 94 | i_3_p26 | (Drogas inyectables) ¿Cuándo fue la última vez que consumió? [Injectable drugs: When was the last time you used it?] |
| 95 | i_4_p25 | (Tranquilizantes, ansiolíticos o antidepresivo) ¿Ha probado Ud. alguna de las siguientes sustancias alguna vez en su vida? [Tranquilizers, anxiolytics or antidepressants: Have you ever tried any of the following substances in your life?] |
| 96 | i_4_p26 | (Tranquilizantes, ansiolíticos o antidepresivo) ¿Cuándo fue la última vez que consumió? [Tranquilizers, anxiolytics or antidepressants: When was the last time you used it?] |
| 97 | i_5_p25 | (Alcohol) ¿Ha probado Ud. alguna de las siguientes sustancias alguna vez en su vida? [Alcohol: Have you ever tried any of the following substances in your life?] |
| 98 | i_5_p26 | ¿Cuándo fue la última vez que consumió? [When was the last time you used it?] |
| 99 | i_6_p25 | (Alucinógenos, hongos) ¿Ha probado Ud. alguna de las siguientes sustancias alguna vez en su vida? [Hallucinogens, mushrooms: Have you ever tried any of the following substances in your life?] |
| 100 | i_6_p26 | (Alucinógenos, hongos) ¿Cuándo fue la última vez que consumió? [Hallucinogens, mushrooms: When was the last time you used it?] |
| 101 | i_7_p25 | (Poppers) ¿Ha probado Ud. alguna de las siguientes sustancias alguna vez en su vida? [Poppers: Have you ever tried any of the following substances in your life?] |
| 102 | i_7_p26 | (Poppers) ¿Cuándo fue la última vez que consumió? [Poppers: When was the last time you used it?] |
| 103 | i_8_p25 | (Otra) ¿Ha probado Ud. alguna de las siguientes sustancias alguna vez en su vida? [Other: Have you ever tried any of the following substances in your life?] |
| 104 | i_8_p26 | (Otra) ¿Cuándo fue la última vez que consumió? [Other: When was the last time you used it?] |
| 105 | p25_otra1 | ¿Cuál? Drogas inyectables [Which one? Injectable drugs] |
| 106 | p25_otra2 | ¿Cuál? Otra [Which one? Other] |
| 107 | p27 | Si usted pudiera volver a la época en que todavía no tenía hijos/as y pudiera elegir exactamente el número de hijos/as que tendría en toda su vida ¿Cuántos serían? [If you could go back to the time before you had children and choose exactly how many children you would have in your life, how many would that be?] |
| 108 | i_1_p28 | Respecto a la sexualidad y el género… ¿Cuán de acuerdo está Ud. con...? Hay que tener un orgasmo para que una relación sexual sea verdaderamente satisfactoria. [Regarding sexuality and gender… To what extent do you agree with the statement: One must have an orgasm for a sexual relationship to be truly satisfying?] |
| 109 | i_2_p28 | Es necesario mantener relaciones sexuales regularmente para el bienestar personal. [It is necessary to have regular sexual activity for personal well-being.] |
| 110 | i_3_p28 | Los hombres por naturaleza tienen más necesidades sexuales que las mujeres. [Men naturally have more sexual needs than women.] |
| 111 | i_4_p28 | La homosexualidad es una forma de vivir la sexualidad tan válida como cualquier otra. [Homosexuality is a way of living sexuality just as valid as any other.] |
| 112 | i_5_p28 | Una mujer debe tener derecho a interrumpir un embarazo, si no desea ser madre o no desea serlo en ese momento o circunstancia. [A woman should have the right to terminate a pregnancy if she does not want to be a mother or does not wish to be one at that time or under those circumstances.] |
| 113 | i_6_p28 | La masturbación es una práctica aceptable teniendo pareja. [Masturbation is an acceptable practice when in a relationship.] |
| 114 | i_7_p28 | Solo se debe tener sexo cuando hay amor. [One should only have sex when there is love.] |
| 115 | p29 | ¿Usted conoce personalmente a personas lesbianas, gay, bisexual, queer, intersex, trans, etc.? [Do you personally know any lesbian, gay, bisexual, queer, intersex, or trans people?] |
| 116 | p30_o1 | ¿Y de dónde conoce a esa/a persona/s? [And where do you know this person/these people from?] |
| 117 | p31 | Si uno de sus hijos/as le dijera que es homosexual (gay), lesbiana, bisexual u otra, usted.... [If one of your children told you they were gay, lesbian, bisexual or other, you would…] |
| 118 | p32 | Si uno de sus hijos/as le dijera que es trans (transgénero masculino, transgénero femenino), usted…: (LEA ALTERNATIVAS) [If one of your children told you they were transgender (male-to-female or female-to-male), you would… (READ OPTIONS)] |
| 119 | i_1_p33 | (Usar preservativos o condón disminuye el placer de las mujeres.) [Using condoms reduces women's sexual pleasure.] |
| 120 | i_2_p33 | (Usar preservativos o condón disminuye el placer de los hombres.) [Using condoms reduces men's sexual pleasure.] |
| 121 | i_3_p33 | (Es necesario ocupar preservativo o condón incluso si se tiene pareja estable) [It is necessary to use condoms even in a stable relationship.] |
| 122 | i_4_p33 | (Usar preservativo o condón estimula el juego sexual.) [Using condoms stimulates sexual play.] |
| 123 | i_5_p33 | (Los preservativos o condones son demasiado caros para usarlos regularmente) [Condoms are too expensive to use regularly.] |
| 124 | p34 | Cuando usted era niño/a, ¿En su familia se conversaban temas sexuales? [When you were a child, were sexual topics discussed in your family?] |
| 125 | p35 | (CONTESTAN P34=2,3) ¿Y con qué frecuencia usted participaba cuando se conversaban temas sexuales? [And how often did you participate in conversations about sexual topics?] |
| 126 | t_p36_1 | En su escuela, cuándo usted era estudiante, ¿se impartía educación sexual en Enseñanza básica? [When you were a student, was sex education taught in primary school?] |
| 127 | t_p36_2 | En su escuela, cuándo usted era estudiante, ¿se impartía educación sexual en Enseñanza media? [When you were a student, was sex education taught in high school?] |
| 128 | p37 | Y respecto de esa educación sexual, en general, usted cree que... [And regarding that sex education, in general, do you think that...] |
| 129 | p38 | ¿Cómo evaluaría EN GENERAL la FORMACIÓN EN SEXUALIDAD que recibió en su colegio o escuela? [How would you evaluate the SEXUALITY EDUCATION you received at school overall?] |
| 130 | i_1_p39 | ¿Qué nota le podría ahora al conocimiento que usted tenía en los siguientes temas, cuando era adolescente, donde 1 es bajo conocimiento y 7 alto conocimiento? (Métodos para prevenir un embarazo) [What grade would you give your knowledge as a teenager on the following topic: methods to prevent pregnancy? (1 = low, 7 = high)] |
| 131 | i_2_p39 | (Métodos para prevenir infecciones de transmisión sexual) ¿Qué nota le pondría a [What grade would you give your knowledge as a teenager on the following topic: methods to prevent sexually transmitted infections?] |
| 132 | i_1_p40_o1 | Cuando usted era adolescente, a raíz de alguna duda sobre los siguientes temas, ¿A quién recurrió para resolverlas? Relaciones sexuales [As a teenager, when you had questions about the following topics, who did you go to for answers? Sexual relations] |
| 133 | i_2_p40_o1 | Cuando usted era adolescente, a raíz de alguna duda sobre los siguientes temas, ¿A quién recurrió para resolverlas? (Métodos preventivos de embarazo) [As a teenager, when you had questions about the following topics, who did you go to for answers? (Pregnancy prevention methods)] |
| 134 | i_3_p40_o1 | Cuando usted era adolescente, a raíz de alguna duda sobre los siguientes temas, ¿A quién recurrió para resolverlas? (Métodos preventivos de infecciones de transmisión sexual, como el VIH) [As a teenager, when you had questions about the following topics, who did you go to for answers? (STI prevention methods, such as HIV)] |
| 135 | i_1_p41 | ¿Qué tan de acuerdo o en desacuerdo está con que la educación sexual en Chile tenga las siguientes características? Que promueva la abstinencia sexual hasta, a lo menos, los 18 años. [To what extent do you agree or disagree that sex education in Chile should include: Promoting sexual abstinence until at least 18 years old.] |
| 136 | i_2_p41 | (Que transmita una visión positiva del placer y el disfrute sexual.) [Conveying a positive view of pleasure and sexual enjoyment.] |
| 137 | i_3_p41 | (Que enseñe que solo un hombre y una mujer pueden conformar una familia) [Teaching that only a man and a woman can form a family.] |
| 138 | i_4_p41 | (Que enseñe a poner un preservativo o condón) [Teaching how to put on a condom.] |
| 139 | i_5_p41 | (Que enseñe que se tiene que acordar claramente que se quiere tener relaciones sexuales con la otra persona) [Teaching that there must be clear mutual agreement to have sex.] |
| 140 | p42 | (Si usted es mujer) ¿A qué edad aproximadamente tuvo su primera menstruación o regla? [If you are a woman: At approximately what age did you have your first menstruation or period?] |
| 141 | p43 | (Si es hombre) ¿A qué edad aproximadamente tuvo su primera eyaculación nocturna? [If you are a man: At approximately what age did you have your first nocturnal ejaculation?] |
| 142 | p44 | (Para mujeres) ¿Usted conversó con alguien sobre su primera menstruación o regla en ese momento? [Did you talk to anyone about your first menstruation or period at that time?] |
| 143 | p45 | (Para hombres) ¿Usted conversó con alguien sobre su primera eyaculación nocturna en ese momento? [Did you talk to anyone about your first nocturnal ejaculation at that time?] |
| 144 | p46 | ¿Qué edad tenía usted cuando dio su primer beso en la boca con connotación romántico o sexual? [How old were you when you had your first kiss on the mouth with romantic or sexual connotation?] |
| 145 | t_p47_1 | ¿Alguna vez en su vida ha tenido relaciones sexuales voluntarias? [Have you ever had voluntary sexual intercourse in your life?] |
| 146 | t_p47_2 | ¿Alguna vez en su vida ha tenido las siguientes prácticas sexuales voluntarias? Sexo vaginal [Have you ever had the following voluntary sexual practices in your life? Vaginal sex] |
| 147 | t_p47_3 | ¿Alguna vez en su vida ha tenido las siguientes prácticas sexuales voluntarias? Sexo anal [Have you ever had the following voluntary sexual practices in your life? Anal sex] |
| 148 | p48 | ¿Alguna vez en su vida ha tenido las siguientes prácticas sexuales voluntarias? Sexo oral [Have you ever had the following voluntary sexual practices in your life? Oral sex] |
| 149 | p49 | (Para quienes no han tenido relaciones sexuales) ¿Ha recibido caricias en el cuerpo, caricias en pechos o caricias genitales? [Have you received caresses on your body, breasts, or genitals?] |
| 150 | p50_o1 | ¿A qué edad tuvo su primera relación sexual voluntaria? [At what age did you have your first voluntary sexual intercourse?] |
| 151 | p51 | ¿Esta primera relación sexual, de qué forma fue? Sexo vaginal [What form did your first sexual intercourse take? Vaginal sex] |
| 152 | p52 | ¿La persona con quien tuvo esa primera relación sexual era hombre, mujer, otro? [Was the person with whom you had your first sexual intercourse a man, woman, or other?] |
| 153 | p53 | ¿Y la otra persona, qué edad tenía? [And how old was the other person?] |
| 154 | p54 | Antes de su primera relación sexual, ¿Usted y esa persona hablaron de cómo evitar un posible embarazo? [Before your first sexual intercourse, did you and that person talk about how to avoid a possible pregnancy?] |
| 155 | p55 | Antes de su primera relación sexual, ¿Usted y esa persona hablaron de cómo evitar una ITS…? [Before your first sexual intercourse, did you and that person talk about how to prevent an STI?] |
| 156 | p56 | En esa primera relación sexual, ¿Ustedes usaron algún método anticonceptivo? [Did you use any contraceptive method during your first sexual intercourse?] |
| 157 | p57 | ¿Cuál o cuáles métodos anticonceptivos usaron en esa primera relación sexual? MARQUE TODAS LAS QUE CORRESPONDA. [Which contraceptive method(s) did you use during your first sexual intercourse? MARK ALL THAT APPLY.] |
| 158 | p58 | ¿Cuál fue el principal motivo por el cual usaron preservativo o condón? [What was the main reason you used a condom?] |
| 159 | p59 | ¿Dónde obtuvo el método o los métodos mencionados anteriormente? [Where did you get the method(s) mentioned above?] |
| 160 | p60 | ¿En qué lugar tuvo su primera relación sexual? [Where did your first sexual intercourse take place?] |
| 161 | p61 | ¿Era la primera relación sexual también para la otra persona? [Was it also the other person's first sexual experience?] |
| 162 | p62 | ¿Qué tipo de vínculo tenía usted en ese momento con la persona con quien tuvo esa primera relación sexual? (LEA) [What was your relationship with the person with whom you had your first sexual intercourse? (READ)] |
| 163 | p63 | ¿Su primera relación sexual fue principalmente por…?: MARQUE UNA ALTERNATIVA [Your first sexual intercourse was mainly because…?: MARK ONE OPTION] |
| 164 | p64 | Después de la primera relación sexual, ¿Usted tuvo otras relaciones sexuales con la misma persona? [After the first sexual intercourse, did you have sex again with the same person?] |
| 165 | p65 | Con la persona con la que usted, tuvo esa primera relación sexual ¿Llegaron a vivir en pareja? [Did you end up living as a couple with the person with whom you had your first sexual intercourse?] |
| 166 | p66 | (CONTESTAN P65=1) ¿Cuánto tiempo duró la relación de pareja con esta persona? (RECORDAR QUE ES EN RELACIÓN CON LA PAREJA DE LA PRIMERA RELACIÓN SEXUAL) [How long did your relationship with this person last? (REMEMBER: This refers to the partner of your first sexual intercourse)] |
| 167 | p67 | En los seis meses siguientes a esa relación sexual, ¿habló con alguien que no fuera su pareja respecto de esa primera relación sexual? [In the six months after your first sexual intercourse, did you talk to someone other than your partner about it?] |
| 168 | p68 | ¿Con quién habló primero sobre esa primera relación sexual? MARQUE UNA ALTERNATIVA [Who did you talk to first about your first sexual intercourse? MARK ONE OPTION] |
| 169 | p69 | En el transcurso de toda su vida, ¿podría indicar con cuántas personas ha tenido relaciones sexuales? Se trata de personas con las que tuvo relaciones sexuales, aun cuando no hayan sido «pareja». Se incluyen parejas ocasionales, parejas de una sola noche y trabajadoras/es sexuales (“prostitutas/os”). No olvidar a la pareja actual. Si no recuerda con exactitud, por favor indique un número aproximado. [Over the course of your life, how many people have you had sexual relations with? This includes people who were not your partner—such as one-night stands, casual partners, and sex workers ("prostitutes"). Don’t forget your current partner. If you don’t remember exactly, please give an approximate number.] |
| 170 | p70 | (CONTESTAN P69>0) En el transcurso de toda su vida, ¿cuántos han sido hombres, mujeres, u otro [Of the sexual partners you've had in your life, how many were men, women, or other?] |
| 171 | p71 | En el transcurso del último año, ¿podría indicar con cuántas personas han tenido relaciones sexuales? Se trata de personas con las que se tuvo relaciones sexuales, aun cuando no hayan constituido «pareja». Se incluyen parejas ocasionales, parejas de una sola noche y trabajadoras/es sexuales (“prostitutas/os”). No olvidar a la pareja actual. Si no recuerda con exactitud, por favor indique un número aproximado. [In the past year, how many people have you had sexual relations with? This includes people who were not your partner—such as one-night stands, casual partners, and sex workers ("prostitutes"). Don’t forget your current partner. If you don’t remember exactly, please give an approximate number.] |
| 172 | p72 | (CONTESTAN P71>0 Y EN P70 SE MARCA MÁS DE UN SEXO) En el transcurso del último año, ¿cuántos han sido hombres, mujeres, u otro? [In the past year, how many of those sexual partners were men, women, or other?] |
| 173 | p73 | (CONTESTAN P71>0) En las relaciones con esas parejas sexuales del último año, ¿con qué frecuencia usted usaba condón o preservativo? (LEA LAS ALTERNATIVAS) [With those sexual partners from the past year, how often did you use condoms? (READ THE OPTIONS)] |
| 174 | p74 | (CONTESTAN P71>0) En el último mes, ¿podría indicar con cuántas personas ha tenido relaciones sexuales? Se trata de personas con las que tuvo relaciones sexuales, aun cuando no hayan sido «pareja». Se incluyen parejas ocasionales, parejas de una sola noche y trabajadoras/es sexuales (“prostitutas/os”). No olvidar a la pareja actual. Si no recuerda con exactitud, por favor indique un número aproximado. [In the last month, could you indicate how many people you had sexual intercourse with? This includes people with whom you had sexual intercourse even if they were not a “partner.” It includes casual partners, one-night stands, and sex workers (“prostitutes”). Don't forget your current partner. If you don’t remember exactly, please provide an approximate number.] |
| 175 | p75 | (CONTESTAN P74>0 Y EN P72 SE MARCA MÁS DE UN SEXO) En el último mes, ¿cuántos han sido hombres, mujeres, u otro? [In the last month, how many were men, women, or other?] |
| 176 | p76 | (CONTESTAN P74>0) En el último mes, ¿cuántas relaciones sexuales tuvo? Considere número de relaciones sexuales (no parejas sexuales) (Si no ha tenido marcar 0). [In the last month, how many times did you have sexual intercourse? Count the number of sexual acts (not sexual partners). (If none, mark 0).] |
| 177 | p77 | En relación con la cantidad de relaciones sexuales que tuvo el último mes, ¿cuál de las siguientes afirmaciones lo/la representa mejor? [Regarding the number of sexual encounters you had last month, which of the following statements best represents you?] |
| 178 | p78 | En el transcurso de toda su vida, ¿podría indicar con cuántas personas ha tenido una relación afectiva importante, hayan o no vivido juntos? (pololos/as, novios/as, esposo/a, etc.) [Over the course of your life, how many people have you had a significant romantic relationship with, whether or not you lived together? (boyfriends/girlfriends, partners, spouses, etc.)] |
| 179 | p79 | En el transcurso de toda su vida, ¿cuántos han sido hombres, mujeres, u otro? [Throughout your life, how many have been men, women, or other?] |
| 180 | p80 | De ellas, ¿con cuántas ha vivido en pareja durante al menos 3 meses? Considere convivencia sin acuerdo de unión civil, convivencia con acuerdo de unión civil y matrimonio. [Of them, how many have you lived with as a couple for at least 3 months? Consider cohabitation without civil union, with civil union agreement, and marriage.] |
| 181 | p81 | Actualmente, ¿Usted tiene pareja? [Do you currently have a partner?] |
| 182 | p82 | ¿Desde qué año usted tiene esa pareja? [Since what year have you had this partner?] |
| 183 | p83 | (CONTESTA SI P81=1) ¿Y actualmente usted vive con esa pareja? [And do you currently live with this partner?] |
| 184 | p84 | (CONTESTA SI P83 = 1) ¿Desde qué año viven juntos? [Since what year have you been living together?] |
| 185 | p85 | (CONTESTA SI P83 = 2) ¿Usted tiene la intención de vivir junto a su pareja algún día? [Do you intend to live with your partner someday?] |
| 186 | p86 | (CONTESTA SI P81 = 1) Su relación de pareja actual se caracteriza por ser… [Your current relationship is characterized by being...] |
| 187 | p87 | ¿En los últimos 5 años ha tenido alguna separación o ruptura de una relación amorosa? [In the last 5 years, have you experienced a separation or breakup of a romantic relationship?] |
| 188 | p88 | ¿Volvió a tener relaciones sexuales con esa persona después de la separación? (Enc: en caso de haber tenido más de una pareja de la cual se separó, responder respecto a la más importante para la persona entrevistada). [Did you have sexual intercourse with that person again after the separation? (Note for interviewer: if the person had more than one partner from whom they separated, refer to the most important one for the interviewee).] |
| 189 | p89 | Y en la primera relación sexual que volvió a tener con (NOMBRE PAREJA SEPARACIÓN) después de la separación ¿usaron condón o preservativo? [And in the first sexual encounter you had again with (NAME OF SEPARATED PARTNER) after the separation, did you use a condom?] |
| 190 | p90 | (CONTESTA SI P81=1) Ahora hablaremos de la persona con la que usted vive, o bien, con quién tiene una relación estable e importante, aunque no vivan juntos. ¿Esa persona es hombre, mujer u otro? [Now let's talk about the person you live with or with whom you have a stable and significant relationship, even if you don’t live together. Is that person a man, woman, or other?] |
| 191 | p91 | ¿Qué edad tiene esa pareja? (INDICAR EDAD EN AÑOS CUMPLIDOS) [How old is your partner? (INDICATE AGE IN COMPLETED YEARS)] |
| 192 | p92 | (CONTESTA SI P81=1) Comparando con su pareja actual, el ingreso económico de Usted es: [Compared to your current partner, your economic income is:] |
| 193 | p93 | (CONTESTA SI P81=1) ¿Diría que usted depende económicamente de su pareja? [Would you say you are economically dependent on your partner?] |
| 194 | p94 | (CONTESTA SI P83=1) Actualmente, ¿Usted duerme…? (LEA ALTERNATIVAS) [Currently, do you sleep...? (READ ALTERNATIVES) (ASKED IF P83=1)] |
| 195 | p95 | ¿Usted y su pareja hablan o han hablado sobre si desean o desearon tener hijos/as? [Have you and your partner talked or ever talked about whether you want(ed) to have children?] |
| 196 | p96 | (CONTESTA SI P81=1) En el último mes, ¿tuvo relaciones sexuales con su pareja? [In the past month, have you had sexual intercourse with your partner? (ASKED IF P81=1)] |
| 197 | p97 | (CONTESTA SI P81=1 & P96=2) ¿Eso es así por qué…? [Is that so because...? (ASKED IF P81=1 & P96=2)] |
| 198 | p98 | En el transcurso de su vida, ¿alguna vez se ha masturbado? [In your lifetime, have you ever masturbated?] |
| 199 | p99 | ¿A qué edad se masturbó Usted por primera vez? Si no recuerda, mencione una edad aproximada [At what age did you masturbate for the first time? If you don’t remember, mention an approximate age] |
| 200 | p100 | En el último mes, ¿cuántas veces se ha masturbado aproximadamente? [In the past month, approximately how many times have you masturbated?] |
| 201 | p101 | En los últimos 12 meses ¿Con qué frecuencia usted ha realizado las siguientes prácticas en sus relaciones sexuales? [In the past 12 months, how often have you practiced the following during your sexual relations?] |
| 202 | p102 | En el transcurso de su vida…. [In your lifetime....] |
| 203 | p103 | (CONTESTAN SI P74 >0. DROGAS SE FILTRAN SEGÚN REPORTADO EN P25) Justo antes o al momento de las relaciones sexuales que ha tenido en el último mes, ¿usted consumió alguna de las siguientes sustancias? [Just before or at the time of the sexual intercourse you had in the past month, did you consume any of the following substances? (ASKED IF P74>0, FILTERED BY P25 DRUGS)] |
| 204 | p104 | (CONTESTAN SI P74 >0) Y alguna de las personas con la que usted estaba, ¿había consumido alguna de las siguientes sustancias? (MULTIPLE) [And did any of the persons you were with consume any of the following substances? (MULTIPLE OPTIONS) (ASKED IF P74>0)] |
| 205 | p105 | En los últimos 12 meses, ¿Con qué frecuencia ha visto material pornográfico (tales como películas, videos, imágenes, etc.)? [In the past 12 months, how often have you watched pornographic material (such as movies, videos, images, etc.)?] |
| 206 | p106 | ¿Alguna vez en su vida usted ha enviado imágenes o videos suyas con contenido sexual o erótico por algún medio digital (mensaje de texto, e-mail, mensaje directo, WhatsApp, etc.) [Have you ever in your life sent images or videos of yourself with sexual or erotic content through any digital means (text message, e-mail, direct message, WhatsApp, etc.)?] |
| 207 | p107 | ¿Alguna vez en su vida usted ha recibido imágenes de conocidos con contenido sexual o erótico por algún medio digital (mensaje de texto, e-mail, mensaje directo, WhatsApp, etc.)? [Have you ever in your life received sexual or erotic content from people you know through digital means (text message, e-mail, direct message, WhatsApp, etc.)?] |
| 208 | p108 | ¿Alguna vez en su vida ha usado video llamadas (mostrando sus genitales, pechos, o realizando alguna actividad sexual)? [Have you ever in your life used video calls to show your genitals, breasts, or perform any sexual activity?] |
| 209 | p109 | En el último año, ¿ha buscado parejas afectivas y/o sexuales por medio de aplicaciones o plataformas de encuentro digitales? [In the past year, have you searched for romantic and/or sexual partners through digital platforms or apps?] |
| 210 | p110 | (CONTESTA SI P109=1) ¿Tuvo relaciones sexuales con esta o estas personas? [Did you have sexual intercourse with this or these people? (ASKED IF P109=1)] |
| 211 | p111 | ¿Alguna vez ha pagado para tener relaciones sexuales? [Have you ever paid to have sex?] |
| 212 | p112 | (CONTESTA SI P111=1) ¿Qué edad tenía en ese momento? [How old were you at that time? (ASKED IF P111=1)] |
| 213 | p113 | (CONTESTA SI P111=2) ¿Qué edad tenía la primera vez? Y ¿Qué edad tenía la última vez? [How old were you the first time? And how old were you the last time? (ASKED IF P111=2)] |
| 214 | p114 | (CONTESTA SI P111=2) ¿Usted tenía pareja la última vez que pagó para tener relaciones sexuales? [Did you have a partner the last time you paid for sex? (ASKED IF P111=2)] |
| 215 | p115 | Vamos a hablar ahora de su última relación sexual. ¿Cuándo fue su última relación sexual? PARA ENTREVISTADO/AR/A: PUEDE SER LA PAREJA U OTRA PERSONA. [Let's now talk about your last sexual encounter. When was your last sexual encounter? FOR RESPONDENT: IT MAY BE WITH A PARTNER OR SOMEONE ELSE.] |
| 216 | p116 | ¿Qué tipo de vínculo tenía usted en ese momento con la persona con quien tuvo esa última relación sexual? (LEA ALTERNATIVAS) [What kind of relationship did you have at the time with the person you had that last sexual encounter with? (READ ALTERNATIVES)] |
| 217 | p117 | ¿La persona con quien tuvo esa última relación sexual era hombre, mujer, otro? [Was the person you had your last sexual encounter with a man, woman, or other?] |
| 218 | p118 | En cuanto a su género, ¿esa persona es?: [Regarding their gender, is that person?:] |
| 219 | p119 | En esa última relación sexual, ¿Ustedes usaron alguno método anticonceptivo? [In that last sexual encounter, did you use any contraceptive method?] |
| 220 | p120 | ¿Cuál o cuáles métodos anticonceptivos utilizaron? MARQUE TODAS LAS QUE CORRESPONDAN [Which contraceptive methods did you use? MARK ALL THAT APPLY] |
| 221 | p121 | (CONTESTAN P120=1,2) ¿Por qué motivo usaron preservativo o condón? [Why did you use a condom? (ASKED IF P120=1,2)] |
| 222 | p122 | ¿Dónde obtuvo el método o los métodos mencionados anteriormente? [Where did you obtain the method(s) mentioned above?] |
| 223 | p123 | (CONTESTAN SI P119=2) ¿Por qué razón no usó ningún método anticonceptivo? [Why didn’t you use any contraceptive method? (ASKED IF P119=2)] |
| 224 | p124 | ¿Tuvo alguna dificultad para conseguir o acceder a métodos anticonceptivos durante la pandemia? [Did you have any difficulty getting or accessing contraceptive methods during the pandemic?] |
| 225 | p125 | ¿Esta última relación sexual, de qué forma fue? MARQUE TODAS LAS QUE CORRESPONDAN. [What sexual practices were involved in this last sexual encounter? MARK ALL THAT APPLY.] |
| 226 | p126 | [SOLO PARA 125=3] ¿Usted le hizo sexo oral a esta persona? [Did you perform oral sex on this person? (ONLY IF 125=3)] |
| 227 | p127 | [SOLO PARA 125=3] ¿Esta persona le hizo sexo oral a usted? [Did this person perform oral sex on you? (ONLY IF 125=3)] |
| 228 | p128 | Sexo vaginal Sexo anal Sexo Oral Prefiero no responder [Vaginal sex Anal sex Oral sex Prefer not to answer] |
| 229 | p129 | Y la otra persona, ¿Alcanzó un orgasmo? [Did the other person reach orgasm?] |
| 230 | p130 | ¿Y qué tan satisfecho está con esta última relación sexual? [And how satisfied are you with this last sexual encounter?] |
| 231 | p131 | (CONTESTAN SI P115=5) ¿Cuál es el principal motivo por el que usted no ha tenido relaciones sexuales en el último año? [What is the main reason you have not had sex in the past year? (ASKED IF P115=5)] |
| 232 | p132 | (CONTESTAN SI P115=4) ¿Qué diría sobre la situación de no tener relaciones sexuales en el último año? [What would you say about not having had sex in the past year? (ASKED IF P115=4)] |
| 233 | p133 | Durante su vida, usted se ha sentido atraído/a sexualmente por… Por favor no lea, solo indíqueme el número de la tarjeta. (ENCUESTADOR: SOLO PIDA NÚMERO DE RESPUESTA AL ENTREVISTADO/A) [In your lifetime, have you felt sexually attracted to... Please don’t read aloud, just tell me the number on the card. (INTERVIEWER: ONLY ASK FOR THE RESPONSE NUMBER FROM THE RESPONDENT)] |
| 234 | p134 | Usted actualmente se identifica cómo: [How do you currently identify yourself?] |
| 235 | p135 | Durante su vida, ¿ha tenido una orientación sexual diferente de la que recién me comentó (homosexual, heterosexual bisexual u otro)? [During your life, have you ever had a sexual orientation different from the one you just mentioned (homosexual, heterosexual, bisexual, or other)?] |
| 236 | p136 | (CONTESTAN P134 = 1, 2 o 3) ¿A qué edad, se sintió sexualmente atraído, por primera vez, por alguien de su mismo sexo? [At what age did you first feel sexually attracted to someone of the same sex? (ASKED IF P134 = 1, 2 or 3)] |
| 237 | p137 | (CONTESTAN P134 = 1, 2 o 3) ¿Alguna vez recibió tratamiento de alguien que intentó cambiar su orientación sexual (como tratar de volverse heterosexual)? En caso afirmativo, marque todas las alternativas que correspondan. [Have you ever received treatment from someone who tried to change your sexual orientation (such as trying to make you heterosexual)? If yes, mark all that apply. (ASKED IF P134 = 1, 2 or 3)] |
| 238 | p138 | (CONTESTAN P3 = 3, 4 Y 5) Anteriormente me mencionó que no se identifica con el género que le asignaron en su nacimiento. ¿A qué edad comenzó a sentir que su género era “diferente” al que le asignaron en su nacimiento? [Earlier you mentioned that you do not identify with the gender assigned to you at birth. At what age did you begin to feel that your gender was “different” from the one assigned at birth? (ASKED IF P3 = 3, 4 or 5)] |
| 239 | p139 | (CONTESTAN P3= 3 o 4) respecto a su identidad de género, ¿diría que está o estuvo en proceso de transición? [Regarding your gender identity, would you say you are or have been in the process of transition? (ASKED IF P3 = 3 or 4)] |
| 240 | p140 | (CONTESTAN P3= 3 o 4) ¿Desde hace cuántos años está en proceso de transición? (indique cantidad de años aproximado) [For how many years have you been in the process of transition? (Indicate approximate number of years) (ASKED IF P3 = 3 or 4)] |
| 241 | p141 | (CONTESTAN P3 = 3 o 4 & P139 = 1) Actualmente, ¿ha tenido o ha comenzado? [Currently, have you had or begun...? (ASKED IF P3 = 3 or 4 & P139 = 1)] |
| 242 | p142 | (CONTESTAN P3 = 3 o 4 & P141.1 = 1) ¿Ha efectuado su cambio de nombre y sexo en el Registro Civil? [Have you changed your name and sex in the Civil Registry? (ASKED IF P3 = 3 or 4 & P141.1 = 1)] |
| 243 | p143 | (CONTESTAN P3 = 3 o 4 & 141.2 = 1) Si está tomando hormonas, ¿por dónde las obtiene? (marque todas las que corresponde) [If you are taking hormones, where do you get them from? (Mark all that apply) (ASKED IF P3 = 3 or 4 & P141.2 = 1)] |
| 244 | p144 | (CONTESTAN P3 = 3 o 4 & 141.3 = 1) ¿Ha tenido operaciones o intervenciones quirúrgicas relacionadas con una reasignación de sexo o transición de género? [Have you had surgeries or procedures related to sex reassignment or gender transition? (ASKED IF P3 = 3 or 4 & P141.3 = 1)] |
| 245 | p145 | (CONTESTAN P144 = 1) ¿Cuál/es es/son la/s operación/es o intervención/es de otra índole que se ha realizado? (Puedes marcar más de una alternativa) [Which surgery or other procedures have you undergone? (You can mark more than one) (ASKED IF P144 = 1)] |
| 246 | p146 | (CONTESTAN P144 = 1) ¿Dónde se realizó esas operaciones o intervenciones quirúrgicas? (Puedes marcar más de una alternativa) [Where did you have those surgeries or procedures performed? (You can mark more than one) (ASKED IF P144 = 1)] |
| 247 | p147 | (CONTESTAN P3 = 3 o 4 & P141.1 = 1 \| P141.2 = 1 \| P141.3 = 1) Durante su proceso de transición ¿ha tenido la necesidad de una consulta de diagnóstico para solicitar certificado de disforia de género con algún profesional de salud (médico psiquiatra o psicólogo/a)? [During your transition, have you needed a diagnostic consultation to request a gender dysphoria certificate from a health professional (psychiatrist or psychologist)? (ASKED IF P3 = 3 or 4 & P141.1/2/3 = 1)] |
| 248 | p148 | (CONTESTAN P3 = 3º 4) ¿Alguna vez recibió tratamiento de alguien que intentó hacerle identificarse solo con su sexo asignado al nacer (en otras palabras, tratar de evitar que usted fuera transgénero)? En caso afirmativo, marque todas las casillas que correspondan. [Have you ever received treatment from someone who tried to make you identify only with your birth-assigned sex (i.e., to prevent you from being transgender)? If yes, check all that apply. (ASKED IF P3 = 3 or 4)] |
| 249 | p149 | (CONTESTAN P3 = 3, 4, 5, 6, 7 o P134 = 1, 3, 4) A continuación, te presentamos varias frases que describen diversas experiencias de vida y cosas que pueden haberte ocurrido por ser una persona LGTBQ+. Desde que tenías 18 años, ¿Alguna vez…? [Here are several statements describing life experiences that may have happened to you as an LGBTQ+ person. Since you were 18, have you ever...? (ASKED IF P3 = 3, 4, 5, 6, 7 or P134 = 1, 3, 4)] |
| 250 | p150 | [SOLO PARA P149 = 1] ¿Y cuántas veces ha sucedido en los últimos 12 meses? [How many times has this happened in the last 12 months? (ONLY IF P149 = 1)] |
| 251 | p151 | (RESPONDE SEXO = MUJER) ¿Alguna vez en su vida ha ido a una consulta o donde algún profesional de la salud para tratar asuntos médicos PROPIOS relacionados con la ginecología, pubertad, sexualidad, métodos preventivos del embarazo o infecciones de transmisión sexual? [Have you ever in your life visited a health professional to address personal medical issues related to gynecology, puberty, sexuality, pregnancy prevention methods, or sexually transmitted infections? (RESPONDENT SEX = FEMALE)] |
| 252 | p152 | (RESPONDE SEXO = HOMBRE) ¿Alguna vez en su vida ha ido a una consulta o donde algún profesional de la salud para tratar asuntos médicos PROPIOS relacionados con la urología, pubertad, sexualidad, métodos preventivos del embarazo o infecciones de transmisión sexual? [Have you ever in your life visited a health professional to address personal medical issues related to urology, puberty, sexuality, pregnancy prevention methods, or sexually transmitted infections? (RESPONDENT SEX = MALE)] |
| 253 | p153 | ¿Qué edad tenía la primera vez que consultó?: [How old were you the first time you sought such a consultation?] |
| 254 | p154 | ¿Por qué motivo fue esa primera vez? (MARQUE TODAS LAS QUE CONSIDERE) [What was the reason for that first time? (MARK ALL THAT APPLY)] |
| 255 | p155 | ¿A qué lugar fue? (LEA ALTERNATIVAS. CIRCULE SOLO UNO) [Where did you go? (READ OPTIONS. CIRCLE ONLY ONE)] |
| 256 | p156 | [SOLO PARA SEXO = MUJER] ¿Alguna vez en su vida ha tenido un control o consulta ginecológica? [FOR WOMEN ONLY: Have you ever had a gynecological check-up or consultation?] |
| 257 | p157 | [SOLO PARA SEXO ASIGNADO AL NACER = MUJER] Te pedimos que nos indiques si, en el marco de tus visitas a servicios de ginecología (públicos o privados), con qué frecuencia le ha ocurrido alguna de las siguientes situaciones. [FOR PEOPLE ASSIGNED FEMALE AT BIRTH ONLY: Please indicate how often the following situations have occurred during your visits to gynecological services (public or private).] |
| 258 | p158 | ¿Ha estado embarazada alguna vez en su vida? (incluir embarazos que terminaron en parto normal, parto prematuro, aborto, muerte fetal u otro problema). [Have you ever been pregnant? (Include pregnancies ending in full-term delivery, preterm delivery, abortion, stillbirth, or other complications).] |
| 259 | p159 | ¿Está embarazada actualmente? [Are you currently pregnant?] |
| 260 | p160 | ¿Cuántos embarazos ha tenido a lo largo de su vida? incluya embarazos que terminaron en parto normal, parto prematuro, aborto, muerte fetal u otro problema. [How many pregnancies have you had in your lifetime? Include pregnancies ending in full-term delivery, preterm delivery, abortion, stillbirth, or other complications.] |
| 261 | p161 | ¿Uno o más de los embarazos que tuvo no fue planificado o no estaba previsto que ocurriera? [Was one or more of your pregnancies unplanned or unexpected?] |
| 262 | p162 | ¿Cuántos embarazos fueron no planificados? [How many pregnancies were unplanned?] |
| 263 | p163 | ¿Cuál o cuáles? [Which one(s)?] |
| 264 | p164 | (CONTESTAN SI P161=1) ¿Qué edad tenía usted al momento que se enteró del primer embarazo que no fue planificado o no había previsto que ocurriera? [(IF P161=1) How old were you when you found out about your first unplanned or unexpected pregnancy?] |
| 265 | p165 | (CONTESTAN SI P161=1) En el momento en que se enteró de este embarazo no planificado o no previsto ¿Qué relación tenía usted con la persona con la que tuvo este embarazo? [(IF P161=1) At the time you found out about this unplanned pregnancy, what was your relationship with the person you had the pregnancy with?] |
| 266 | p166 | (CONTESTAN SI P161=1) ¿Usted o esa persona usaban un método preventivo del embarazo en el momento en que tuvo ese primer embarazo que no fue planificado o no estaba previsto que ocurriera? [(IF P161=1) Were you or that person using any pregnancy prevention method when you had that first unplanned pregnancy?] |
| 267 | p167 | ¿Qué edad tenía usted cuando ocurrió el primer embarazo? [How old were you when the first pregnancy occurred?] |
| 268 | p168 | ¿Quién era la persona de la cual se embarazó? Considere la relación que tenía usted con esa persona en el momento del embarazo. [Who was the person you got pregnant by? Consider the relationship you had with that person at the time of the pregnancy.] |
| 269 | p169 | ¿Y cuántos/as hijos/as nacidos vivos/as ha tenido Usted? [How many live-born children have you had?] |
| 270 | p170 | (SI TIENE HIJOS/AS) ¿Qué edad tenía usted cuando nació su primer/a hijo/a? [IF YOU HAVE CHILDREN: How old were you when your first child was born?] |
| 271 | p171 | (SI TIENE HIJOS/AS) ¿Con cuántas personas distintas ha tenido hijos/as? [IF YOU HAVE CHILDREN: How many different people have you had children with?] |
| 272 | p172 | (SI TIENE HIJOS/AS), ¿Cuántos de sus hijos viven con usted? [IF YOU HAVE CHILDREN: How many of your children live with you?] |
| 273 | p173 | ¿En este hogar hay niños menores de 3 años a su cuidado? [Are there children under 3 years old in this household under your care?] |
| 274 | p174 | Ahora vamos a conversar sobre el último embarazo que tuvo. ¿Se realizó un control prenatal, se chequeó el embarazo alguna vez? [Now we are going to talk about your last pregnancy. Did you have a prenatal check-up, did you have the pregnancy checked at any time?] |
| 275 | p175 | ¿En qué semana de su último embarazo tuvo el primer control? [In which week of your last pregnancy did you have your first check-up?] |
| 276 | p176 | ¿Cuántos controles prenatales tuvo Ud. durante el último embarazo? [How many prenatal check-ups did you have during your last pregnancy?] |
| 277 | p177 | ¿Durante su último embarazo con quién se controló? (MARQUE TODAS LAS QUE CONSIDERE) [During your last pregnancy, who did you consult with for check-ups? (MARK ALL THAT APPLY)] |
| 278 | p178 | ¿Dónde se controló? (MARQUE TODAS LAS QUE CONSIDERE) [Where did you have your check-ups? (MARK ALL THAT APPLY)] |
| 279 | p179 | En alguno de sus controles de su último embarazo, le hicieron o le mandaron a usted lo siguiente: [During any of your check-ups in your last pregnancy, were you given or referred for the following:] |
| 280 | p180 | Su último embarazo terminó con: [Your last pregnancy ended in:] |
| 281 | p181 | (CONTESTAN SI P0=1 o 2) ¿Cuál es la fecha de su último parto? SI NO SABE O NO RECUERDA, COLOCAR 01/01/1900. [(IF P0=1 or 2) What is the date of your last delivery? IF YOU DO NOT KNOW OR DO NOT REMEMBER, ENTER 01/01/1900.] |
| 282 | p182 | (CONTESTAN SI P0=3 o 4) ¿Cuál es la fecha de su última pérdida o aborto? SI NO SABE O NO RECUERDA, COLOCAR 01/01/1900. [(IF P0=3 or 4) What is the date of your last miscarriage or abortion? IF YOU DO NOT KNOW OR DO NOT REMEMBER, ENTER 01/01/1900.] |
| 283 | p183 | (CONTESTAN SI P0=3 o 4) ¿Recibió atención médica durante o inmediatamente después del aborto? [(IF P0=3 or 4) Did you receive medical attention during or immediately after the abortion?] |
| 284 | p184 | (CONTESTAN SI P183 = 1) ¿Dónde recibió la atención médica? [(IF P183 = 1) Where did you receive medical attention?] |
| 285 | p185 | (CONTESTAN SI P0=1 o 2) ¿Dónde fue su último parto? [(IF P0=1 or 2) Where did your last delivery take place?] |
| 286 | p186 | (CONTESTAN SI P0=1 o 2) ¿El parto fue por cesárea? [(IF P0=1 or 2) Was the delivery by cesarean section?] |
| 287 | p187 | (CONTESTAN SI P186 = 1) ¿Esta cesárea fue por…? [(IF P186 = 1) Was this cesarean section due to…?] |
| 288 | p188 | (CONTESTAN SI P0=1 o 2) Una vez dada de alta del último parto, ¿tuvo Ud. algún chequeo o control médico dentro de las 2 semanas posteriores? [(IF P0=1 or 2) After being discharged from your last delivery, did you have any medical check-up within the following 2 weeks?] |
| 289 | p189 | (CONTESTAN SI P188 = 2) ¿Cuál fue la principal razón por qué no tuvo chequeo o revisión médica? [(IF P188 = 2) What was the main reason you did not have a medical check-up?] |
| 290 | p190 | (CONTESTAN SI P180=1 o 2) ¿Algún médico(a) o doctor(a) le diagnosticó depresión postparto? [(IF P180=1 or 2) Did any doctor diagnose you with postpartum depression?] |
| 291 | p191 | (CONTESTAN SI P190 = 1) ¿Tuvo tratamiento para esta depresión postparto? [(IF P190 = 1) Did you receive treatment for this postpartum depression?] |
| 292 | p192 | (CONTESTAN SI P0=1 o 2) ¿Mirando hacia atrás, cree usted que tuvo depresión postparto? [(IF P0=1 or 2) Looking back, do you think you had postpartum depression?] |
| 293 | p193 | (CONTESTAN SI P0=1 o 2) Después de este último parto, ¿usted amamantó a su hijo/a? [(IF P0=1 or 2) After this last delivery, did you breastfeed your child?] |
| 294 | p194 | [SOLO SI P180 = 1 o 2 y P193 = 1] ¿Por cuántos meses amamantó a su hijo/a? [[ONLY IF P180 = 1 or 2 and P193 = 1] For how many months did you breastfeed your child?] |
| 295 | p195 | [SOLO SI P180 = 1 o 2 y P193 = 1] ¿Cree usted que el amamantar afectó a su vida sexual…? LEER ALTERNATIVAS [[ONLY IF P180 = 1 or 2 and P193 = 1] Do you think breastfeeding affected your sex life...? READ OPTIONS] |
| 296 | p196 | (CONTESTAN SI P180 = 1 o 2) En su último parto, ¿a usted…? [(IF P180 = 1 or 2) During your last delivery, were you...?] |
| 297 | p197 | ¿Ha tenido menstruación o regla en el último año? [Have you had your period or menstruation in the past year?] |
| 298 | p198 | ¿Cuál es la razón por la cual usted no ha tenido menstruación o regla en el último año? [What is the reason you have not had your period or menstruation in the past year?] |
| 299 | p199 | (CONTESTAN SI P198 = 4 O 7) ¿Podría indicarme aproximadamente qué edad tenía usted en su última menstruación o regla? [(IF P198 = 4 or 7) Could you tell me approximately how old you were when you had your last period?] |
| 300 | p200 | (CONTESTAN SI P198 = 4 o 7) ¿Alguna vez ha consultado a un médico/a, ginecólogo/a o matrón/a por razones relacionadas con su menopausia? [(IF P198 = 4 or 7) Have you ever consulted a doctor, gynecologist, or midwife for reasons related to your menopause?] |
| 301 | p201 | (CONTESTAN SI P198 = 4 O 7) ¿Ha tomado hormonas (terapia hormonal) para la menopausia? [(IF P198 = 4 or 7) Have you taken hormones (hormone therapy) for menopause?] |
| 302 | p202 | ¿Alguna vez en su vida, un doctor o médico le ha dicho que tiene o que padece de...? [Has a doctor ever told you that you have or suffer from...?] |
| 303 | p203 | ¿A qué edad se lo diagnosticaron por última vez? [At what age were you last diagnosed with this condition?] |
| 304 | p204 | La última vez que usted tuvo esta enfermedad ¿Siguió tratamiento? [The last time you had this illness, did you follow a treatment?] |
| 305 | p205 | ¿Alguna vez ha sido operado por esto? [Have you ever had surgery for this?] |
| 306 | p206 | ¿Cómo descubrió que tenía alguna de esas infecciones? Si ha tenido más de una vez una infección, piense en la última. [How did you find out that you had any of those infections? If you had more than one, think of the last one.] |
| 307 | p207 | La última vez que fue diagnosticado de alguna infección de transmisión sexual, ¿se lo informó a su o sus parejas sexuales? [The last time you were diagnosed with a sexually transmitted infection, did you inform your sexual partner(s)?] |
| 308 | p208 | [RESPONDEN P202.10 = 2] Por cualquier razón, ¿Se ha hecho el examen del VIH o Sida en los últimos 12 meses? [[IF P202.10 = 2] For any reason, have you had an HIV or AIDS test in the last 12 months?] |
| 309 | p209 | [RESPONDEN P202.10 = 2 & P208 = 1] ¿Conoce su resultado? [[IF P202.10 = 2 & P208 = 1] Do you know your result?] |
| 310 | p210 | [SOLO PARA P202.10 = 1 & P208 = 1] ¿Por qué razón se hizo el examen del VIH o Sida? [[ONLY IF P202.10 = 1 & P208 = 1] What was the reason you had an HIV or AIDS test?] |
| 311 | p211 | [SOLO PARA P202.10 = 2 & P208 = 2] ¿Por qué razón no se ha hecho el examen del VIH o Sida en los últimos 12 meses? MARCAR TODAS LAS QUE CORRESPONDA [[ONLY IF P202.10 = 2 & P208 = 2] Why haven’t you had an HIV or AIDS test in the past 12 months? MARK ALL THAT APPLY] |
| 312 | p212 | Ahora le voy a hacer unas preguntas sobre VIH o Sida. Cree usted qué… LEA AL ENCUESTADO CADA PREGUNTA Y MARQUE SÍ O NO [Now I’m going to ask you some questions about HIV or AIDS. Do you believe that… READ EACH QUESTION TO THE RESPONDENT AND MARK YES OR NO] |
| 313 | p213 | ¿Conoce usted la medida “profilaxis de preexposición” o PREP como alternativa de prevención del VIH/Sida? [Do you know about “pre-exposure prophylaxis” or PrEP as an HIV/AIDS prevention measure?] |
| 314 | p214 | SI ES MUJER: Alguna vez en su vida, ¿Usted ha usado anticoncepción de emergencia, llamada píldora del día después o PAE? Por favor, considere solo el uso de la píldora del día después, y no otras píldoras que buscan tener el mismo efecto. [IF FEMALE: Have you ever used emergency contraception, known as the morning-after pill or ECP? Please consider only the use of the morning-after pill, and not other pills that seek the same effect.] |
| 315 | p215 | SI ES HOMBRE: Alguna vez en su vida, ¿En una relación sexual con una mujer, ella ha usado anticoncepción de emergencia llamada píldora del día después o PAE? Por favor, considere solo el uso de la píldora del día después, y no otras píldoras que buscan tener el mismo efecto. [IF MALE: Have you ever been in a sexual relationship with a woman who used emergency contraception, known as the morning-after pill or ECP? Please consider only the use of the morning-after pill, and not other pills that seek the same effect.] |
| 316 | p216 | (CONTESTA SI EN P124 o 215 = 1) En los últimos 12 meses, ¿Cuántas veces ha usado anticoncepción de emergencia? (INDICAR NUMERO DE VECES) Por favor, considere solo el uso de la píldora del día después, y no otras píldoras que buscan tener el mismo efecto. [(IF YES TO P124 OR 215 = 1) In the past 12 months, how many times have you used emergency contraception? (INDICATE NUMBER OF TIMES) Please consider only the morning-after pill.] |
| 317 | p217 | ¿Alguna vez en la vida, alguna de sus parejas ha interrumpido algún embarazo producto de una relación sexual con usted? [Has any of your partners ever terminated a pregnancy that resulted from sexual intercourse with you?] |
| 318 | p218 | ¿Esta decisión fue consensuada con usted? [Was this decision made jointly with your partner?] |
| 319 | p219 | ¿Alguna vez en la vida, usted intentó o realizó alguna acción para interrumpir un embarazo propio, pero sin concretarlo? [Have you ever attempted or taken any action to terminate your own pregnancy, but without completing it?] |
| 320 | p220 | ¿Alguna vez en la vida, usted interrumpió un embarazo propio, concretándolo? [Have you ever successfully terminated your own pregnancy?] |
| 321 | p221 | ¿Ese embarazo lo interrumpió bajo alguna de las causas indicadas en la Ley de Interrupción voluntaria del embarazo? [Was the termination carried out under any of the grounds established in the Law on Voluntary Termination of Pregnancy?] |
| 322 | p222 | ¿Por cuál causal lo interrumpió? [What was the reason or ground for terminating it?] |
| 323 | p223 | ¿Qué edad tenía usted cuando interrumpió ese embarazo? (INDICAR EDAD N AÑOS CUMPLIDOS) [How old were you when you terminated that pregnancy? (INDICATE AGE IN COMPLETED YEARS)] |
| 324 | p224 | ¿Cuántos meses de gestación tenía al momento de interrumpir ese embarazo? (ANOTAR SEMANAS. SI NO RECUERDA SEMANAS; ANOTAR MESES) [How many months pregnant were you when you terminated that pregnancy? (RECORD IN WEEKS; IF YOU DON’T REMEMBER, RECORD IN MONTHS)] |
| 325 | p225 | Cuando usted interrumpió ese embarazo ¿Qué relación tenía usted con la persona con la que se embarazó? [What was your relationship with the person who got you pregnant at the time of the pregnancy?] |
| 326 | p226 | Para interrumpir el embarazo usted puede haber usado uno o más métodos para hacerlo, ¿cuál o cuáles usó? MARQUE TODAS LAS QUE CORRESPONDA [To terminate the pregnancy, you may have used one or more methods. Which one(s) did you use? CHECK ALL THAT APPLY] |
| 327 | p227 | ¿Usted se hizo una ecografía después del aborto? [Did you have an ultrasound after the abortion?] |
| 328 | p228 | ¿Usted tuvo alguna consulta con un profesional de la salud para interrumpir ese embarazo? [Did you consult a health professional before terminating that pregnancy?] |
| 329 | p229 | ¿Tuvo complicaciones que requirieron atención médica en un servicio de salud a raíz del aborto? [Did you experience complications that required medical care after the abortion?] |
| 330 | p230 | ¿Usted tuvo alguna consulta con un profesional de la salud para realizar el seguimiento de la interrupción de ese embarazo? [Did you have a follow-up consultation with a health professional after the abortion?] |
| 331 | p231 | ¿Con quién? [With whom?] |
| 332 | p232 | ¿Se contactó con un colectivo u ONG que colabora con las mujeres en la realización del aborto? [Did you contact any organization or NGO that supports women in accessing abortion services?] |
| 333 | p233 | ¿Su pareja actual ha tenido alguna enfermedad o discapacidad que lo haya afectado a usted en su actividad sexual o el disfrute de la misma? [Has your current partner had any illness or disability in the past 12 months that has affected your sexual activity or enjoyment?] |
| 334 | p234 | En los últimos 12 meses, usted ha tenido alguna enfermedad o discapacidad que lo haya afectado a usted en su actividad sexual o el disfrute de esta? [In the past 12 months, have you had any illness or disability that affected your sexual activity or enjoyment?] |
| 335 | p235 | ¿Usted ha tomado algún medicamento que haya afectado negativamente su actividad sexual o el disfrute de esta? [Have you taken any medication in the past 12 months that negatively affected your sexual activity or enjoyment?] |
| 336 | p236 | ¿Con qué frecuencia usted ha experimentado alguna de las siguientes situaciones en su vida sexual? MARCAR TODAS LAS QUE CORRESPONDAN [How often have you experienced any of the following situations in your sex life? MARK ALL THAT APPLY] |
| 337 | p237 | ¿Esto que le pasa a usted, cree que es un problema para la vida sexual con su pareja? [Do you think this affects your sex life with your partner?] |
| 338 | p238 | ¿Usted ha consultado o pedido ayuda a algún profesional de la salud por las situaciones que mencionó anteriormente? [Have you sought help from a health professional for the situations you mentioned?] |
| 339 | p239 | ¿A qué profesional o profesionales? [Which professional(s)?] |
| 340 | p240 | ¿Alguna vez en su vida ha experimentado las siguientes situaciones en lugares públicos, sin su consentimiento, tales como calles, plazas, transporte público, centros comerciales, cines, estadios, conciertos, marchas u otros espacios similares a estos? [Have you ever experienced the following situations in public spaces without your consent, such as streets, plazas, public transport, shopping centers, cinemas, stadiums, concerts, marches, or other similar places?] |
| 341 | p241 | ¿Le ocurrió en el último año? [Did it happen in the past year?] |
| 342 | p242 | ¿A qué edad le ocurrió por primera vez o única vez esta situación? [At what age did this situation happen to you for the first or only time?] |
| 343 | p243 | ¿En cuál o cuáles de los siguientes lugares le ha ocurrido esa situación alguna vez en la vida? TODAS LAS QUE CORRESPONDAN [In which of the following places has this situation occurred in your life? SELECT ALL THAT APPLY] |
| 344 | p244 | ¿Alguna vez en la vida ha experimentado alguna de las siguientes situaciones? [Have you ever experienced any of the following situations in your life?] |
| 345 | p245 | ¿Qué tan frecuentemente le sucedió esto en los últimos 5 años? [How frequently did this happen to you in the last 5 years?] |
| 346 | p246 | Pensando la última vez que le sucedió ¿Dónde le ocurrió? [Thinking of the last time this happened, where did it occur?] |
| 347 | p247 | ¿Alguna vez en la vida alguien te tocó tus partes privadas, tus genitales o tus pechos, bajo manipulación, engaño, sometimiento u obligación, y/o te forzaron a hacerlo? [Has anyone ever touched your private parts, genitals or breasts under manipulation, deceit, coercion or obligation, and/or forced you to do it?] |
| 348 | p248 | ¿Y en esa oportunidad te penetraron oral, anal o vaginalmente, con el pene, los dedos u otro objeto, bajo manipulación, engaño, sometimiento u obligación, y/o te forzaron a hacerlo? [And at that time, were you penetrated orally, anally or vaginally, with a penis, fingers or another object under manipulation, deceit, coercion or obligation, and/or were you forced to do it?] |
| 349 | p249 | ¿Y esto, cuántas veces le ocurrió? [How many times has this happened to you?] |
| 350 | p250 | ¿Qué edad tenía usted cuando ocurrió esto? [How old were you when this happened?] |
| 351 | p251 | ¿Qué edad tenía usted la primera vez? [How old were you the first time?] |
| 352 | p252 | ¿Qué edad tenía usted la última vez? [How old were you the last time?] |
| 353 | p253 | Pensando en la primera o única vez ¿Quién le hizo esto? [Thinking of the first or only time, who did this to you?] |
| 354 | p254 | (CONTESTA SI P249 = 1,2) Y pensando en la primera o única vez ¿La persona que le hizo esto era hombre o mujer? [If P249 = 1 or 2: Thinking about the first or only time, was the person who did this to you a man or a woman?] |
| 355 | p255 | (CONTESTA SI P249 = 3,4) Y pensando en la primera o única vez ¿Las personas que le hicieron esto eran hombres o mujeres? [If P249 = 3 or 4: Thinking about the first or only time, were the people who did this to you men or women?] |
| 356 | p256 | (CONTESTA SI P249 = 1, 2) Y pensando en la primera o única vez ¿Qué edad aproximadamente tenía la persona que le hizo esto? [If P249 = 1 or 2: Thinking about the first or only time, approximately how old was the person who did this to you?] |
| 357 | p257 | (CONTESTA SI P249 = 3, 4) Y pensando en la primera o única vez ¿Qué edad en promedio tenían las personas que le hicieron esto? [If P249 = 3 or 4: Thinking about the first or only time, what was the average age of the people who did this to you?] |
| 358 | p258 | ¿Ha hablado de esto con alguien? [Have you talked to anyone about this?] |
| 359 | p259 | (RESPONDEN P258 = 1) ¿Y con quién habló de ello? [If answered yes to P258: Who did you talk to about it?] |
| 360 | p260 | ¿Usted realizó una denuncia a la Justicia por este hecho? [Did you report this incident to the justice system?] |
| 361 | p261 | (CONTESTAN SI P260 = 2) ¿Cuál fue la principal razón por la qué no realizó una denuncia a la Justicia? [If P260 = 2: What was the main reason why you did not report it to the justice system?] |
| 362 | p262 | ¿Siente Ud. que esta situación lo(la) afectó de manera significativa en su vida? [Do you feel that this situation significantly affected your life?] |
| 363 | p263 | ¿Cuál es su nacionalidad? [What is your nationality?] |
| 364 | p264 | ¿Cuál? [Which one?] |
| 365 | p265 | Cuándo usted nació, ¿En qué país vivía su madre? [When you were born, in which country was your mother living?] |
| 366 | p266 | ¿Cuál? [Which one?] |
| 367 | p267 | En Chile, la ley reconoce diez pueblos indígenas, ¿pertenece Usted o es descendiente de alguno de ellos? [In Chile, the law recognizes ten Indigenous peoples. Do you belong to or are you a descendant of any of them?] |
| 368 | p268 | ¿Cuál es su religión o credo? [What is your religion or belief?] |
| 369 | p269 | ¿Usted se definiría como una persona…? (LEA ALTERNATIVAS) [How would you define yourself as a person...? (READ ALTERNATIVES)] |
| 370 | p270 | En general, la gente suele situarse en posiciones políticas más cercanas a la izquierda, al centro o a la derecha. En una escala de 1 a 10, donde 1 es izquierda y 10 es derecha, ¿En qué lugar se ubicaría usted? Elija el número que quiera según se inclina más hacia uno u otro lado [In general, people tend to position themselves politically closer to the left, center, or right. On a scale from 1 to 10, where 1 is left and 10 is right, where would you place yourself? Choose the number that best reflects your tendency.] |
| 371 | p271 | ¿Cuántos dormitorios, de uso exclusivo para dormir, tiene su vivienda? (INDICAR EL NUMERO DE PIEZAS) [How many bedrooms, used exclusively for sleeping, does your household have? (INDICATE THE NUMBER OF ROOMS)] |
| 372 | p272 | La semana pasada, ¿usted trabajó al menos una hora, sin considerar los quehaceres del hogar? [Last week, did you work at least one hour, not counting household chores?] |
| 373 | p273 | Aunque no trabajó la semana pasada, ¿usted realizó alguna actividad por lo menos durante una hora? …por un salario o remuneración? ¿en su empresa o negocio? para la empresa o negocio de un familiar (con o sin remuneración)? por pago en especies? como aprendiz o realizando una práctica remunerada? de venta, ¿sin incluir bienes del hogar? agrícola, minera o artesanal para la venta? [Even if you didn’t work last week, did you do any activity for at least one hour… for a salary or payment? In your own business or company? For a family business (with or without payment)? In exchange for goods? As an apprentice or in a paid internship? For sales, excluding household items? In agricultural, mining, or craft work for sale?] |
| 374 | p274 | ¿Aunque no trabajó la semana pasada, %NOMBRE% tenía algún empleo, negocio u otra actividad del cual estuvo ausente temporalmente por licencia, permiso postnatal parental, huelga, enfermedad, vacaciones, suspensión temporal u otra razón? [Although %NAME% did not work last week, did they have any job, business, or other activity from which they were temporarily absent due to leave, parental leave, strike, illness, vacation, temporary suspension, or other reason?] |
| 375 | p275 | ¿%NOMBRE% buscó trabajo remunerado o realizó alguna gestión para iniciar una actividad por cuenta propia (negocio o empresa) en las últimas cuatro semanas? [Has %NAME% looked for paid work or taken any steps to start a self-employed activity (business or enterprise) in the last four weeks?] |
| 376 | p276 | (CONTESTAN P272=1 o P273=1 o P274=1) ¿Cuál es su ocupación u oficio? [What is your occupation or trade?] |
| 377 | p277 | (CONTESTAN P272=1 o P273=1 o P274=1) ¿Qué hace usted en su trabajo o negocio principal? [What do you do in your main job or business?] |
| 378 | p278 | (CONTESTAN P272=1 o P273=1 o P274=1) ¿A qué se dedica o qué hace el negocio, empresa o institución donde usted trabaja? [What does the business, company, or institution where you work do?] |
| 379 | p279 | (CONTESTAN P272=1 o P273=1 o P274=1) En su trabajo o negocio principal, ¿Usted trabaja como? [In your main job or business, do you work as...?] |
| 380 | p280 | En su trabajo principal, ¿tiene contrato de trabajo escrito? [In your main job, do you have a written work contract?] |
| 381 | p281 | ¿A qué sistema previsional de salud pertenece usted? [Which health insurance system are you affiliated with?] |
| 382 | p282 | [SOLO PARA PERSONAS CON PAREJA P81 = 1] ¿Cuál es el nivel educacional más alto alcanzado o nivel educacional actual de su pareja? [What is the highest level of education your partner has completed or is currently attending?] |
| 383 | p283 | [SOLO PARA PERSONAS CON PAREJA P81 = 1] En ese nivel educacional, ¿cuál fue el último curso que su pareja aprobó o que cursa actualmente? [In that educational level, what was the last grade your partner completed or is currently attending?] |
| 384 | p284 | ¿Cuál fue el ingreso total de su hogar en el último mes? [What was your household’s total income last month?] |
| 385 | p285 | ¿Podría decirme en cuál de estos tramos está el ingreso total mensual de su hogar? [Could you tell me in which of these ranges your household’s monthly income falls?] |
| 386 | p286 | ¿Cree usted poder entregar información respecto a educación, ocupación e ingresos de los miembros del hogar? [Do you think you can provide information about the education, occupation, and income of household members?] |
| 387 | p287 | ¿Quién entregará información respecto de los miembros del hogar? [Who will provide information about the household members?] |
| 388 | p288 | ¿Cuál es el nivel educacional más alto alcanzado o nivel educacional actual de %MIEMBRO%? [What is the highest level of education %MEMBER% has completed or is currently attending?] |
| 389 | p289 | ¿Cuál fue el último curso que aprobó o que cursa actualmente %MIEMBRO%? [What was the last grade %MEMBER% completed or is currently attending?] |
| 390 | p290 | ¿%MIEMBRO% trabajó al menos una hora la semana pasada, sin contar quehaceres del hogar? [Did %MEMBER% work at least one hour last week, not counting household chores?] |
| 391 | p291 | ¿%MIEMBRO% realizó alguna actividad por lo menos durante una hora la semana pasada? [Did %MEMBER% perform any activity for at least one hour last week?] |
| 392 | p292 | ¿%MIEMBRO% tenía algún empleo, negocio u otra actividad del cual estuvo ausente temporalmente? [Did %MEMBER% have any job, business, or other activity from which they were temporarily absent?] |
| 393 | p293 | ¿%MIEMBRO% buscó trabajo remunerado o hizo alguna gestión para iniciar una actividad por cuenta propia en las últimas cuatro semanas? [Did %MEMBER% seek paid work or take any steps to start a self-employed activity in the past four weeks?] |
| 394 | p294 | ¿Cuál es la ocupación u oficio de %MIEMBRO%? (Indique el nombre completo del empleo u ocupación principal, y facilite detalles, por ejemplo: recolector de frutas, profesor de escuela nivel secundario, enfermera titulada, conductor de bus, guardia de seguridad, gerente de una empresa, entre otros). [What is %MIEMBRO%'s occupation or job? (Please specify the full name of the main job or occupation and provide details, for example: fruit picker, secondary school teacher, registered nurse, bus driver, security guard, company manager, among others).] |
| 395 | p295 | ¿Qué hace %MIEMBRO% en su trabajo o negocio principal? (Facilite detalles, por ejemplo: recoger y transportar uvas, enseñar matemáticas, cuidar enfermos y administrar medicamentos, transportar pasajeros entre ciudades, vigilar y controlar entrada en una empresa, administrar una empresa de productos lácteos). [What does %MIEMBRO% do in their main job or business? (Provide details, e.g., harvest and transport grapes, teach mathematics, care for the sick and administer medications, transport passengers between cities, monitor and control entry to a company, manage a dairy product business).] |
| 396 | p296 | ¿A qué se dedica o qué hace el negocio, empresa o institución donde %MIEMBRO% trabaja? (Describa la actividad a que se dedica la empresa, negocio o institución en que la persona realiza su actividad u ocupación principal). [What does the business, company or institution where %MIEMBRO% works do? (Describe the main activity of the business or institution where the person performs their main job).] |
| 397 | p297 | En su trabajo o negocio principal, ¿%MIEMBRO% trabaja como...? [In their main job or business, does %MIEMBRO% work as...?] |
| 398 | p298 | En %MES PASADO%, ¿cuál fue el ingreso de %MIEMBRO% proveniente de su o sus trabajos, ocupación o actividad? [In %LAST MONTH%, what was %MIEMBRO%'s income from their job(s), occupation, or activity?] |
| 399 | p299 | En %MES%, ¿cuál fue el ingreso que recibió %MIEMBRO% por jubilación o pensión? [In %MONTH%, what income did %MIEMBRO% receive from retirement or pension?] |
| 400 | p300 | En %MES PASADO%, ¿cuál fue el ingreso total que recibió %MIEMBRO% por subsidios, bonos o aportes del Estado? [In %LAST MONTH%, what was the total income %MIEMBRO% received from state subsidies, bonuses or contributions?] |
| 401 | p301 | Los últimos 12 meses, ¿cuál fue el ingreso que recibió %MIEMBRO% por los siguientes subsidios, bonos o aportes del Estado? [In the last 12 months, what income did %MIEMBRO% receive from the following subsidies, bonuses or state contributions?] |
| 402 | p302 | En %MES PASADO%, ¿cuál fue el ingreso total que recibió %MIEMBRO% por arriendos urbanos, pensión de alimentos o dinero aportado por terceros ajenos al hogar? [In %LAST MONTH%, what was the total income %MIEMBRO% received from urban rentals, alimony or money contributed by third parties outside the household?] |
| 403 | p303 | ¿Cuál fue el ingreso total de su hogar en el último mes? [What was the total income of your household last month?] |
| 404 | p304 | ¿Podría decirme en cuál de estos tramos está el ingreso total mensual de su hogar? [Could you tell me which of these ranges your household's total monthly income falls into?] |
| 405 | p305 | ¿Diría que el ingreso mensual del hogar les permite pagar sin necesidad de créditos o préstamos? [Would you say your household's monthly income allows you to pay without needing loans or credit?] |

Appendix 2 – Structured Coding Matrix of Selected ENSEXX Survey Items: Variable Labels, Theoretical Mapping, and Justification

| **N°** | **VARIABLE** | **Label ( questionnaire Item)** | **Justification** | **Coder 1 vote TDF** | **Coder 2 vote TDF** |
| --- | --- | --- | --- | --- | --- |
| 1 | p1 | ¿Cuál es su sexo asignado al nacer? [What is your sex assigned at birth?] | Do not code. Category does not meet the objective. | Not applicable | Not applicable |
| 2 | p2 | ¿Es usted una persona intersex? [Are you an intersex person?] | Do not code. Category does not meet the objective. | Not applicable | Not applicable |
| 3 | p3 | ¿Cuál es el género con el que Usted se identifica? [What gender do you identify with?] | Do not code. Category does not meet the objective. | Not applicable | Not applicable |
| 4 | p4 | ¿Qué edad tiene? [How old are you?] | Do not code. Category does not meet the objective. | Not applicable | Not applicable |
| 5 | p5 | ¿Cuál es su nivel educacional más alto alcanzado o su nivel educacional actual? [What is your highest level of education attained or your current educational level?] | Do not code. Category does not meet the objective. | Not applicable | Not applicable |
| 6 | p7 | ¿Cuál es su estado conyugal o civil actual? [What is your current marital or civil status?] | Do not code. Category does not meet the objective. | Not applicable | Not applicable |
| 7 | p8 | ¿Cómo calificaría su calidad de vida? [How would you rate your quality of life?] | Do not code. Category does not meet the objective. | Not applicable | Not applicable |
| 8 | i_1_p9 | (Con la privacidad que tiene donde vive) Ahora le preguntaré acerca de cómo se s [Regarding the privacy where you live, I will now ask how you feel about it] | Do not code. Category does not meet the objective. | Not applicable | Not applicable |
| 9 | i_3_p9 | (Con su vida amorosa) Ahora le preguntaré acerca de cómo se [Regarding your love life, I will now ask how you feel about it] | Do not code. Category does not meet the objective. | Not applicable | Not applicable |
| 10 | i_4_p9 | (Con la cantidad de diversión que tiene en su vida) Ahora le preguntaré acerca d [Regarding the amount of fun you have in your life, I will now ask how you feel about it] | Do not code. Category does not meet the objective. | Not applicable | Not applicable |
| 11 | i_5_p9 | (Con su vida familiar) Ahora le preguntaré acerca de cómo se siente Usted en dis [Regarding your family life, I will now ask how you feel about it] | Do not code. Category does not meet the objective. | Not applicable | Not applicable |
| 12 | i_6_p9 | (Con su vida sexual) Ahora le preguntaré acerca de cómo se siente Usted en disti [Regarding your sex life, I will now ask how you feel about it] | Do not code. Category does not meet the objective. | Not applicable | Not applicable |
| 13 | p10 | En general Usted diría que su salud es... [In general, would you say your health is...] | Do not code. Category does not meet the objective. | Not applicable | Not applicable |
| 14 | p22 | ¿Me podría decir cuánto pesa aproximadamente actualmente? [Could you tell me approximately how much you currently weigh?] | Do not code. Category does not meet the objective. | Not applicable | Not applicable |
| 15 | p23 | ¿Me podría decir cuánto mide aproximadamente actualmente? [Could you tell me approximately how tall you are currently?] | Do not code. Category does not meet the objective. | Not applicable | Not applicable |
| 16 | i_1_p25 | (Cocaína) ¿Ha probado Ud. alguna de las siguientes sustancias alguna vez en su vida? [Have you ever tried any of the following substances? (Cocaine)] | Do not code. Category does not meet the objective. | Not applicable | Context and resources |
| 17 | i_1_p26 | (Cocaína) ¿Cuándo fue la última vez que consumió? [When was the last time you used it? (Cocaine)] | Do not code. Category does not meet the objective. | Not applicable | Context and resources |
| 18 | i_2_p25 | (Marihuana) ¿Ha probado Ud. alguna de las siguientes sustancias alguna vez en su vida? [Have you ever tried any of the following substances? (Marijuana)] | Do not code. Category does not meet the objective. | Not applicable | Context and resources |
| 19 | i_2_p26 | (Marihuana) ¿Cuándo fue la última vez que consumió? [When was the last time you used it? (Marijuana)] | Do not code. Category does not meet the objective. | Not applicable | Context and resources |
| 20 | i_3_p25 | (Drogas inyectables) ¿Ha probado Ud. alguna de las siguientes sustancias alguna vez en su vida? [Have you ever tried any of the following substances? (Injectable drugs)] | Do not code. Category does not meet the objective. | Not applicable | Context and resources |
| 21 | i_3_p26 | (Drogas inyectables) ¿Cuándo fue la última vez que consumió? [When was the last time you used it? (Injectable drugs)] | Do not code. Category does not meet the objective. | Not applicable | Context and resources |
| 22 | i_4_p25 | (Tranquilizantes, ansiolíticos o antidepresivo) ¿Ha probado Ud. alguna de las siguientes sustancias alguna vez en su vida? [Have you ever tried any of the following substances? (Tranquilizers, anxiolytics or antidepressants)] | Do not code. Category does not meet the objective. | Not applicable | Context and resources |
| 23 | i_4_p26 | (Tranquilizantes, ansiolíticos o antidepresivo) ¿Cuándo fue la última vez que consumió? [When was the last time you used it? (Tranquilizers, anxiolytics or antidepressants)] | Do not code. Category does not meet the objective. | Not applicable | Context and resources |
| 24 | i_5_p25 | (Alcohol) ¿Ha probado Ud. alguna de las siguientes sustancias alguna vez en su vida? [Have you ever tried any of the following substances? (Alcohol)] | Do not code. Category does not meet the objective. | Not applicable | Context and resources |
| 25 | i_5_p26 | ¿Cuándo fue la última vez que consumió? [When was the last time you used it? (Alcohol)] | Do not code. Category does not meet the objective. | Not applicable | Context and resources |
| 26 | i_6_p25 | (Alucinógenos, hongos) ¿Ha probado Ud. alguna de las siguientes sustancias alguna vez en su vida? [Have you ever tried any of the following substances? (Hallucinogens, mushrooms)] | Do not code. Category does not meet the objective. | Not applicable | Context and resources |
| 27 | i_6_p26 | (Alucinógenos, hongos) ¿Cuándo fue la última vez que consumió? [When was the last time you used it? (Hallucinogens, mushrooms)] | Do not code. Category does not meet the objective. | Not applicable | Context and resources |
| 28 | i_7_p25 | (Poppers) ¿Ha probado Ud. alguna de las siguientes sustancias alguna vez en su vida? [Have you ever tried any of the following substances? (Poppers)] | Do not code. Category does not meet the objective. | Not applicable | Context and resources |
| 29 | i_7_p26 | (Poppers) ¿Cuándo fue la última vez que consumió? [When was the last time you used it? (Poppers)] | Do not code. Category does not meet the objective. | Not applicable | Context and resources |
| 30 | i_1_p33 | (Usar preservativos o condón disminuye el placer de las mujeres.) ¿Qué tan de acuerdo está con esta afirmación? [Using condoms decreases women's pleasure. How much do you agree with this statement?] | Investigates perceived barriers related to the impact of condom use on female sexual pleasure. | Beliefs about consequences | Beliefs about consequences |
| 31 | i_2_p33 | (Usar preservativos o condón disminuye el placer de los hombres.) ¿Qué tan de acuerdo está con esta afirmación? [Using condoms decreases men's pleasure. How much do you agree with this statement?] | Evaluates how condom use can be perceived as a barrier in male sexual experience. | Beliefs about consequences | Beliefs about consequences |
| 32 | i_3_p33 | (Es necesario ocupar preservativo o condón incluso si se tiene pareja estable) [It is necessary to use condoms even if you have a stable partner.] | Measures knowledge about the relevance of condom use in STI prevention, even among stable couples. | Knowledge | ~~Social Role & Identity~~ |
| 33 | i_4_p33 | (Usar preservativo o condón estimula el juego sexual.) [Using condoms enhances sexual play.] | Explores beliefs linking condom use with more positive sexual experiences. | Beliefs about consequences | Beliefs about consequences |
| 34 | i_5_p33 | (Los preservativos o condones son demasiado caros para usarlos regularmente) [Condoms are too expensive to use regularly.] | Analyzes economic barriers related to access and regular use of condoms. | Environmental context and resources | Context and resources |
| 35 | p34 | Cuando usted era niño/a, ¿En su familia se conversaban temas sexuales? [When you were a child, did your family talk about sexual topics?] | Examines the influence of early family socialization on sexuality and preventive practices. | Social influences | Social influences |
| 36 | p35 | (CONTESTAN P34=2,3) ¿Y con qué frecuencia usted participaba cuando se conversaban temas sexuales? [(If answered P34=2 or 3) How often did you participate in those conversations about sexual topics?] | Investigates the level of participation in family discussions related to sexuality and prevention. | Social influences | Social influences |
| 37 | t_p36_1 | En su escuela, cuándo usted era estudiante, ¿se impartía educación sexual en Enseñanza básica? [In your school, when you were a student, was sexual education provided in primary school?] | Evaluates the availability of formal sex education during primary education. | Environmental context and resources | Context and resources |
| 38 | t_p36_2 | En su escuela, cuándo usted era estudiante, ¿se impartía educación sexual en Enseñanza media? [In your school, when you were a student, was sexual education provided in secondary school?] | Investigates the continuity of sex education in higher educational levels. | Environmental context and resources | Context and resources |
| 39 | p37 | Y respecto de esa educación sexual, en general, usted cree que... [And regarding that sexual education, in general, you think that...] | Explores perceptions of the quality and impact of received sex education. | ~~Beliefs about consequences~~ | ~~Beliefs about capacity~~ |
| 40 | p38 | ¿Cómo evaluaría EN GENERAL la FORMACIÓN EN SEXUALIDAD que recibió en su colegio o escuela? [How would you rate, IN GENERAL, the SEXUALITY EDUCATION you received in your school?] | Evaluates general perceptions about sexuality education, without direct relation to specific behaviors. | ~~Beliefs about consequences~~ | ~~Beliefs about capacity~~ |
| 41 | i_2_p39 | (Métodos para prevenir infecciones de transmisión sexual) ¿Qué nota le pondría a...? [Methods to prevent sexually transmitted infections: What grade would you give it?] | Measures knowledge level about STI preventive methods. | Knowledge | Knowledge |
| 42 | i_1_p40_o1 | Cuando usted era adolescente, a raíz de alguna duda sobre los siguientes temas, ¿A quién recurrió para resolverlas? Relaciones sexuales [When you were a teenager, if you had doubts about the following topics, who did you turn to? Sexual relations] | Identifies support networks available to resolve doubts about sexual relationships. | Environmental context and resources | Context and resources |
| 43 | i_3_p40_o1 | Cuando usted era adolescente, a raíz de alguna duda sobre los siguientes temas, ¿A quién recurrió para resolverlas? (Métodos preventivos de infecciones de transmisión sexual, como el VIH) [When you were a teenager, if you had doubts about the following topics, who did you turn to? (Preventive methods for sexually transmitted infections, such as HIV)] | Evaluates access to specific information about STI preventive methods. | Environmental context and resources | Context and resources |
| 44 | i_4_p41 | (Que enseñe a poner un preservativo o condón) [That teaches how to put on a condom] | Do not code. Category does not meet the objective. | Not applicable | Not applicable |
| 45 | p49 | ¿A qué edad tuvo su primera relación sexual voluntaria? (SI NO SABE LA EDAD EXACTA, SEÑALE EDAD APROXIMADA. ANOTAR LA EDAD DE LA PRIMERA RELACIÓN SEXUAL VOLUNTARIA) [At what age did you have your first voluntary sexual intercourse? (If you don't know the exact age, indicate an approximate age)] | Do not code. Category does not meet the objective. | Not applicable | Not applicable |
| 46 | p55 | Antes de su primera relación sexual, ¿Usted y esa persona hablaron de cómo evitar una ITS…? [Before your first sexual intercourse, did you and that person talk about how to avoid STIs...?] | Explores partner communication about STIs before first sexual intercourse. | Social influences | Social influences |
| 47 | p56 | En esa primera relación sexual, ¿Ustedes usaron algún método anticonceptivo? [In that first sexual intercourse, did you use any contraceptive method?] | Measures adoption of preventive practices in the first sexual intercourse. | Behavioural regulation | ~~Intentions~~ |
| 48 | p57 | ¿Cuál o cuáles métodos anticonceptivos usaron en esa primera relación sexual? MARQUE TODAS LAS QUE CORRESPONDA. [Which contraceptive methods did you use in that first sexual encounter? MARK ALL THAT APPLY.] | Records which specific contraceptive methods were used in the first sexual intercourse. | Behavioural regulation | ~~Intentions~~ |
| 49 | p58 | ¿Cuál fue el principal motivo por el cual usaron preservativo o condón? [What was the main reason for using a condom?] | Explores motivations for condom use in a preventive context. | ~~Reinforcement~~ | Goals |
| 50 | p59 | ¿Dónde obtuvo el método o los métodos mencionados anteriormente? [Where did you obtain the method(s) mentioned above?] | Investigates access to contraceptive methods within the social environment. | Environmental context and resources | Context and resources |
| 51 | p60 | ¿En qué lugar tuvo su primera relación sexual? [Where did you have your first sexual intercourse?] | Do not code. Category does not meet the objective. | Not applicable | Context and resources |
| 52 | p69 | En el transcurso de toda su vida, ¿podría indicar con cuántas personas ha tenido relaciones sexuales? [...] [Over your lifetime, how many people have you had sexual intercourse with? Approximate number.] | Do not code. Category does not meet the objective. | Not applicable | Not applicable |
| 53 | p73 | (CONTESTAN P71>0) En las relaciones con esas parejas sexuales del último año, ¿con qué frecuencia usted usaba condón o preservativo? [In relationships with those sexual partners in the past year, how often did you use a condom?] | Measures frequency of condom use in recent sexual relationships. | Behavioural regulation | ~~Intentions~~ |
| 54 | p89 | Y en la primera relación sexual que volvió a tener con (NOMBRE PAREJA SEPARACIÓN) después de la separación ¿usaron condón o preservativo? [In the first sexual intercourse after reuniting with (PARTNER'S NAME) post-separation, did you use a condom?] | Assesses condom use following significant changes in civil or emotional status. | Behavioural regulation | ~~Intentions~~ |
| 55 | p103 | (CONTESTAN SI P74 >0) Justo antes o al momento de las relaciones sexuales que ha tenido en el último mes, ¿usted consumió alguna de las siguientes sustancias? [Just before or during sexual intercourse in the last month, did you consume any of the following substances?] | Analyzes how substance use affects sexual decision-making. | ~~Emotion~~ | Reinforcement |
| 56 | p104 | (CONTESTAN SI P74 >0) Y alguna de las personas con la que usted estaba, ¿había consumido alguna de las siguientes sustancias? (MÚLTIPLE) [And any of the people you were with, had they consumed any of the following substances? (MULTIPLE)] | Explores how partner decisions influence the sexual context. | ~~Emotion~~ | Social influences |
| 57 | p119 | En esa última relación sexual, ¿Ustedes usaron alguno método anticonceptivo? [In that last sexual encounter, did you use any contraceptive method?] | Measures contraceptive use in recent sexual encounters. | Behavioural regulation | ~~Intentions~~ |
| 58 | p120 | ¿Cuál o cuáles métodos anticonceptivos utilizaron? MARQUE TODAS LAS QUE CORRESPONDAN [Which contraceptive methods did you use? MARK ALL THAT APPLY] | Records preventive methods used in recent sexual relationships. | Behavioural regulation | ~~Intentions~~ |
| 59 | p121 | (CONTESTAN P120=1,2) ¿Por qué motivo usaron preservativo o condón? [Why did you use a condom?] | Explores specific reasons behind condom use. | ~~Reinforcement~~ | Goals |
| 60 | p122 | ¿Dónde obtuvo el método o los métodos mencionados anteriormente? [Where did you obtain the method(s) mentioned above?] | Investigates access routes to preventive methods. | Environmental context and resources | Context and resources |
| 61 | p123 | (CONTESTAN SI P119=2) ¿Por qué razón no usó ningún método anticonceptivo? [Why didn’t you use any contraceptive method?] | Identifies perceived barriers to contraceptive use. | Beliefs about capabilities | ~~Goals~~ |
| 62 | p133 | Durante su vida, usted se ha sentido atraído/a sexualmente por… (ENCUESTADOR: SOLO PIDA NÚMERO DE RESPUESTA) [Throughout your life, who have you felt sexually attracted to...? (INTERVIEWER: ONLY REQUEST RESPONSE NUMBER)] | Do not code. Category does not meet the objective. | Not applicable | Not applicable |
| 63 | p134 | Usted actualmente se identifica cómo: [You currently identify as:] | Do not code. Category does not meet the objective. | Not applicable | Not applicable |
| 64 | p151 | (RESPONDE SEXO = MUJER) ¿Alguna vez ha ido a una consulta con un profesional de la salud por temas propios de ginecología, pubertad, sexualidad...? [For women: Have you ever consulted a health professional for personal topics such as gynecology, puberty, sexuality...?] | Investigates access to medical services related to sexuality (women). | Environmental context and resources | Context and resources |
| 65 | p152 | (RESPONDE SEXO = HOMBRE) ¿Alguna vez en su vida ha ido a una consulta o donde algún profesional de la salud para tratar asuntos médicos PROPIOS relacionados con la urología, pubertad, sexualidad, métodos preventivos del embarazo o infecciones de transmisión sexual? [MEN ONLY: Have you ever gone to a medical consultation or seen a health professional for PERSONAL medical issues related to urology, puberty, sexuality, contraceptive methods, or sexually transmitted infections?] | Investigates access to medical services related to sexuality (men). | Environmental context and resources | Context and resources |
| 66 | p154 | ¿Por qué motivo fue esa primera vez? (MARQUE TODAS LAS QUE CONSIDERE) [What was the reason for that first visit? (CHECK ALL THAT APPLY)] | Explores motivations for attending the first medical consultation. | ~~Environmental context and resources~~ | Goals |
| 67 | p155 | ¿A qué lugar fue? (LEA ALTERNATIVAS. CIRCULE SOLO UNO) [Where did you go? (READ OPTIONS. CIRCLE ONLY ONE)] | Assesses physical access to health services during the first consultation. | Environmental context and resources | Context and resources |
| 68 | p202 | ¿Alguna vez en su vida, un doctor o médico le ha dicho que tiene o que padece de...? [Has a doctor ever told you that you have or suffer from...?] | Do not code. Category does not meet the objective. | Not applicable | Not applicable |
| 69 | p203 | ¿A qué edad se lo diagnosticaron por última vez? [How old were you the last time you were diagnosed?] | Do not code. Category does not meet the objective. | Not applicable | Not applicable |
| 70 | p206 | ¿Cómo descubrió que tenía alguna de esas infecciones? Si ha tenido más de una vez una infección, piense en la última. [How did you discover that you had any of those infections? If you’ve had more than one, refer to the most recent.] | Explores how external circumstances, such as access to resources or services, influence STI discovery. | Environmental context and resources | Context and resources |
| 71 | p207 | La última vez que fue diagnosticado de alguna infección de transmisión sexual, ¿se lo informó a su o sus parejas sexuales? [The last time you were diagnosed with an STI, did you inform your partner(s)?] | Measures communication about diagnoses with sexual partners. | ~~Social influences~~ | Behavioural regulation |
| 72 | p208 | [RESPONDEN P202.10 = 2] Por cualquier razón, ¿Se ha hecho el examen del VIH o Sida en los últimos 12 meses? [[ONLY IF P202.10 = 2] For any reason, have you been tested for HIV/AIDS in the past 12 months?] | Assesses uptake of HIV preventive testing in recent months. | ~~Beliefs about consequences~~ | Behavioural regulation |
| 73 | p210 | [SOLO PARA P202.10 = 1 & P208 = 1] ¿Por qué razón se hizo el examen del VIH o Sida? [[ONLY IF P202.10 = 1 & P208 = 1] What was the reason for getting the HIV/AIDS test?] | Identifies motivations for undergoing HIV testing. | Beliefs about consequences | ~~Goals~~ |
| 74 | p211 | [SOLO PARA P202.10 = 2 & P208 = 2] ¿Por qué razón no se ha hecho el examen del VIH o Sida en los últimos 12 meses? MARCAR TODAS LAS QUE CORRESPONDAN [[ONLY IF P202.10 = 2 & P208 = 2] Why haven’t you been tested for HIV/AIDS in the last 12 months? CHECK ALL THAT APPLY] | Explores perceived barriers to HIV testing. | Beliefs about consequences | ~~Goals~~ |
| 75 | i_1_p212 | Ahora le voy a hacer unas preguntas sobre VIH o Sida. Cree usted qué…¿Puede reducirse el riesgo de transmisión del VIH manteniendo relaciones sexuales con una única pareja fiel y sin VIH o Sida? [Can the risk of HIV transmission be reduced by having sex with only one faithful, uninfected partner?] | Measures general knowledge about HIV preventive measures. | Knowledge | ~~Beliefs about consequences~~ |
| 76 | i_2_p212 | ¿Puede reducirse el riesgo de transmisión del VIH usando preservativo o condón cada vez que se mantienen relaciones sexuales? [Can using a condom every time reduce the risk of HIV transmission?] | Measures general knowledge about HIV preventive measures. | Knowledge | ~~Beliefs about consequences~~ |
| 77 | i_3_p212 | ¿Puede una persona de aspecto saludable tener VIH? [Can a person who looks healthy have HIV?] | Measures general knowledge about HIV preventive measures. | Knowledge | ~~Beliefs about consequences~~ |
| 78 | i_4_p212 | ¿Se puede adquirir el VIH por picaduras de mosquito? [Can HIV be transmitted through mosquito bites?] | Measures general knowledge about HIV preventive measures. | Knowledge | ~~Beliefs about consequences~~ |
| 79 | i_5_p212 | ¿Se puede adquirir el VIH por compartir alimentos con una persona con VIH/Sida? [Can HIV be transmitted by sharing food with someone with HIV/AIDS?] | Measures general knowledge about HIV preventive measures. | Knowledge | ~~Beliefs about consequences~~ |
| 80 | i_6_p212 | ¿Se puede transmitir el VIH de la madre al niño/a en el embarazo, parto o durante la lactancia? [Can HIV be transmitted from mother to child during pregnancy, delivery, or breastfeeding?] | Measures general knowledge about HIV preventive measures. | Knowledge | ~~Beliefs about consequences~~ |
| 81 | p213 | ¿Conoce usted la medida “profilaxis de preexposición” o PREP como alternativa de prevención del VIH/Sida? [Do you know about pre-exposure prophylaxis (PrEP) as an HIV prevention method?] | Assesses knowledge about PrEP as an advanced preventive method. | Knowledge | Knowledge |
| 82 | p263 | ¿Cuál es su nacionalidad? [What is your nationality?] | Do not code. Category does not meet the objective. | Not applicable | Not applicable |
| 83 | p265 | Cuándo usted nació, ¿En qué país vivía su madre? [When you were born, in which country was your mother living?] | Do not code. Category does not meet the objective. | Not applicable | Not applicable |
| 84 | p267 | En Chile, la ley reconoce diez pueblos indígenas, ¿pertenece Usted o es descendiente de alguno de ellos? [In Chile, ten indigenous peoples are legally recognized. Do you belong to or are you a descendant of any of them?] | Do not code. Category does not meet the objective. | Not applicable | Not applicable |
| 85 | p268 | ¿Cuál es su religión o credo? [What is your religion or belief system?] | Do not code. Category does not meet the objective. | Not applicable | Not applicable |
| 86 | p269 | ¿Usted se definiría como una persona…? (LEA ALTERNATIVAS) [Would you define yourself as a person…? (READ ALTERNATIVES)] | Do not code. Category does not meet the objective. | Not applicable | Not applicable |
| 87 | p270 | En general, la gente suele situarse en posiciones políticas más cercanas a la izquierda, al centro o a la derecha. En una escala de 1 a 10, donde 1 es izquierda y 10 es derecha, ¿En qué lugar se ubicaría usted? Elija el número que quiera según se inclina más hacia uno u otro lado. [In general, people place themselves on the political spectrum from left to right. On a scale from 1 to 10, where 1 is left and 10 is right, where would you place yourself? Choose the number that best reflects your inclination.] | Do not code. Category does not meet the objective. | Not applicable | Not applicable |
| 88 | p271 | ¿Cuántos dormitorios, de uso exclusivo para dormir, tiene su vivienda? (INDICAR EL NUMERO DE PIEZAS) [How many bedrooms, used exclusively for sleeping, are in your household? (INDICATE THE NUMBER OF ROOMS)] | Do not code. Category does not meet the objective. | Not applicable | Not applicable |
| 89 | p272 | La semana pasada, ¿usted trabajó al menos una hora, sin considerar los quehaceres del hogar? [Last week, did you work at least one hour, not including household chores?] | Do not code. Category does not meet the objective. | Not applicable | Not applicable |
| 90 | p273 | Aunque no trabajó la semana pasada, ¿usted realizó alguna actividad por lo menos durante una hora? …por un salario o remuneración? ¿en su empresa o negocio? ¿para la empresa o negocio de un familiar (con o sin remuneración)? ¿por pago en especies? ¿como aprendiz o realizando una práctica remunerada? ¿de venta, sin incluir bienes del hogar? ¿agrícola, minera o artesanal para la venta? [Even if you did not work last week, did you carry out any activity for at least one hour? …for a salary or remuneration? in your own business or company? in a relative’s business (with or without pay)? in exchange for goods? as an apprentice or in a paid internship? sales (excluding household goods)? agricultural, mining, or artisan work for sale?] | Do not code. Category does not meet the objective. | Not applicable | Not applicable |
| 91 | p274 | Aunque no trabajó la semana pasada, ¿%NOMBRE% tenía algún empleo, negocio u otra actividad del cual estuvo ausente temporalmente por licencia, permiso postnatal parental, huelga, enfermedad, vacaciones, suspensión temporal u otra razón? [Even if you did not work last week, did %NAME% have a job, business or other activity from which they were temporarily absent due to leave, parental leave, strike, illness, vacation, suspension or another reason?] | Do not code. Category does not meet the objective. | Not applicable | Not applicable |
| 92 | p275 | ¿%NOMBRE% buscó trabajo remunerado o realizó alguna gestión para iniciar una actividad por cuenta propia (negocio o empresa) en las últimas cuatro semanas? [%NAME%: In the last four weeks, did you look for paid work or take any steps to start a self-employment activity (business or enterprise)?] | Do not code. Category does not meet the objective. | Not applicable | Not applicable |
| 93 | p276 | (CONTESTAN P272=1 o P273=1 o P274=1) Ahora quisiera preguntarle acerca de su trabajo o negocio principal. ¿Cuál es su ocupación u oficio? (Indique el nombre completo del empleo u ocupación principal, y facilite detalles…) [(IF P272=1 or P273=1 or P274=1) Now I would like to ask about your main job or business. What is your occupation or trade? (Provide the full name and details…)] | Do not code. Category does not meet the objective. | Not applicable | Not applicable |
| 94 | p277 | (CONTESTAN P272=1 o P273=1 o P274=1) ¿Qué hace usted en su trabajo o negocio principal? (Facilite detalles…) [(IF P272=1 or P273=1 or P274=1) What do you do in your main job or business? (Provide details…)] | Do not code. Category does not meet the objective. | Not applicable | Not applicable |
| 95 | p278 | (CONTESTAN P272=1 o P273=1 o P274=1) ¿A qué se dedica o qué hace el negocio, empresa o institución donde usted trabaja? (Describa la actividad…) [(IF P272=1 or P273=1 or P274=1) What does the business, company or institution where you work do? (Describe the activity…)] | Do not code. Category does not meet the objective. | Not applicable | Not applicable |
| 96 | p279 | (CONTESTAN P272=1 o P273=1 o P274=1) En su trabajo o negocio principal, ¿Usted trabaja como? [(IF P272=1 or P273=1 or P274=1) In your main job or business, do you work as…?] | Do not code. Category does not meet the objective. | Not applicable | Not applicable |
| 97 | p280 | En su trabajo principal, ¿tiene contrato de trabajo escrito? (Entrevistado, responder si P279=3;4;5;6;7) [In your main job, do you have a written employment contract? (Interviewer, respond if P279=3;4;5;6;7)] | Do not code. Category does not meet the objective. | Not applicable | Not applicable |
| 98 | p281 | ¿A qué sistema previsional de salud pertenece usted? [Which health insurance system are you affiliated with?] | Do not code. Category does not meet the objective. | Not applicable | Not applicable |
| 99 | p284 | Por favor, piense en ingreso total de su hogar en el MES PASADO, considerando el aporte de todos sus miembros y otros ingresos adicionales como rentas de propiedades, jubilaciones o pensiones. ¿Cuál fue el ingreso total de su hogar en el último mes? [Please think of your household’s total income LAST MONTH, including all contributions and additional income (rents, pensions, etc.). What was the total household income last month?] | Do not code. Category does not meet the objective. | Not applicable | Not applicable |
| 100 | p285 | (APLICAR SOLO EN CASO DE QUE NO RESPONDA P284) ¿Podría decirme en cuál de estos tramos está el ingreso total mensual de su hogar? (PREGUNTA FILTRADA SEGÚN MIEMBROS DEL HOGAR) [(ASK ONLY IF P284 IS UNANSWERED) Could you tell me in which of these brackets your total monthly household income fits? (QUESTION FILTERED BY NUMBER OF HOUSEHOLD MEMBERS)] | Do not code. Category does not meet the objective. | Not applicable | Not applicable |

Appendix Table S2.1–Classification of ENSSEX items into descriptive and injunctive (prescriptive) norms

| **Type of norm** | **ENSSEX item (code + text)** | **Example of interpretation** |
| --- | --- | --- |
| **Descriptive** | P73. In your sexual relationships over the past year, how often did you use condoms? | Captures actual condom use; when aggregated, reflects common practice in the population. |
| **Descriptive** | P119. In your last sexual encounter, did you use any contraceptive method?? | Provides a population-level indicator of protection at last sex; auxiliary for temporal coherence; not condom-specific. |
| **Descriptive** | P34. Have you discussed sexuality with your family? | Describes social practices in family contexts, reflecting what is typical within households. |
| **Injunctive** | i_3_p33. Is it necessary to use condoms even when having a stable partner? | Represents a normative expectation of what should be done, independent of actual practice. |
| **Injunctive** | i_1_p33 / i_2_p33. Condoms reduce women’s/men’s sexual pleasure. | Reflect beliefs about consequences that shape approval or disapproval of condom use. |
| **Injunctive** | P55. Did you discuss STI prevention with your partner before your first sexual relationship? | Illustrates a normative standard of socially approved behavior before initiating sex. |

Note: Descriptive norms were approximated using survey-weighted behavioral prevalence and were not entered as independent covariates. P73 was the primary outcome; P119 was used only as an auxiliary indicator for temporal coherence. Classification adapted from Cialdini et al. (1990) [23] on descriptive vs. injunctive norms.

Table S2.1 — Normative constructs (descriptive vs. injunctive). Following the normative conduct framework, we distinguished descriptive norms—population-level behavioral regularities indicating what people typically do—from injunctive (prescriptive) norms, which capture what is socially approved or expected. In ENSSEX, descriptive norms were approximated using survey-weighted behavioral prevalence (e.g., condom use in the past year, contraceptive use at last sex, family discussion about sexuality) and reported as contextual indicators rather than included as independent covariates (condom use at 12 months, P73, was our primary outcome; P119 served only as an auxiliary check). Conversely, injunctive norms (e.g., endorsing condom use even with a stable partner; pleasure-related beliefs; pre-sex prevention talk) were included as theory-driven explanatory variables in multivariable models. This operational split clarifies the complementary roles of what is commonly done versus what ought to be done, aligning with canonical distinctions in the literature and guiding our interpretation of normative influences on condom use and HIV/STI testing.

Appendix 3 –. Final Coding Matrix: Mapping of ENSEXX Survey Items to TDF, and COM-B Frameworks

| **N°** | **VARIABLE** | **Questionnaire item (original in Spanish with English translation)** | **Domain TDF AGREED** | **COM Sub-Contructs** | **COM-B** |
| --- | --- | --- | --- | --- | --- |
| 1 | i_1_p33 | Según lo que usted cree, ¿qué tan de acuerdo está con que usar preservativos o condón disminuye el placer de las mujeres? [According to what you believe, how much do you agree that using condoms reduces women's sexual pleasure?] | Beliefs about consequences | Reflective motivation | Motivation |
| 2 | i_2_p33 | Según lo que usted cree, ¿qué tan de acuerdo está con que usar preservativos o condón disminuye el placer de los hombres? [According to what you believe, how much do you agree that using condoms reduces men's sexual pleasure?] | Beliefs about consequences | Reflective motivation | Motivation |
| 3 | i_3_p33 | Según lo que usted cree, ¿es necesario ocupar preservativo o condón incluso si se tiene pareja estable? [According to what you believe, is it necessary to use condoms even when in a stable relationship?] | Knowledge | Psychological Capability | Capability |
| 4 | i_4_p33 | Según lo que usted cree, ¿usar preservativo o condón estimula el juego sexual? [According to what you believe, does using condoms enhance sexual play?] | Beliefs about consequences | Reflective motivation | Motivation |
| 5 | i_5_p33 | Según lo que usted cree, ¿los preservativos o condones son demasiado caros para usarlos regularmente? [According to what you believe, are condoms too expensive to use regularly?] | Environmental context and resources | Physical opportunity | Opportunity |
| 6 | p34 | Cuando usted era niño/a, ¿En su familia se conversaban temas sexuales? [When you were a child, did your family talk about sexual topics?] | Social influences | Social opportunity | Opportunity |
| 7 | p35 | ¿Y con qué frecuencia usted participaba cuando se conversaban temas sexuales? [And how often did you participate when sexual topics were discussed?] | Social influences | Social opportunity | Opportunity |
| 8 | t_p36_1 | En su escuela, cuándo usted era estudiante, ¿se impartía educación sexual en Enseñanza básica? [In your school, when you were a student, was sex education taught in primary school?] | Environmental context and resources | Physical opportunity | Opportunity |
| 9 | t_p36_2 | En su escuela, cuándo usted era estudiante, ¿se impartía educación sexual en Enseñanza media? [In your school, when you were a student, was sex education taught in secondary school?] | Environmental context and resources | Physical opportunity | Opportunity |
| 10 | p37 | Y respecto de esa educación sexual, en general, usted cree que… [And regarding that sex education, in general, do you think that...] | Beliefs about capacity | Reflective motivation | Motivation |
| 11 | p38 | ¿Cómo evaluaría en general la formación en sexualidad que recibió en su colegio o escuela? [How would you evaluate the sexuality education you received in school?] | Beliefs about capacity | Reflective motivation | Motivation |
| 12 | i_2_p39 | ¿Qué nota le podría ahora al conocimiento que usted tenía en los siguientes temas (Métodos para prevenir infecciones de transmisión sexual), cuando era adolescente? [How would you now grade the knowledge you had on the following topics (methods to prevent sexually transmitted infections), when you were an adolescent?] | Knowledge | Psychological Capability | Capability |
| 13 | i_1_p40_o1 | Cuando usted era adolescente, a raíz de alguna duda sobre los siguientes temas, ¿A quién recurrió para resolverlas? Relaciones sexuales [As an adolescent, if you had questions about sexual intercourse, who did you turn to for answers?] | Environmental context and resources | Physical opportunity | Motivation |
| 14 | i_3_p40_o1 | Cuando usted era adolescente, a raíz de alguna duda sobre los siguientes temas, ¿A quién recurrió para resolverlas? (Métodos preventivos de infecciones de transmisión sexual, como el VIH)  [As an adolescent, if you had questions about preventive methods for sexually transmitted infections like HIV, who did you turn to for answers?] | Environmental context and resources | Physical opportunity | Motivation |
| 15 | p55 | Antes de su primera relación sexual, ¿Usted y esa persona hablaron de cómo evitar una ITS…?  [Before your first sexual intercourse, did you and your partner talk about how to avoid STIs...?] | Social influences | Social opportunity | Motivation |
| 16 | p56 | En esa primera relación sexual, ¿Ustedes usaron algún método anticonceptivo? [In that first sexual intercourse, did you use any contraceptive method?] | Behavioural regulation | Psychological Capability | Capability |
| 17 | p57 | ¿Cuál o cuáles métodos anticonceptivos usaron en esa primera relación sexual? [Which contraceptive methods did you use in that first sexual intercourse?] | Behavioural regulation | Psychological Capability | Capability |
| 18 | p58 | ¿Cuál fue el principal motivo por el cual usaron preservativo o condón? [What was the main reason for using a condom in that first sexual intercourse?] | Goals | Reflective motivation | Motivation |
| 19 | p59 | ¿Dónde obtuvo el método o los métodos mencionados anteriormente? [Where did you get the contraceptive method(s) mentioned above?] | Environmental context and resources | Physical opportunity | Opportunity |
| 20 | p73 | En las relaciones con esas parejas sexuales del último año, ¿con qué frecuencia usted usaba condón o preservativo? [In your sexual relationships over the past year, how often did you use condoms?] | Behavioural regulation | Psychological Capability | Capability |
| 21 | p89 | Y en la primera relación sexual que volvió a tener con es persona después de la separación ¿usaron condón o preservativo? [In the first sexual encounter you had again with that person after the separation, did you use a condom?] | Behavioural regulation | Psychological Capability | Capability |
| 22 | p103 | Justo antes o al momento de las relaciones sexuales que ha tenido en el último mes, ¿usted consumió alguna de las siguientes sustancias? [Just before or during your sexual encounters in the last month, did you use any of the following substances?] | Reinforcement | Automatic motivation | Motivation |
| 23 | p104 | Y alguna de las personas con la que usted estaba, ¿había consumido alguna de las siguientes sustancias? [And did any of the people you were with use any of the following substances?] | Social influences | Social opportunity | Opportunity |
| 24 | p119 | En esa última relación sexual, ¿Ustedes usaron alguno método anticonceptivo? [In that last sexual encounter, did you use any contraceptive method?] | Behavioural regulation | Psychological Capability | Capability |
| 25 | p120 | ¿Cuál o cuáles métodos anticonceptivos utilizaron? [Which contraceptive methods did you use?] | Behavioural regulation | Psychological Capability | Capability |
| 26 | p121 | ¿Por qué motivo usaron preservativo o condón? [What was the reason for using a condom?] | Goals | Reflective motivation | Motivation |
| 27 | p122 | ¿Dónde obtuvo el método o los métodos mencionados anteriormente? [Where did you get the contraceptive method(s) mentioned above?] | Environmental context and resources | Physical opportunity | Opportunity |
| 28 | p123 | ¿Por qué razón no usó ningún método anticonceptivo? [Why did you not use any contraceptive method?] | Beliefs about capabilities | Reflective motivation | Motivation |
| 29 | p151 | ¿Alguna vez en su vida ha ido a una consulta o donde algún profesional de la salud para tratar asuntos médicos PROPIOS relacionados con la ginecología, pubertad, sexualidad, métodos preventivos del embarazo o infecciones de transmisión sexual? [Have you ever visited a health professional for personal medical concerns related to gynecology, puberty, sexuality, pregnancy prevention or sexually transmitted infections?] | Environmental context and resources | Physical opportunity | Opportunity |
| 30 | p152 | ¿Alguna vez en su vida ha ido a una consulta o donde algún profesional de la salud para tratar asuntos médicos PROPIOS relacionados con la urología, pubertad, sexualidad, métodos preventivos del embarazo o infecciones de transmisión sexual? [Have you ever visited a health professional for personal medical concerns related to urology, puberty, sexuality, pregnancy prevention or sexually transmitted infections?] | Environmental context and resources | Physical opportunity | Opportunity |
| 31 | p154 | ¿Por qué motivo fue esa primera vez? [What was the reason for that first visit?] | Goals | Reflective motivation | Motivation |
| 32 | p155 | ¿A qué lugar fue? [Where did you go?] | Environmental context and resources | Physical opportunity | Opportunity |
| 33 | p206 | ¿Cómo descubrió que tenía alguna de esas infecciones? Si ha tenido más de una vez una infección, piense en la última. [How did you find out you had one of these infections? If you have had more than one, think of the most recent.] | Environmental context and resources | Physical opportunity | Opportunity |
| 34 | p207 | La última vez que fue diagnosticado de alguna infección de transmisión sexual, ¿se lo informó a su o sus parejas sexuales? [The last time you were diagnosed with a sexually transmitted infection, did you inform your sexual partner(s)?] | Behavioural regulation | Psychological Capability | Capability |
| 35 | p208 | Por cualquier razón, ¿Se ha hecho el examen del VIH o Sida en los últimos 12 meses? [For any reason, have you had an HIV test in the last 12 months?] | Behavioural regulation | Psychological Capability | Capability |
| 36 | p210 | ¿Por qué razón se hizo el examen del VIH o Sida? [Why did you have an HIV test?] | Beliefs about consequences | Reflective motivation | Motivation |
| 37 | p211 | ¿Por qué razón no se ha hecho el examen del VIH o Sida en los últimos 12 meses? [Why have you not had an HIV test in the last 12 months?] | Beliefs about consequences | Reflective motivation | Motivation |
| 38 | i_1_p212 | ¿Puede reducirse el riesgo de transmisión del VIH manteniendo relaciones sexuales con una única pareja fiel y sin VIH o Sida? [Can the risk of HIV transmission be reduced by having sex with one faithful partner who does not have HIV/AIDS?] | Knowledge | Psychological Capability | Capability |
| 39 | i_2_p212 | ¿Puede reducirse el riesgo de transmisión del VIH usando preservativo o condón cada vez que se mantienen relaciones sexuales? [Can the risk of HIV transmission be reduced by using condoms every time you have sex?] | Knowledge | Psychological Capability | Capability |
| 40 | i_3_p212 | ¿Puede una persona de aspecto saludable tener VIH?  [Can a healthy-looking person have HIV?] | Knowledge | Psychological Capability | Capability |
| 41 | i_4_p212 | ¿Se puede adquirir el VIH por picaduras de mosquito? [Can HIV be transmitted through mosquito bites?] | Knowledge | Psychological Capability | Capability |
| 42 | i_5_p212 | ¿Se puede adquirir el VIH por compartir alimentos con una persona con VIH/Sida? [Can HIV be transmitted by sharing food with someone with HIV/AIDS?] | Knowledge | Psychological Capability | Capability |
| 43 | i_6_p212 | ¿Se puede transmitir el VIH de la madre al niño/a en el embarazo, parto o durante la lactancia? [Can HIV be transmitted from mother to child during pregnancy, childbirth or breastfeeding?] | Knowledge | Psychological Capability | Capability |
| 44 | p213 | ¿Conoce usted la medida “profilaxis de preexposición” o PREP como alternativa de prevención del VIH/Sida? [Do you know about “pre-exposure prophylaxis” or PrEP as an alternative for HIV/AIDS prevention?] | Knowledge | Psychological Capability | Capability |

| **TDF domain** | **Description** |
| --- | --- |
| Knowledge | An awareness of the existence of something |
| Skills | An ability or proficiency acquired through practice |
| Social/professional role and identity | A coherent set of behaviors and displayed personal qualities of an individual in a social or work setting |
| Beliefs about capabilities | Acceptance of the truth, reality, or validity about an ability, talent, or facility that a person can put to constructive use |
| Optimism | The confidence that things will happen for the best, or that desired goals will be attained |
| Beliefs about consequences | Acceptance of the truth, reality, or validity about outcomes of a behavior in a given situation |
| Reinforcement | Increasing the probability of a response by arranging a dependent relationship, or contingency, between the response and a given stimulus |
| Intentions | A conscious decision to perform a behavior or a resolve to act in a certain way |
| Goals | Mental representation of outcomes or end states that an individual wants to achieve |
| Memory, attention and decision processes | The ability to retain information, focus selectively on aspects of the environment, and choose between two or more alternatives |
| Environmental context and resources | Any circumstance of a person’s situation or environment that discourages or encourages the development of skills and abilities, independence, social competence, and adaptive behavior |
| Social influences | Those interpersonal processes that can cause an individual to change their thoughts, feelings, or behaviors |
| Emotion | A complex reaction pattern, involving experiential, behavioral, and physiological elements, by which the individual attempts to deal with a personally significant matter or event |
| Behavioral regulation | Anything aimed at managing or changing objectively observed or measured actions |

# Appendix 4 – Intercoder reliability calculation report

Data Overview:

The data used for the analysis is summarized in the following table:

|  | Coder 1 - Yes | Coder 1 - No | Total |
| --- | --- | --- | --- |
| Coder 2 - Yes | 90 | 0 | 90 |
| Coder 2 - No | 6 | 309 | 315 |
| Total | 96 | 309 | 405 |

Calculation of Observed Agreement (Po):

The observed agreement (Po) is the proportion of cases where both coders agreed. The formula used is:
Po = (a + d) / N
Where:
- a = 90 (cases where both coders said "Yes")
- d = 309 (cases where both coders said "No")
- N = 405 (total cases)

The observed agreement is:
Po = 90 / 309 = 0.985
This indicates that the coders agreed in 98.5% of the cases.

Calculation of Expected Agreement (Pe):

The expected agreement (Pe) accounts for the agreement that could occur by chance. The formula used is:
Pe = [(a+b) * (a+c) + (c+d) * (b+d)] / N^2
Where:
- (a + b) = 90 (coder 1 - Yes)
- (a + c) = 96 (coder 2 - Yes)
- (c + d) = 305 (coder 2 - No)
- (b + d) = 309 (coder 1 - No)

The expected agreement is:
Pe = 0.646

Calculation of Cohen's Kappa (K):

Cohen's Kappa is calculated to determine the agreement beyond chance. The formula used is:
K = (Po - Pe) / (1 - Pe)
Substituting the calculated values:
K = (0.985 - 0.646) / (1-0.646) = 0.958

The interpretation of kappa values followed these ranges: < 0.00: Poor agreement, 0.00–0.20: Slight agreement, 0.21–0.40: Fair agreement, 0.41–0.60: Moderate agreement, 0.61–0.80: Substantial agreement, 0.81–1.00: Almost perfect agreement.

Interpretation:

The Kappa value of 0.958 suggests an almost perfect agreement between the coders. According to the standard interpretation of Kappa values, this level of agreement indicates that the coders' decisions are highly reliable and consistent.

Appendix S5. Model diagnostics (AUC, Hosmer–Lemeshow, spline plots, sensitivity to missingness).

S4. Model diagnostics (AUC, Hosmer–Lemeshow, spline plots, sensitivity to missingness).

**Condom use**

Model discrimination was moderate (AUC ≈ 0.65), and the Hosmer–Lemeshow test indicated no evidence of lack of fit (p ≈ 0.23).


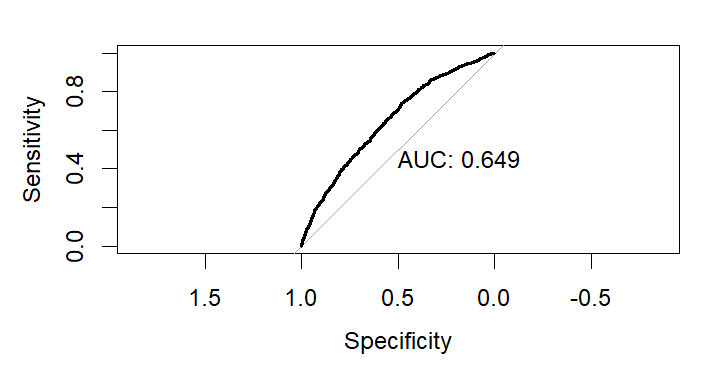


**HIV/STI testing in the past 12 months (design-weighted).**

The model showed good fit (Hosmer–Lemeshow p ≈ 0.56) and moderate discrimination (AUC ≈ 0.67–0.68).


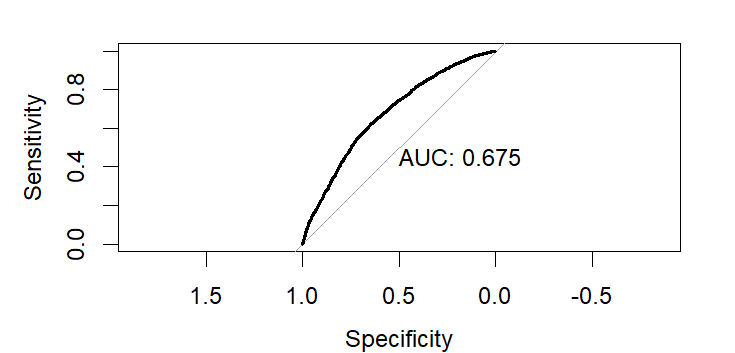


Appendix Table S5a and S5b

Supplementary predicted probabilities

Age-specific predicted probabilities, aligned with Figures 1–2, are provided for transparency and reproducibility:

Table S5a. Predicted probability of consistent condom use at the same ages, by sex (weighted; marginal effects with 95% CIs).

| Table S5a. Predicted probability of consistent condom use at the same ages, by sex (weighted; marginal effects with 95% CIs). | | | |
| --- | --- | --- | --- |
| Age | Sex | Predicted probability | 95%% CI |
| 18 | Male | 50.7% | 40.4–61.0% |
| 18 | Female | 38.7% | 29.2–49.2% |
| 20 | Male | 46.9% | 38.4–55.7% |
| 20 | Female | 35.2% | 27.6–43.7% |
| 25 | Male | 38.0% | 31.4–45.0% |
| 25 | Female | 27.3% | 22.2–33.1% |
| 30 | Male | 30.5% | 23.0–39.3% |
| 30 | Female | 21.2% | 15.7–28.1% |
| 35 | Male | 25.3% | 16.4–36.8% |
| 35 | Female | 17.2% | 10.8–26.2% |
| 40 | Male | 22.8% | 14.2–34.7% |
| 40 | Female | 15.4% | 9.1–24.7% |
| 45 | Male | 24.0% | 16.2–34.0% |
| 45 | Female | 16.2% | 10.3–24.6% |
| 50 | Male | 29.8% | 10.4–60.8% |
| 50 | Female | 20.7% | 6.5–49.4% |
| 55 | Male | 40.5% | 3.7–92.3% |
| 55 | Female | 29.4% | 2.3–88.3% |
| 60 | Male | 54.3% | 1.0–99.3% |
| 60 | Female | 42.1% | 0.6–98.8% |

Table S5b. Predicted probability of HIV/STI testing at ages 18, 20, 25, 30, 35, 40, 45, 50, 55 and 60 (weighted; marginal effects with 95% CIs).

| Table S5b. Predicted probability of HIV/STI testing at ages 18, 20, 25, 30, 35, 40, 45, 50, 55 and 60 (weighted; marginal effects with 95% CIs). | | |
| --- | --- | --- |
| Age | Predicted probability | 95%% CI |
| 18 | 23.0% | 17.5–29.5% |
| 20 | 24.5% | 19.4–30.4% |
| 25 | 27.7% | 23.3–32.6% |
| 30 | 28.4% | 24.1–33.2% |
| 35 | 25.4% | 21.0–30.3% |
| 40 | 20.0% | 15.8–24.9% |
| 45 | 14.4% | 11.3–18.2% |
| 50 | 10.0% | 7.9–12.6% |
| 55 | 6.8% | 4.4–10.3% |
| 60 | 4.5% | 2.1–9.6% |


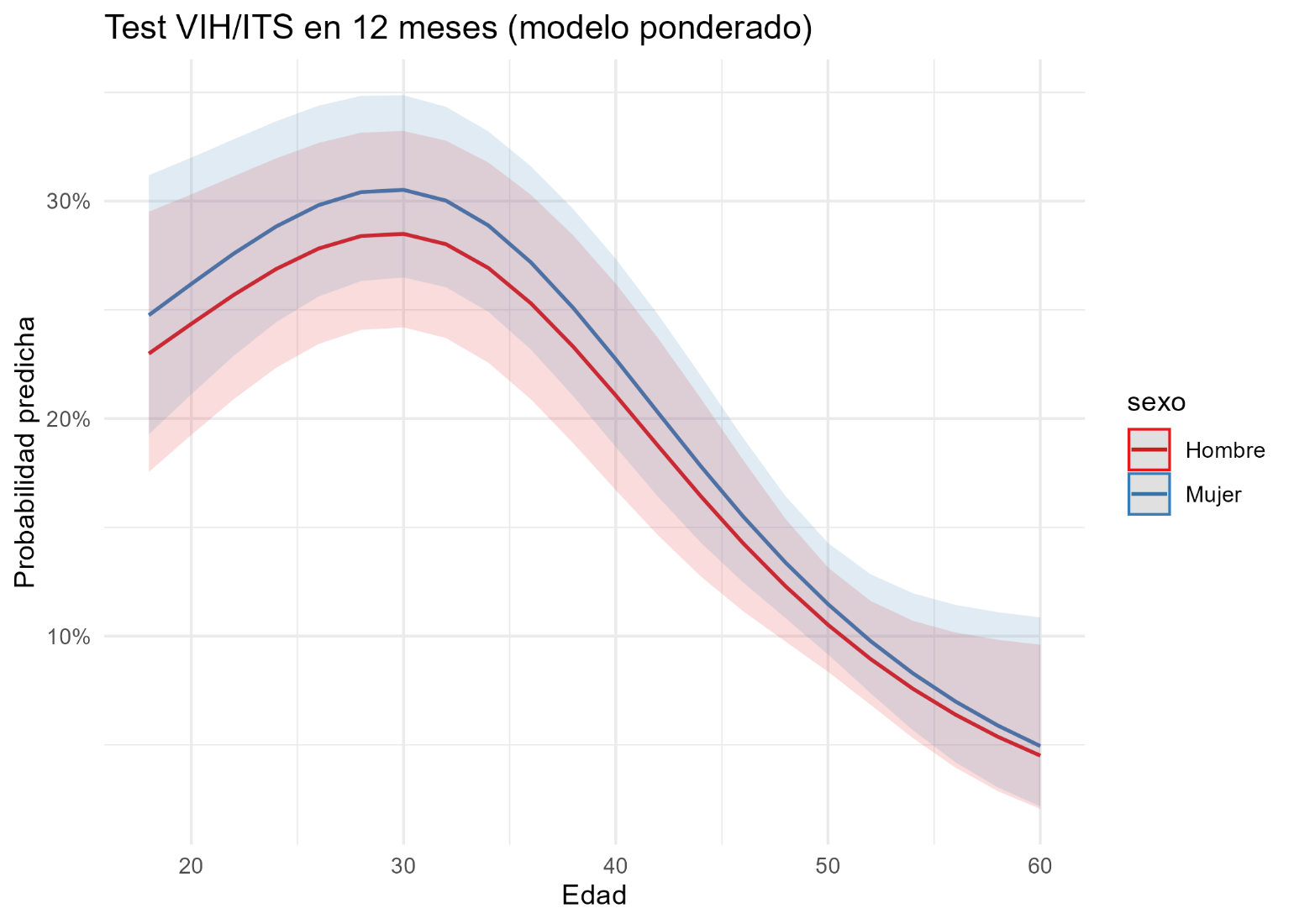


**Figure S5a. Predicted probability of HIV/STI testing within the last 12 months by age and sex (weighted model). Shaded areas represent 95% confidence intervals. Red lines = men; blue lines = women. The x-axis represents age (years), and the y-axis represents the predicted probability of having an HIV/STI test in the past year. (Original figure labels are in Spanish.)**


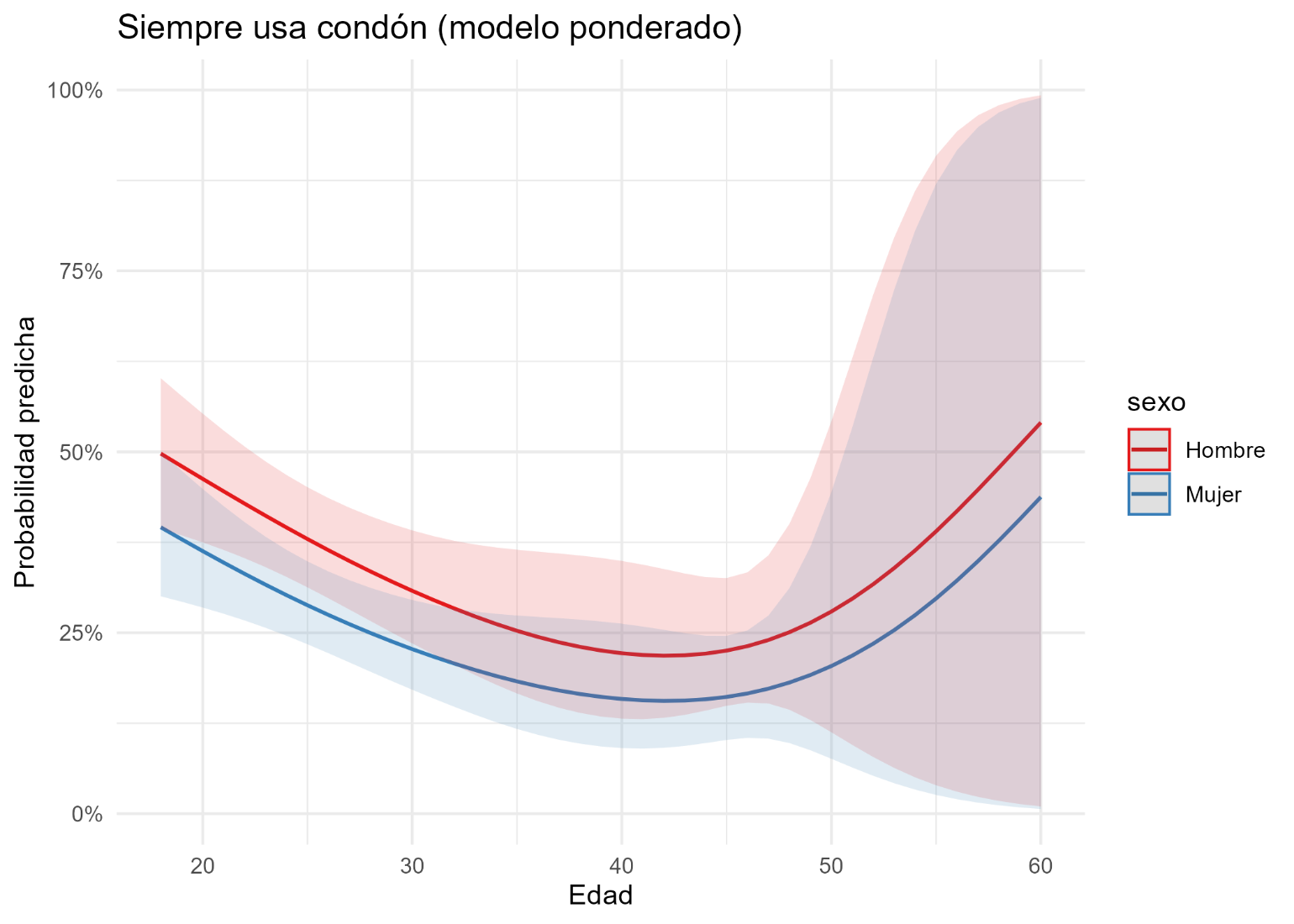


**Figure S5b. Predicted probability of consistent condom use by age and sex (weighted model). Shaded areas represent 95% confidence intervals. Red lines = men; blue lines = women. The x-axis represents age (years), and the y-axis represents the predicted probability of always using condoms during the past 12 months. (Original figure labels are in Spanish.)**


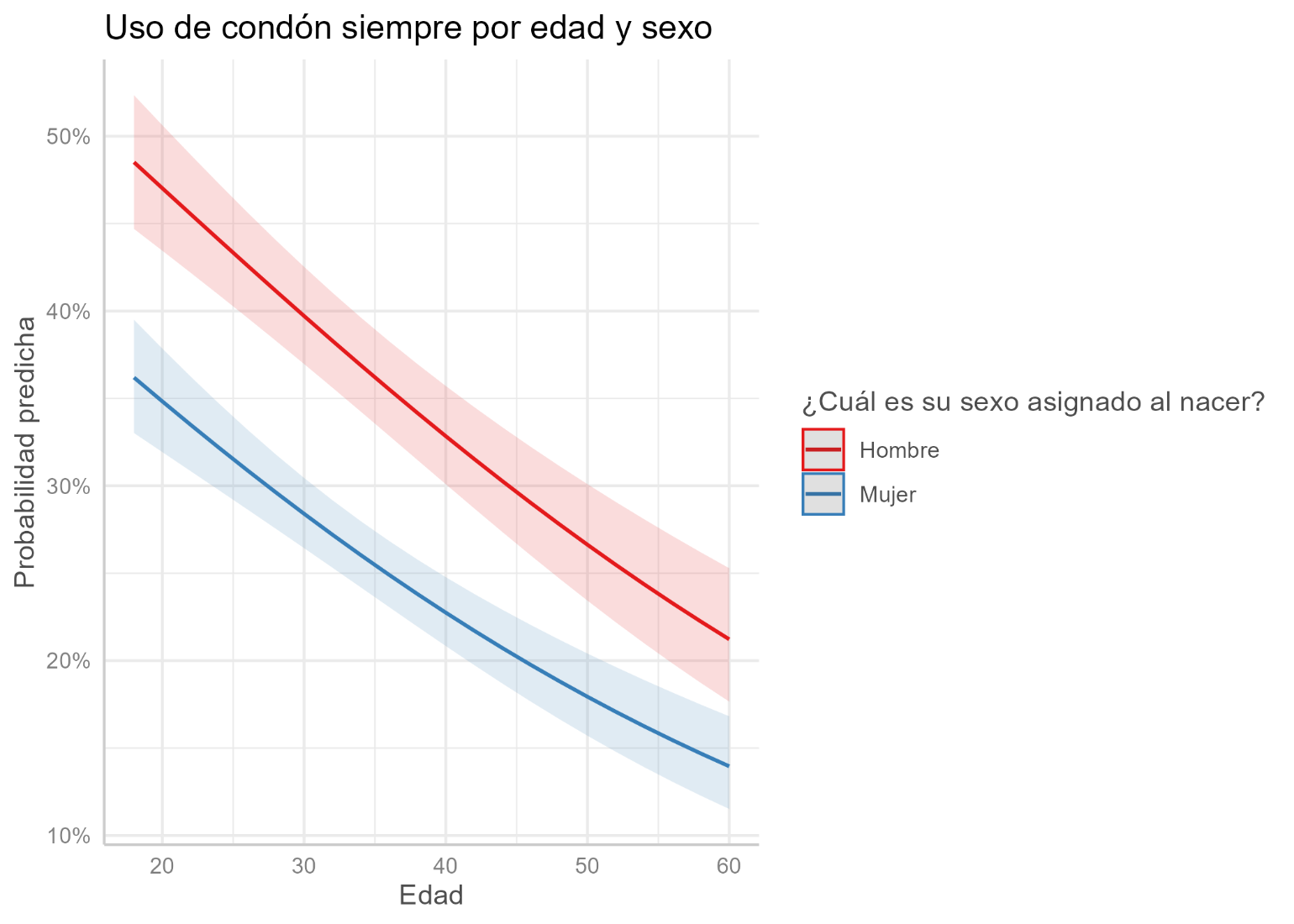


**Figure S5c. Predicted probability of consistent condom use by age and sex. Shaded areas represent 95% confidence intervals. Red lines = men; blue lines = women. The x-axis represents age (years), and the y-axis represents the predicted probability of always using condoms. (Original figure labels are in Spanish.)**


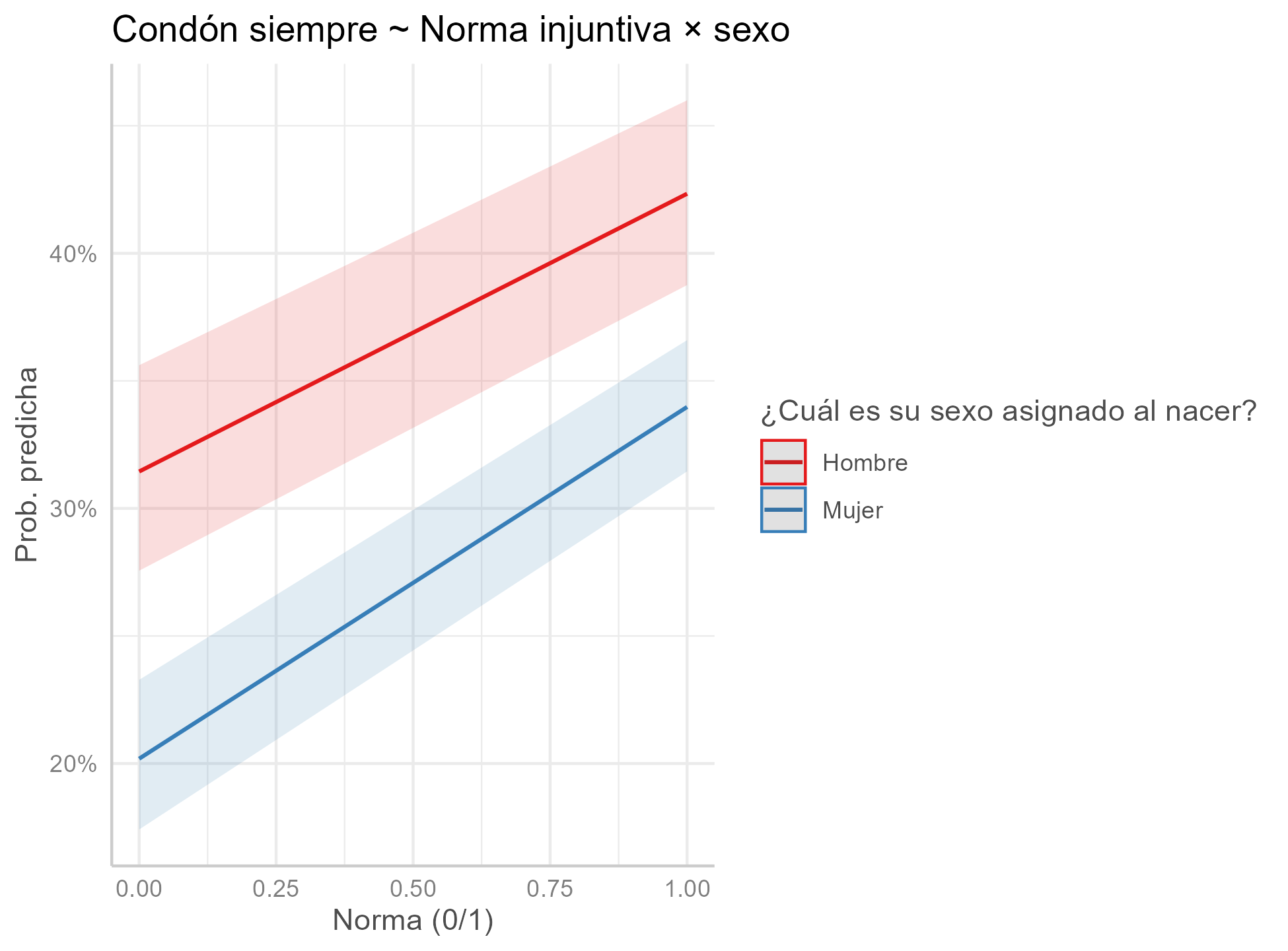


**Figure S5d. Predicted probability of consistent condom use by injunctive norm and sex. Shaded areas represent 95% confidence intervals. Red lines = men; blue lines = women. The x-axis represents the injunctive norm (0 = absence, 1 = presence), and the y-axis represents the predicted probability of always using condoms. (Original figure labels are in Spanish.)**


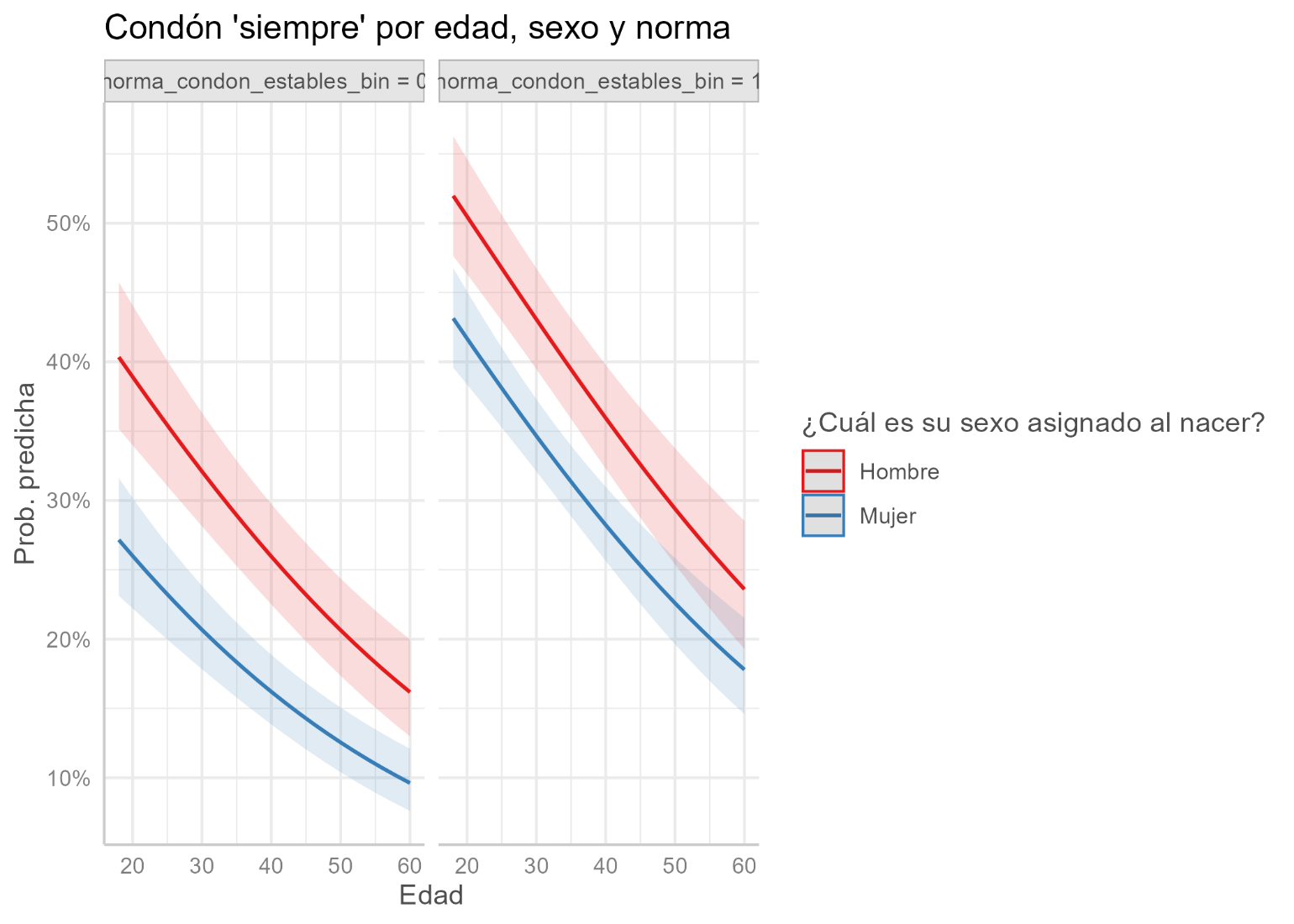


**Figure S5e. Predicted probability of consistent condom use by age, sex, and injunctive norm. Each panel represents the presence (1) or absence (0) of the injunctive norm related to condom use with stable partners. Shaded areas represent 95% confidence intervals. Red lines = men; blue lines = women. The x-axis represents age (years), and the y-axis represents the predicted probability of always using condoms. (Original figure labels are in Spanish.)**


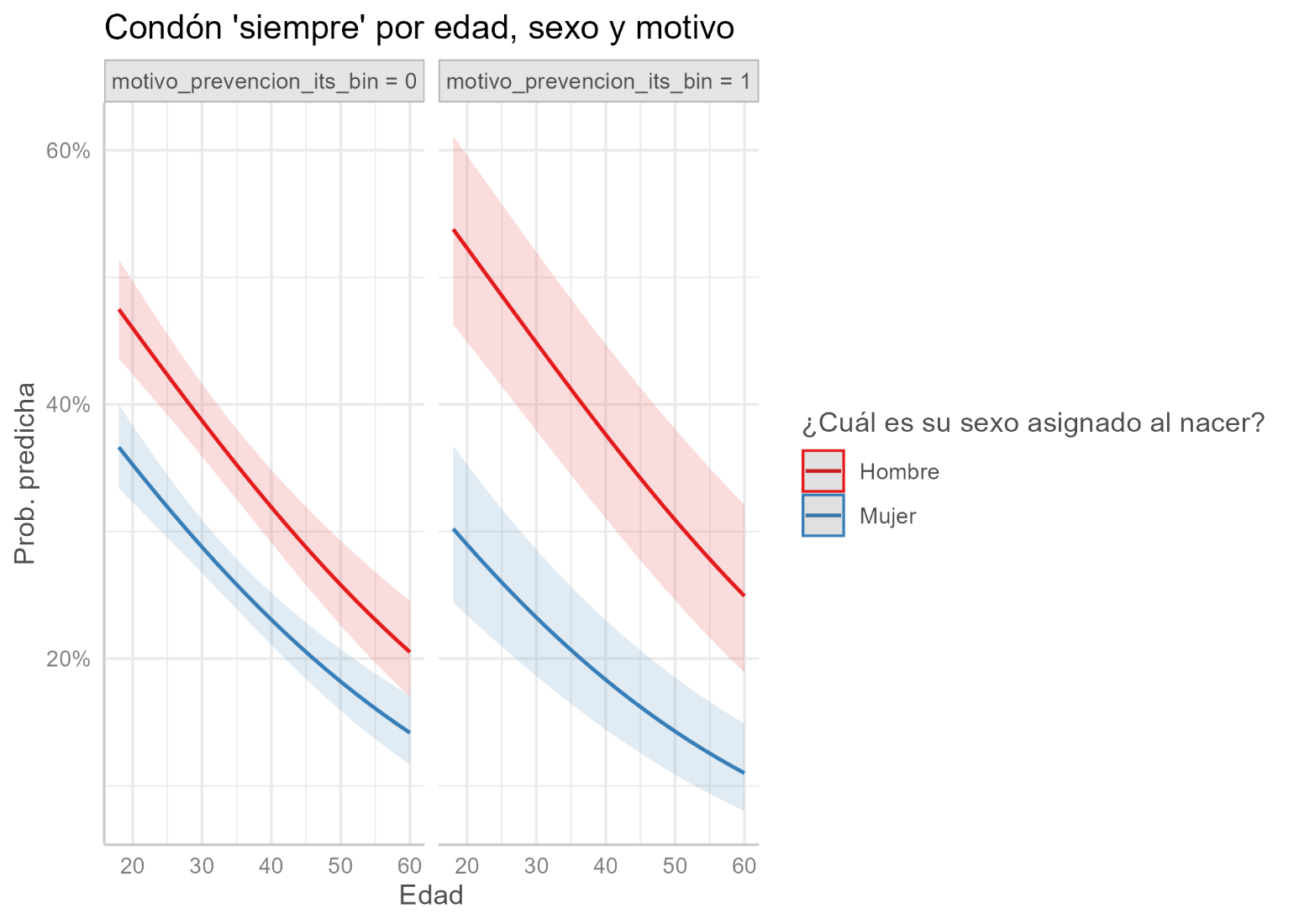


**Figure S5f. Predicted probability of consistent condom use by age, sex, and reason for condom use. Each panel represents participants who did not report (0) or did report (1) using condoms for HIV/STI prevention. Shaded areas represent 95% confidence intervals. Red lines = men; blue lines = women. The x-axis represents age (years), and the y-axis represents the predicted probability of always using condoms. (Original figure labels are in Spanish.)**


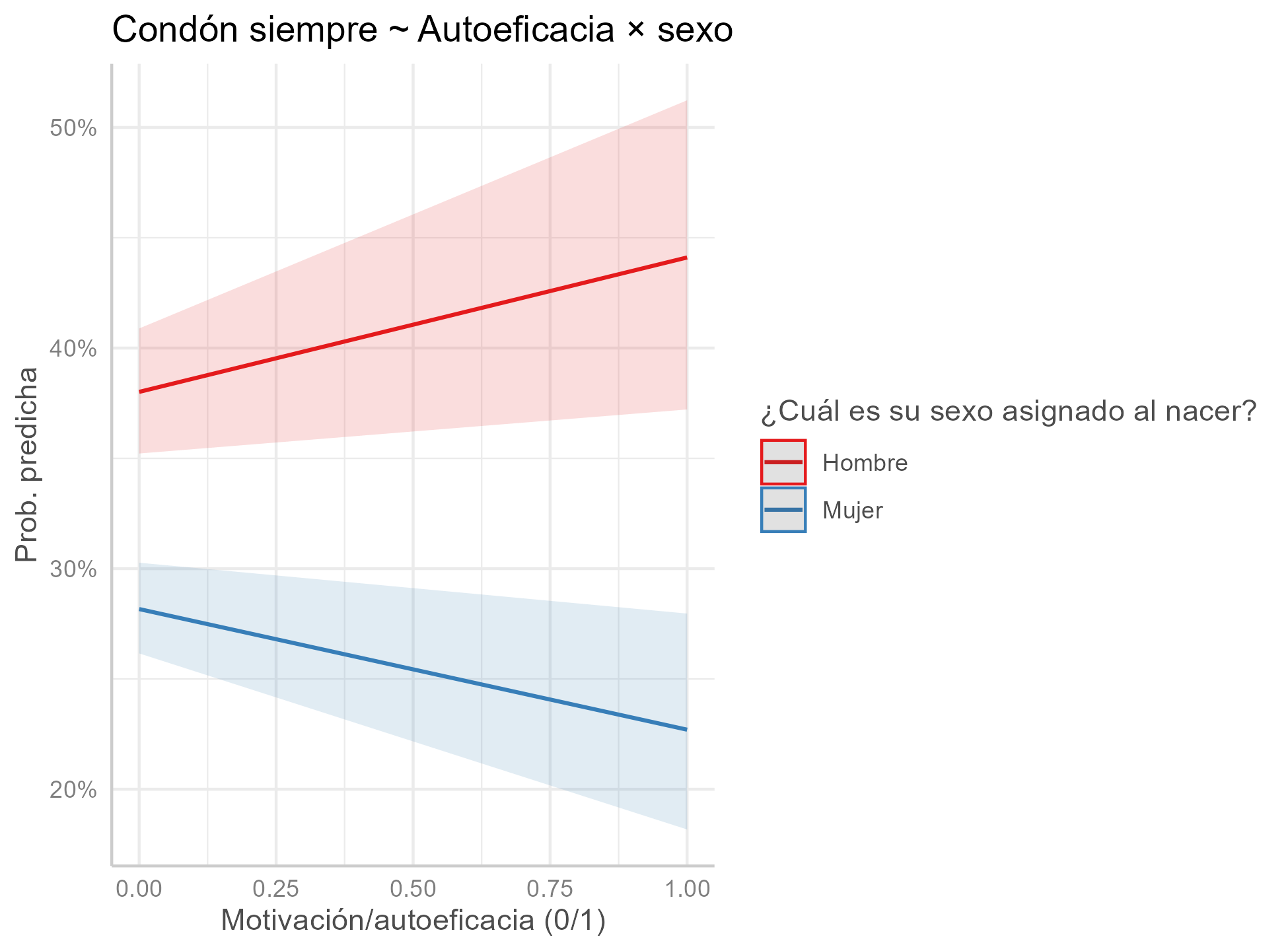


**Figure S5g. Predicted probability of consistent condom use by self-efficacy (motivation) and sex. Shaded areas represent 95% confidence intervals. Red lines = men; blue lines = women. The x-axis represents self-efficacy or motivation for condom use (0–1), and the y-axis represents the predicted probability of always using condoms. (Original figure labels are in Spanish.)**

Appendix S6. Exploratory sex interactions (SAGER compliance)

To assess potential effect modification, we estimated sex × determinant interaction terms in the multivariable condom-use model. The injunctive norm “condoms are necessary even with a stable partner” showed similar effects in men (aOR = 1.60; 95% CI: 1.27–2.02) and women (aOR = 2.03; 1.65–2.51; interaction p = 0.13). The self-efficacy proxy (“used condom to prevent HIV/STIs”) suggested a differential pattern (positive trend among men, negative among women; interaction p = 0.013), although estimates were imprecise and not robust in adjusted models. These exploratory interactions are reported here for transparency and were not included in the main text.

Table S6a. Reporting according to the SAGER guidelines

| **Section of article** | **Recommendation (SAGER)** | **Compliance in this study** | Page # |
| --- | --- | --- | --- |
| Title and abstract | If only one sex is included in the study, or if the results of the study are to be applied to only one sex or gender, the title and the abstract should specify the sex of animals or any cells, tissues and other material derived from these and the sex and gender of human participants. | NA, both sexes and genders included. | – |
|  |  |  |  |
| Introduction | Authors should report, where relevant, whether sex and/or gender differences may be expected. | Differences were expected and framed in research questions. | 6 |
|  |  |  |  |
| Methods | Authors should report how sex and gender were taken into account in the design of the study, whether they ensured adequate representation of males and females, and justify the reasons for any exclusion of males or females. | Both sexes adequately represented; sex and gender considered in variable selection and interactions. | 6 |
|  |  |  |  |
| Results | Where appropriate, data should be routinely presented disaggregated by sex and gender. Sex- and gender-based analyses should be reported regardless of positive or negative outcome. In clinical trials, data on withdrawals and dropouts should also be reported disaggregated by sex. | Outcomes disaggregated by sex; exploratory interactions tested and reported in Appendix S6. | 9 |
|  |  |  |  |
| Discussion | The potential implications of sex and gender on the study results and analyses should be discussed. If a sex and gender analysis was not conducted, the rationale should be given. Authors should further discuss the implications of the lack of such analysis on the interpretation of the results. | Implications of sex differences are discussed, noting consistent effects across genders except for self-efficacy. | 20 |
|  |  |  |  |

From: (Heidari S, Babor TF, De Castro P, Tort S, Curno M. Sex and Gender Equity in Research: rationale for the SAGER guidelines and recommended use. Research Integrity and Peer Review. 2016;1: 2. doi:10.1186/s41073-016-0007-6)

Appendix Supplementary Table S7. Consistency checks with P89 and P119.

S7. Consistency checks with P89 and P119.

| **Supplementary Table S7a. Auxiliary consistency checks — Overall (ENSSEX 2022–2023)** | | | |
| --- | --- | --- | --- |
| Indicator | Weighted % | 95% CI | Denominator (n) |
| Condom use at first post-separation sex (P89) | — | — | 2,228 |
| Any contraceptive at last sex (P119) | 36.0% | 34.3%–37.7% | 12,111 |
| Note S7a (overall). Percentages and 95% confidence intervals are survey-weighted using ENSSEX design weights (Taylor linearization; Wald CIs). For P89, no overall weighted prevalence was reported in the manuscript; we therefore display the unweighted denominator only (yes + no = 2,228). For P119, we report the overall weighted estimate 36.0% (95% CI 34.3–37.7) and its unweighted denominator (12,111 = 4,467 yes + 7,644 no). These indicators were specified as auxiliary consistency checks and were not entered as independent predictors. | | | |

| **Supplementary Table S7b. Auxiliary consistency checks — By sex (ENSSEX 2022–2023)** | | | | |
| --- | --- | --- | --- | --- |
| Indicator | Sex | Weighted % | 95% CI | Denominator (n) |
| Condom use at first post-separation sex (P89) | Male | 39.3% | 36.0%–42.6% | 821 |
| Condom use at first post-separation sex (P89) | Female | 33.3% | 30.9%–35.8% | 1,407 |
| Any contraceptive at last sex (P119) | Male | 38.1% | 36.7%–39.5% | 4,544 |
| Any contraceptive at last sex (P119) | Female | 35.5% | 34.4%–36.6% | 7,567 |
| **Note S7b (by sex).** Denominators are **unweighted** counts (e.g., P89: Male **821** = 327 yes + 494 no; Female **1,407** = 474 yes + 933 no). Percentages and 95% CIs are the **survey-weighted** estimates reported in Table 3. These measures are provided as **auxiliary checks of temporal coherence** and were **not** included as predictors in multivariable models. | | | | |

Appendix Supplementary Table S8. Full Distributions

Full response distributions and coding of behavioral determinants (ENSSEX 2022–2023). For each item we display original response categories (unweighted n; weighted % with 95% CI when available) and the recoding rule used for the manuscript’s binary indicators, alongside the corresponding survey-weighted prevalence (95% CI). Self-efficacy (p121) is restricted to respondents eligible for the condom-use items. Access to services combines p151 and p152. The social support index equals p34 (any vs none) + p55 (yes), yielding categories 0/1/2.

| **variable** | **item** | **category_code** | **category_label** | **n_unweighted** | **unweighted % (if available)** | **weighted % (95% CI) (if available)** |
| --- | --- | --- | --- | --- | --- | --- |
| p213 | PrEP awareness | 1 | Sí |  |  | 9.5 (8.8–10.3) |
| p213 | PrEP awareness | 2 | No |  |  | 83.5 (82.5–84.6) |
| p213 | PrEP awareness | 9 | Nr |  |  | 6.9 (6.1–7.7) |
| i_1_p33 | Condoms reduce women’s sexual pleasure | 1 | Muy en desacuerdo | 1309 | 6,4 |  |
| i_1_p33 | Condoms reduce women’s sexual pleasure | 2 | En desacuerdo | 6480 | 31,8 |  |
| i_1_p33 | Condoms reduce women’s sexual pleasure | 3 | Ni de acuerdo, ni en desacuerdo | 3411 | 16,7 |  |
| i_1_p33 | Condoms reduce women’s sexual pleasure | 4 | De acuerdo | 4579 | 22,5 |  |
| i_1_p33 | Condoms reduce women’s sexual pleasure | 5 | Muy de acuerdo | 509 | 2,5 |  |
| i_1_p33 | Condoms reduce women’s sexual pleasure | 8 | NS | 3758 | 18,4 |  |
| i_1_p33 | Condoms reduce women’s sexual pleasure | 9 | NR | 346 | 1,7 |  |
| i_1–i_6_p212 | HIV knowledge (i_1–i_6_p212) |  | Mean score (0–6), % correct items |  |  | 64.3% (95% CI: 63.5–65.1) |
| i_1_p33 | Condom belief: reduces women’s pleasure (i_1_p33) |  | Agree/strongly agree |  |  | 31.0% (95% CI: 29.8–32.2) |
| i_2_p33 | Condom belief: reduces men’s pleasure (i_2_p33) |  | Agree/strongly agree |  |  | 43.4% (95% CI: 41.9–44.9) |
| i_4_p33 | Condom belief: enhances sexual play (i_4_p33) |  | Agree/strongly agree |  |  | 30.7% (95% CI: 29.2–32.1) |
| p121 | Self-efficacy proxy (p121) |  | Used condom to prevent HIV/STIs |  |  | 13.0% (95% CI: 11.6–14.6) |
| p213 | PrEP awareness (p213) |  | Knows about PrEP |  |  | 10.2% (95% CI: 9.5–11.1) |
| i_3_p33 | Injunctive norm (i_3_p33) |  | Condoms are necessary even with stable partner (agree/strongly agree) |  |  | 50.6% (95% CI: 49.0–52.2) |
| i_5_p33 | Perceived cost barrier (i_5_p33) |  | Condoms are expensive (agree/strongly agree) |  |  | 21.5% (95% CI: 20.3–22.7) |
| p151/p152 | Access to services (p151/p152) |  | Ever consulted a health professional for sexual health |  |  | 49.9% (95% CI: 48.1–51.6) |
| p34+p55 | Social support index (p34+p55) |  | 0 / 1 / 2 |  |  | S0: 57.3% (95% CI: 55.9–58.7) / S1: 31.7% (95% CI: 30.5–32.9) / S2: 11.0% (95% CI: 10.2–11.8) |

Appendix Supplementary Table S9a-c. Full response distributions for descriptive and injunctive norms related to HIV/STI prevention behaviors (ENSSEX 2022–2023)

These tables summarize weighted percentages and 95% confidence intervals for key behavioral and normative indicators, as well as contrasts by injunctive norms.

**Table S9a.** *Descriptive norms related to HIV/STI prevention behaviors (ENSSEX 2022–2023).* Weighted prevalence and 95% confidence intervals for key behavioral indicators, including consistent condom use, contraceptive use at last sex, and family sexuality communication

| **Table S9a. Descriptive norms – Prevalence (ENSSEX 2022–2023)** | | |
| --- | --- | --- |
| **Indicator** | **Weighted %** | **95% CI** |
| Consistent condom use (P73) | 15.5% | 14.5%–16.5% |
| Any contraceptive at last sex (P119) | 36.0% | 34.3%–37.7% |
| Family sexuality talk (P34: any) | 68.8% | 67.7%–69.9% |
| Note. Percentages and 95% CIs are survey-weighted using ENSSEX design weights; CIs via Taylor linearization (Wald). Items include P73 (condom “always” in past year), P119 (contraceptive at last sex), and P34 (family talk about sexuality in childhood). Denominators reflect valid (non-missing) responses. | | |

**Table S9b.** *Injunctive norms related to HIV/STI prevention behaviors (ENSSEX 2022–2023).*Weighted prevalence and 95% confidence intervals for injunctive beliefs and perceived norms regarding condom use and sexual health discussions

| **Table S9b. Injunctive norms – Prevalence (ENSSEX 2022–2023)** | | |
| --- | --- | --- |
| **Indicator** | **Weighted %** | **95% CI** |
| Injunctive: condoms necessary even with stable partner | 50.6% | 49.0%–52.2% |
| Belief: condoms reduce women's pleasure (agree) | 21.3% | 20.2%–22.4% |
| Belief: condoms reduce men's pleasure (agree) | 21.4% | 20.4%–22.5% |
| Belief: condoms enhance sexual play (agree) | 19.8% | 18.7%–20.8% |
| Discussed STI prevention before first sex (yes) | 1.5% | 1.2%–1.9% |
| Note. Percentages and 95% CIs are survey-weighted using ENSSEX design weights; CIs via Taylor linearization (Wald). Items include i_3_p33 (condoms necessary even with stable partner), i_1_p33 / i_2_p33 (beliefs on condoms reducing/enhancing pleasure), and discussion of STI prevention before first sex. Denominators reflect valid (non-missing) responses. | | |

**Table S9c.***Contrasts by injunctive norm: “It is necessary to use condoms even with a stable partner” (ENSSEX 2022–2023).*Weighted percentages and 95% confidence intervals comparing consistent condom use and HIV/STI testing across groups differing in endorsement of injunctive norm

| **Table S9c. Contrasts by injunctive norm (if condoms necessary even with stable partner)** | | | |
| --- | --- | --- | --- |
| **Outcome** | **Group** | **Weighted %** | **95% CI** |
| Consistent condom use | Other | 9.0% | 7.7%–10.2% |
| Consistent condom use | Agree/strongly agree | 20.5% | 18.7%–22.2% |
| HIV/STI testing (12m) | Other | 21.3% | 19.6%–23.1% |
| HIV/STI testing (12m) | Agree/strongly agree | 26.9% | 25.0%–28.8% |
| Note. Percentages and 95% CIs are survey-weighted using ENSSEX design weights; CIs via Taylor linearization (Wald). “Agree/strongly agree” refers to item i_3_p33 (“It is necessary to use condoms even with a stable partner”); “Other” pools neutral, disagree, and strongly disagree. Outcomes: Consistent condom use (P73, “always” vs. other) and HIV/STI testing (12m) (tested in past 12 months). Denominators reflect valid (non-missing) responses for each outcome and grouping. | | | |

Appendix Supplementary Table S10 a-b. Full Distributions

Table S10a-b

| **S10a — By sex: Consistent condom use (Men)** | | | |
| --- | --- | --- | --- |
| Sex | Determinant | OR (95% CI) | p-value |
| Men | Access to services | 0.89 (0.68–1.15) | 0.372 |
| Men | Belief: condoms enhance sexual play (agree) | 1.29 (1.02–1.62) | 0.033 |
| Men | Belief: condoms reduce men's pleasure (agree) | 0.83 (0.67–1.03) | 0.084 |
| Men | Belief: condoms reduce women's pleasure (agree) | 0.83 (0.66–1.04) | 0.107 |
| Men | HIV knowledge (per point) | 1.14 (1.04–1.24) | 0.004 |
| Men | Injunctive norm (agree/strongly agree) | 2.59 (2.04–3.29) | <0.001 |
| Men | Perceived cost barrier (agree) | 0.74 (0.55–1.01) | 0.056 |
| Men | PrEP awareness | 1.77 (1.27–2.47) | <0.001 |
| Men | Self-efficacy: used condom to prevent HIV/STIs | 1.14 (0.81–1.60) | 0.464 |
|  |  |  |  |
| **S10a— By sex: Consistent condom use (Women)** | | | |
| Sex | Determinant | OR (95% CI) | p-value |
| Women | Access to services | 1.09 (0.87–1.38) | 0.443 |
| Women | Belief: condoms enhance sexual play (agree) | 1.33 (1.06–1.67) | 0.013 |
| Women | Belief: condoms reduce men's pleasure (agree) | 0.68 (0.53–0.87) | 0.002 |
| Women | Belief: condoms reduce women's pleasure (agree) | 0.55 (0.43–0.69) | <0.001 |
| Women | HIV knowledge (per point) | 0.98 (0.90–1.07) | 0.608 |
| Women | Injunctive norm (agree/strongly agree) | 3.00 (2.31–3.90) | <0.001 |
| Women | Perceived cost barrier (agree) | 0.87 (0.68–1.12) | 0.295 |
| Women | PrEP awareness | 1.34 (1.04–1.72) | 0.023 |
| Women | Self-efficacy: used condom to prevent HIV/STIs | 0.91 (0.63–1.32) | 0.624 |
|  |  |  |  |
| **S10b — By sex: HIV/STI testing (Men)** | | | |
| Sex | Determinant | OR (95% CI) | p-value |
| Men | Access to services | 1.90 (1.60–2.25) | <0.001 |
| Men | Belief: condoms enhance sexual play (agree) | 1.48 (1.18–1.84) | <0.001 |
| Men | Belief: condoms reduce men's pleasure (agree) | 0.98 (0.82–1.19) | 0.870 |
| Men | Belief: condoms reduce women's pleasure (agree) | 1.28 (1.06–1.54) | 0.010 |
| Men | HIV knowledge (per point) | 1.21 (1.12–1.30) | <0.001 |
| Men | Injunctive norm (agree/strongly agree) | 1.34 (1.03–1.74) | 0.027 |
| Men | Perceived cost barrier (agree) | 0.77 (0.57–1.05) | 0.099 |
| Men | PrEP awareness | 2.61 (1.91–3.55) | <0.001 |
| Men | Self-efficacy: used condom to prevent HIV/STIs | 1.80 (1.28–2.52) | <0.001 |
|  |  |  |  |
| **S10b — By sex: HIV/STI testing (Women)** | | | |
| Sex | Determinant | OR (95% CI) | p-value |
| Women | Access to services | 2.28 (1.86–2.80) | <0.001 |
| Women | Belief: condoms enhance sexual play (agree) | 1.07 (0.92–1.25) | 0.379 |
| Women | Belief: condoms reduce men's pleasure (agree) | 1.14 (0.96–1.34) | 0.127 |
| Women | Belief: condoms reduce women's pleasure (agree) | 0.99 (0.84–1.18) | 0.950 |
| Women | HIV knowledge (per point) | 1.16 (1.09–1.23) | <0.001 |
| Women | Injunctive norm (agree/strongly agree) | 1.26 (1.02–1.54) | 0.028 |
| Women | Perceived cost barrier (agree) | 0.97 (0.81–1.16) | 0.745 |
| Women | PrEP awareness | 1.82 (1.52–2.17) | <0.001 |
| Women | Self-efficacy: used condom to prevent HIV/STIs | 1.68 (1.21–2.32) | 0.002 |

Appendix Supplementary Table S11 a-b. Full Distributions

Table S11

| **S11a — Association of p89/p119 with Consistent condom use (vs p73)** |  |  |
| --- | --- | --- |
| Predictor (binary yes=1) | OR (95% CI) | p-value |
| Condom at first sex after separation (p89) | 5.85 (4.01–8.53) | <0.001 |
| Condom at last sex (p119) | 9.71 (7.90–11.94) | <0.001 |
|  |  |  |
| **S11b — p89 as alternate outcome (bivariate ORs)** |  |  |
| Determinant | OR (95% CI) | p-value |
| HIV knowledge (per point) | 0.99 (0.88–1.11) | 0.825 |
| PrEP awareness | 1.55 (1.10–2.19) | 0.013 |
| Belief: condoms reduce women's pleasure (agree) | 0.66 (0.45–0.98) | 0.040 |
| Belief: condoms reduce men's pleasure (agree) | 0.73 (0.52–1.02) | 0.068 |
| Belief: condoms enhance sexual play (agree) | 1.39 (1.00–1.95) | 0.053 |
| Self-efficacy: used condom to prevent HIV/STIs | 1.18 (0.73–1.91) | 0.500 |
| Injunctive norm (agree/strongly agree) | 2.49 (1.81–3.42) | <0.001 |
| Perceived cost barrier (agree) | 1.08 (0.82–1.43) | 0.563 |
| Access to services | 0.97 (0.72–1.30) | 0.823 |

Appendix Supplementary Table S12

Table S12

| **S12 — p119 as alternate outcome (bivariate ORs)** |  |  |
| --- | --- | --- |
| **Determinant** | OR (95% CI) | p-value |
| HIV knowledge (per point) | 1.04 (0.97–1.10) | 0.269 |
| PrEP awareness | 1.26 (1.06–1.51) | 0.010 |
| Belief: condoms reduce women's pleasure (agree) | 0.73 (0.64–0.83) | <0.001 |
| Belief: condoms reduce men's pleasure (agree) | 0.78 (0.68–0.89) | <0.001 |
| Belief: condoms enhance sexual play (agree) | 1.08 (0.94–1.24) | 0.298 |
| Self-efficacy: used condom to prevent HIV/STIs | 0.68 (0.51–0.90) | 0.008 |
| Injunctive norm (agree/strongly agree) | 2.02 (1.75–2.33) | <0.001 |
| Perceived cost barrier (agree) | 0.91 (0.77–1.06) | 0.234 |
| Access to services | 0.95 (0.83–1.08) | 0.424 |

Appendix Supplementary Table S13. Survey-weighted crude odds ratios (bivariate associations) for consistent condom use and HIV/STI testing (ENSSEX 2022–2023)

Table S13. Survey-weighted crude odds ratios (bivariate associations)

|  | **Determinant** | OR (95% CI) - Consistent condom use | p-value - Consistent condom use | OR (95% CI) - HIV/STI testing | p-value - HIV/STI testing |
| --- | --- | --- | --- | --- | --- |
| **Individual determinants** | HIV knowledge (per point) | 1.06 (0.99–1.13) | .078 | 1.18 (1.13–1.23) | <.001 |
|  | PrEP awareness | 1.53 (1.24–1.89) | <.001 | 2.14 (1.81–2.52) | <.001 |
|  | Belief: condoms reduce women's pleasure (agree) | 0.72 (0.61–0.84) | <.001 | 1.09 (0.96–1.23) | .169 |
|  | Belief: condoms reduce men's pleasure (agree) | 0.78 (0.67–0.91) | .002 | 1.03 (0.91–1.17) | .630 |
|  | Belief: condoms enhance sexual play (agree) | 1.30 (1.09–1.55) | .003 | 1.25 (1.10–1.42) | <.001 |
|  | Self-efficacy: used condom to prevent HIV/STIs | 1.08 (0.84–1.39) | .553 | 1.66 (1.33–2.07) | <.001 |
| **Socio-structural determinants** | Injunctive norm (agree/strongly agree) | 2.61 (2.18–3.12) | <.001 | 1.36 (1.17–1.58) | <.001 |
|  | Perceived cost barrier (agree) | 0.79 (0.64–0.97) | .028 | 0.90 (0.77–1.05) | .172 |
|  | Access to services | 0.83 (0.70–0.97) | .018 | 2.14 (1.91–2.41) | <.001 |
|  | Social support (any vs none) | 2.39 (2.07–2.75) | <.001 | 1.55 (1.38–1.73) | <.001 |
| Note. Survey-weighted crude odds ratios (OR) from separate bivariate logistic regressions that account for stratification, clustering, and sampling weights. Outcomes: Consistent condom use (p73 = “always” vs other) and HIV/STI testing (p208 = ≥1 test in the past 12 months). Predictor coding: HIV knowledge = per 1-point increase in 0-6 score (items i_1–i_6_p212); PrEP awareness (p213 = yes); beliefs from p33 dichotomized “agree/strongly agree” vs other: reduces women’s pleasure (i_1_p33), reduces men’s pleasure (i_2_p33), enhances sexual play (i_4_p33); self-efficacy (p121a = yes: used condom to prevent HIV/STIs); injunctive norm (i_3_p33 agree/strongly agree); perceived cost barrier (i_5_p33 agree/strongly agree); access to services (p151/p152 = yes: ever consulted a health professional for sexual-health concerns); social support (index from p34 and p55; any vs none). Don’t-know/no-response were coded as missing (listwise deletion). 95% CIs are model-based; p-values are from Wald tests. OR > 1 indicates higher odds of the outcome. Abbreviations: OR, odds ratio; CI, confidence interval; PrEP, pre-exposure prophylaxis. | | | | | |
